# Supplementary material for: SAR Study and Molecular Mechanism Investigation of Novel Naphthoquinone-furan-2-cyanoacryloyl Hybrids with Antitumor Activity
Source: Pharmaceutics. 2022 Oct 1;14(10):2104. doi: 10.3390/pharmaceutics14102104 (PMC9609996; doi:10.3390/pharmaceutics14102104)
Supplement: Supplementary file 1 [file pharmaceutics-14-02104-s001.zip › pharmaceutics-1842667-supplementary.pdf]

SUPPLEMENTARY MATERIAL

SAR Study and Molecular Mechanism Investigation of Novel Naphthoquinone-furan-2-cyanoacryloyl Hybrids with Antitumor Activity

Pingxian Liu [1,2,†], Dongmei Fan [1,2,†], Wenliang Qiao [3,†], Xinlian He [1,2,4], Lidan Zhang [1,2,4], Yunhan Jiang [1,2,5] and Tao Yang [1,2,4,*]

1 Laboratory of Human Diseases and Immunotherapies, West China Hospital, Sichuan University, Chengdu 610041, China.

2 Institute of Immunology and Inflammation, Frontiers Science Center for Disease-Related Molecular Network, West China Hospital, Sichuan University, Chengdu 610041, China

3 Laboratory of Lung Cancer, Lung Cancer Center, West China Hospital, Sichuan University, Chengdu 610041, China.

4 State Key Laboratory of Biotherapy and Cancer Center, West China Hospital, Sichuan University, Chengdu 610041, China

5 Department of Cardiovascular Surgery, West China Hospital, Sichuan University, Chengdu 610041, China

CONTENTS:

Table S1. Binding free energies (ΔG_{bind}^{cal}) for **5c**/STAT3 and **SI-109**/STAT3 systems.

Figure S1. Root means square deviation (RMSD) value of heavy atoms of backbone for protein receptor along 100 ns MD simulation for **5c**/STAT3 and **SI-109**/STAT3 complex systems.

Figure S2. Binding model for **SI-109** with STAT3.

Figure S3. Binding model for **5c** with STAT3.

^{1}H NMR, ^{13}C NMR, IR and HRMS Spectrum for **2, 4a-4i, 5a-5g, 6, 6a, 8a-8i and 9a-9c.**

Table S1. Binding free energies (ΔG_{bind}^{cal}) for **5c**/STAT3 and **SI-109**/STAT3 systems.

Energy	**5c**		**SI-109**[a]	
	Average	Std. Dev.	Average	Std. Dev.
ΔE_{vdW}	-34.68	2.70	-43.18	5.67
ΔE_{ele}	-34.64	4.75	-225.27	24.77
ΔE_{GB}	40.41	4.70	219.50	21.49
ΔE_{surf}	-4.96	0.29	-6.71	0.46
ΔE_{gas}	-69.32	5.46	-268.45	25.14
ΔE_{solv}	35.45	4.57	212.79	21.39
ΔG_{bind}^{cal}	-33.87	3.52	-55.66	8.85

The binding free energies ($\mathbf{\Delta G_{bind}^{cal}}$) for **5c**/STAT3 and **SI-109**/STAT3 complex and decomposition to electrostatic interaction ($\mathbf{\Delta E_{ele}}$), van der Walls interaction ($\mathbf{\Delta E_{vdW}}$), solvation free energies ($\mathbf{\Delta E_{GB}}$), and non-polar free energies ($\mathbf{\Delta E_{surf}}$). Energy values are presented in kcal/mol. Uncertainties (Std. Dev.) were calculated as the root mean square error for all frames extracted from the trajectories. [a] Ligand contained in the crystal structure of 6NUQ.

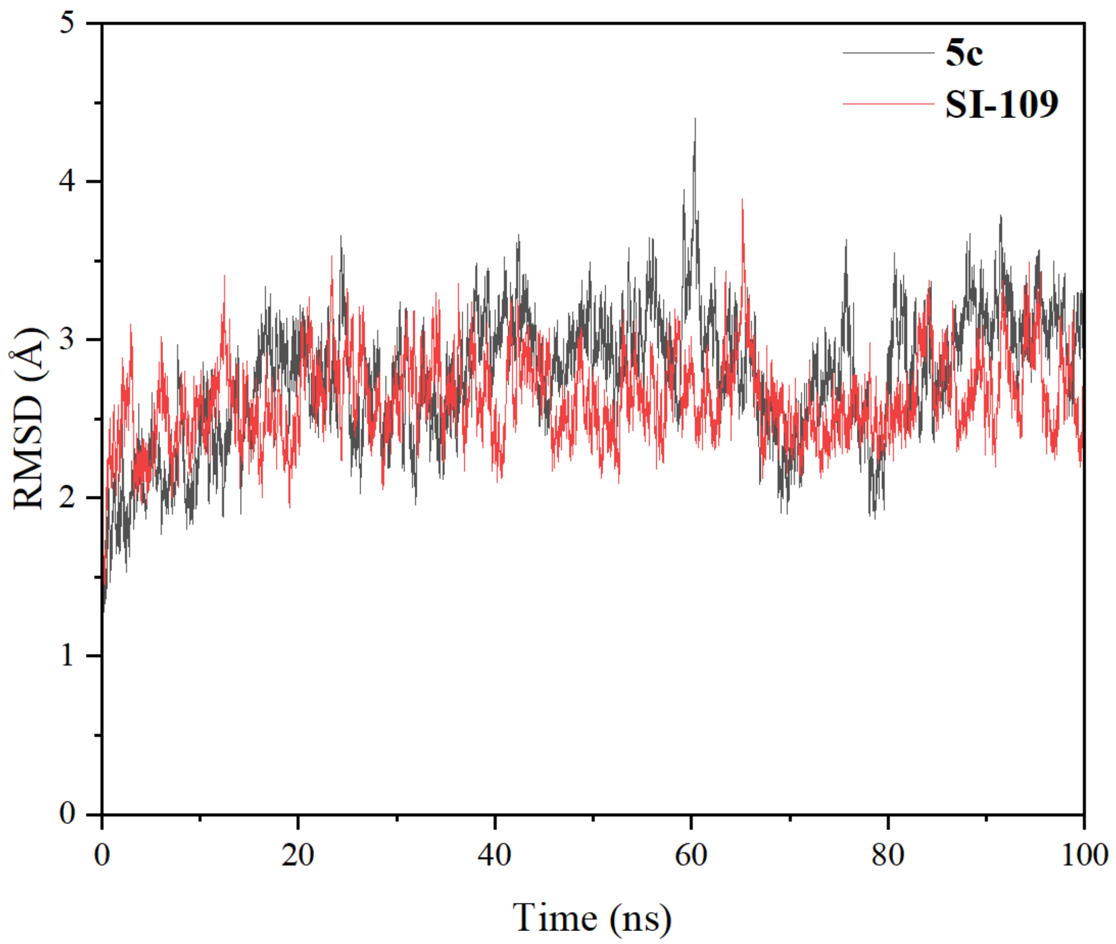

Figure S1. Root means square deviation (RMSD) value of heavy atoms of backbone for protein receptor along 100 ns MD simulation for **5c**/STAT3 and **SI-109**/STAT3 complex systems.

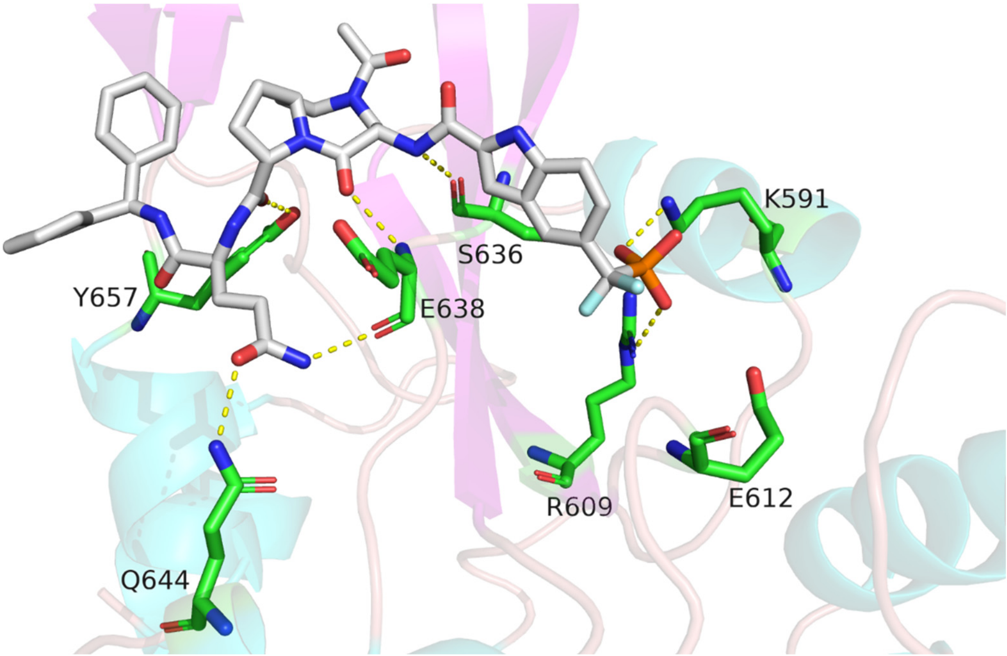

Figure S2. Binding model for **SI-109** with STAT3.

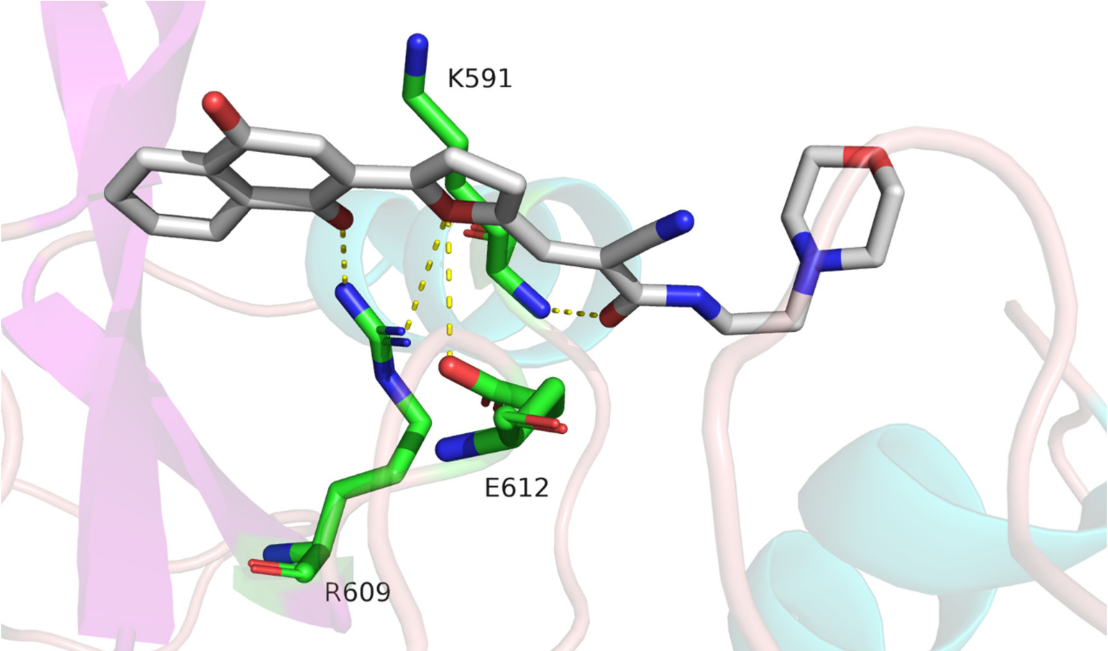

Figure S3. Binding model for **5c** with STAT3.

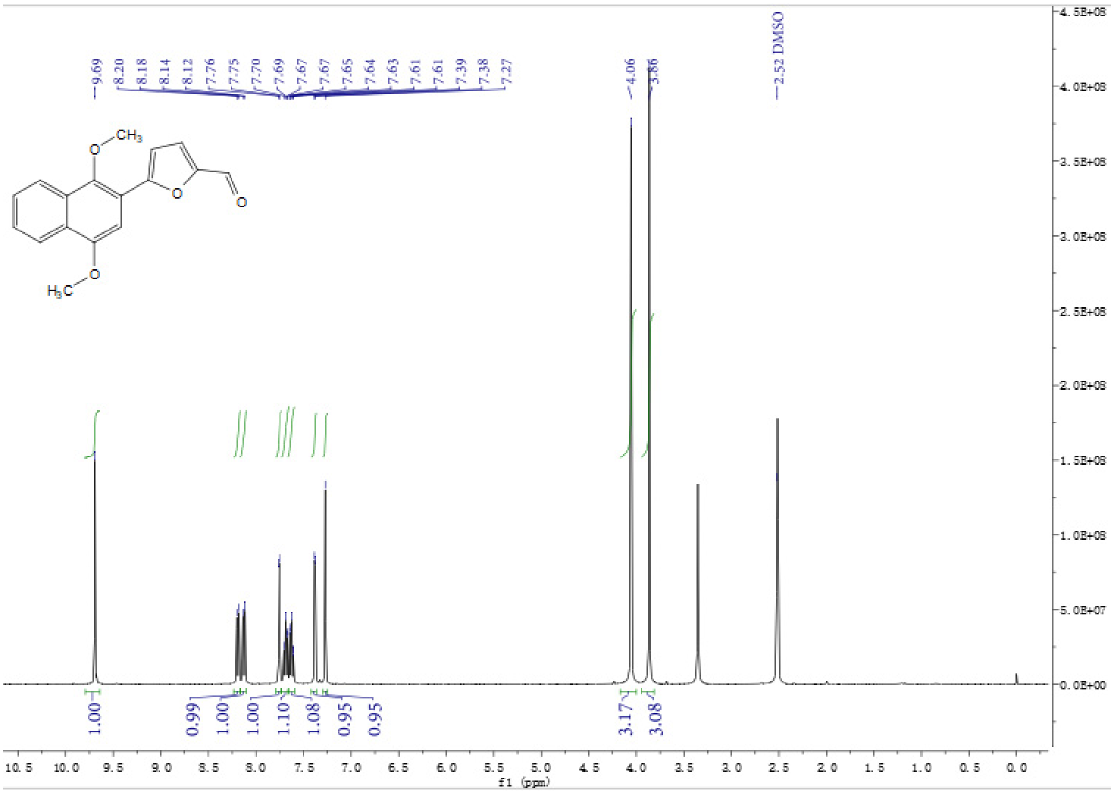

^{1}H-NMR of compound **2**

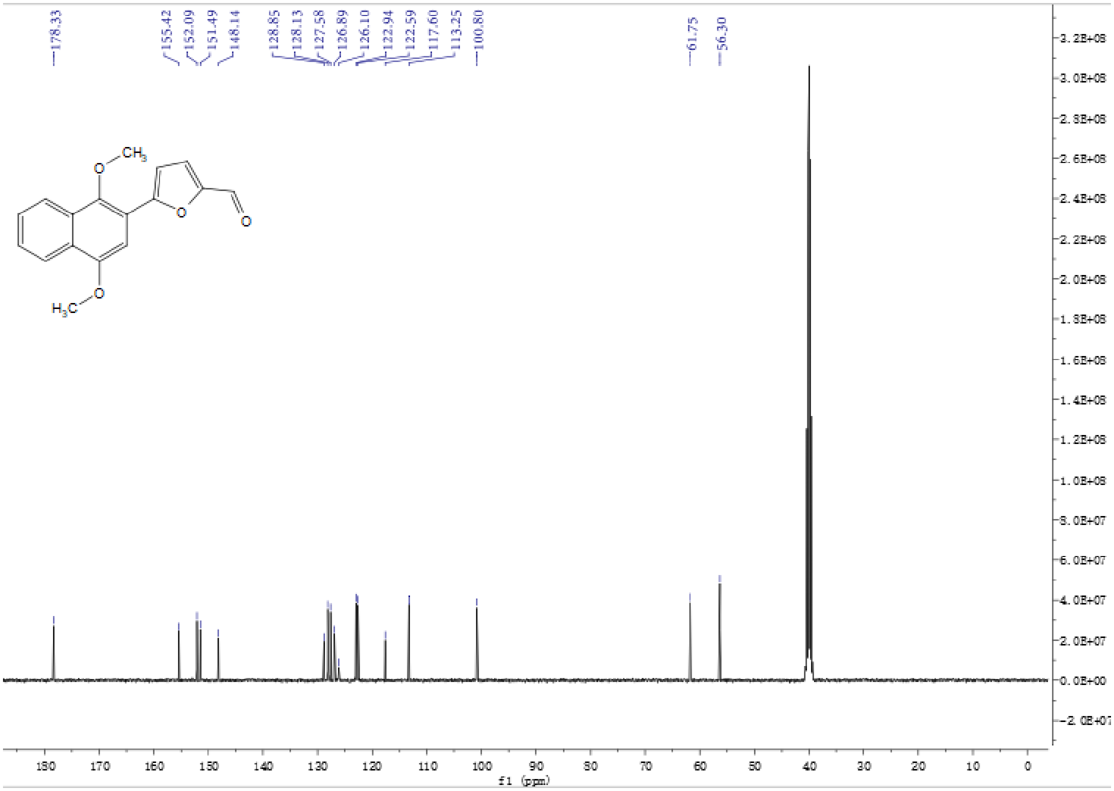

^{13}C-NMR of compound **2**

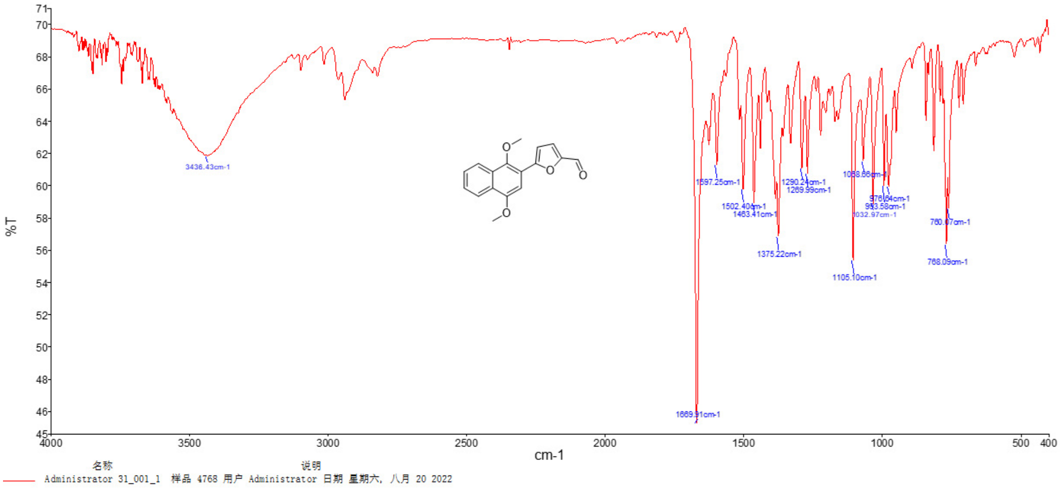

IR of compound **2**

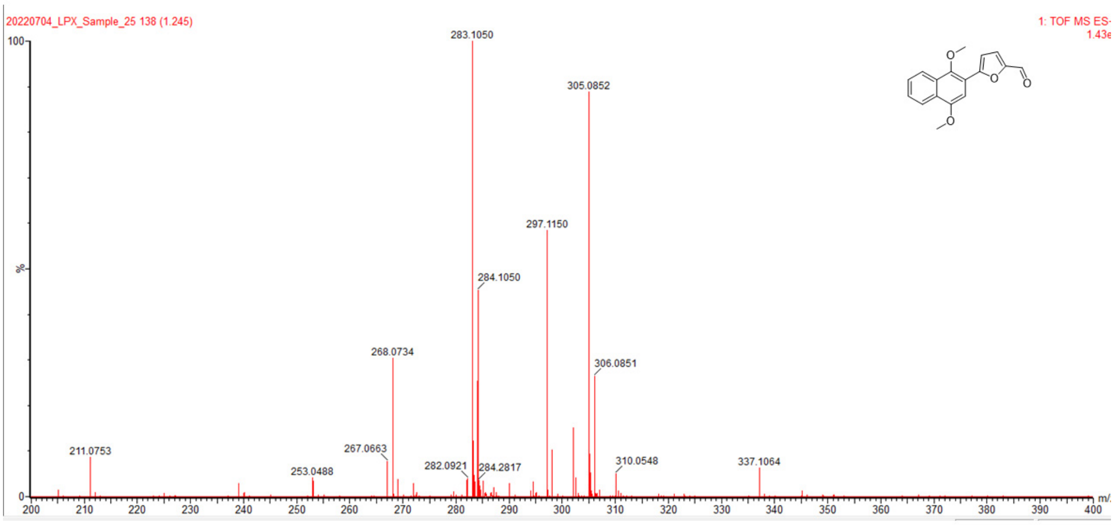

HRMS of compound **2**

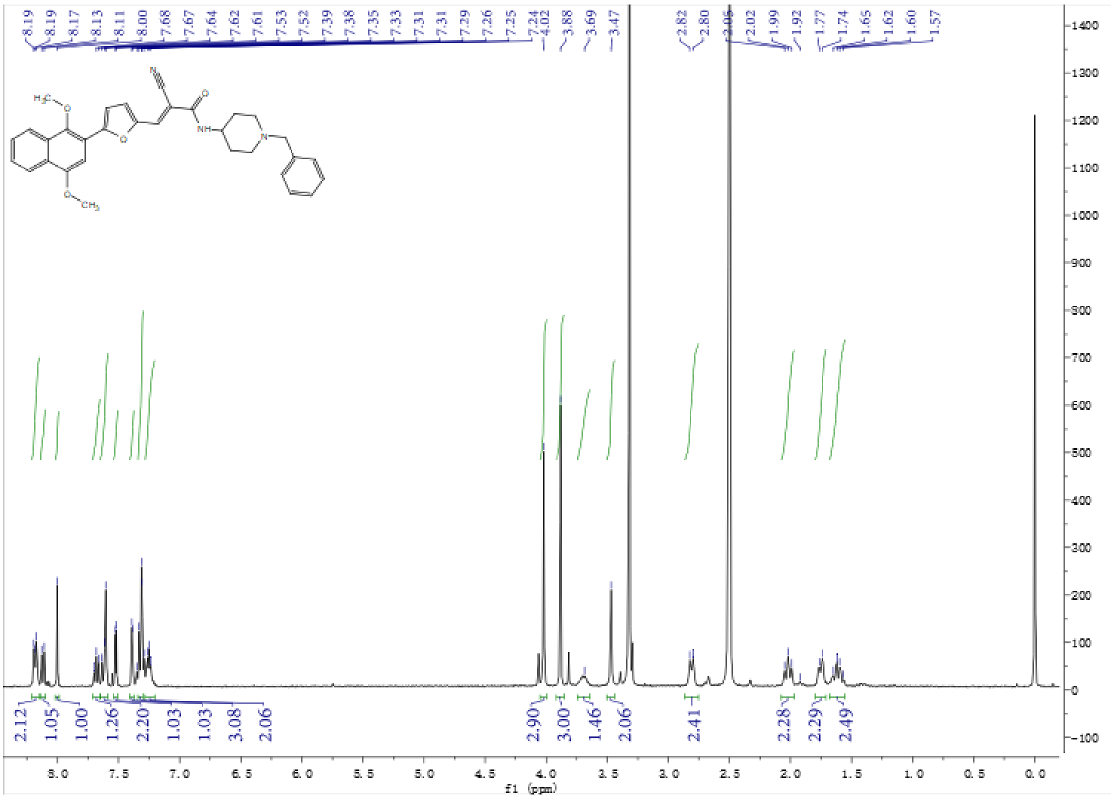

^{1}H-NMR of compound **4a**

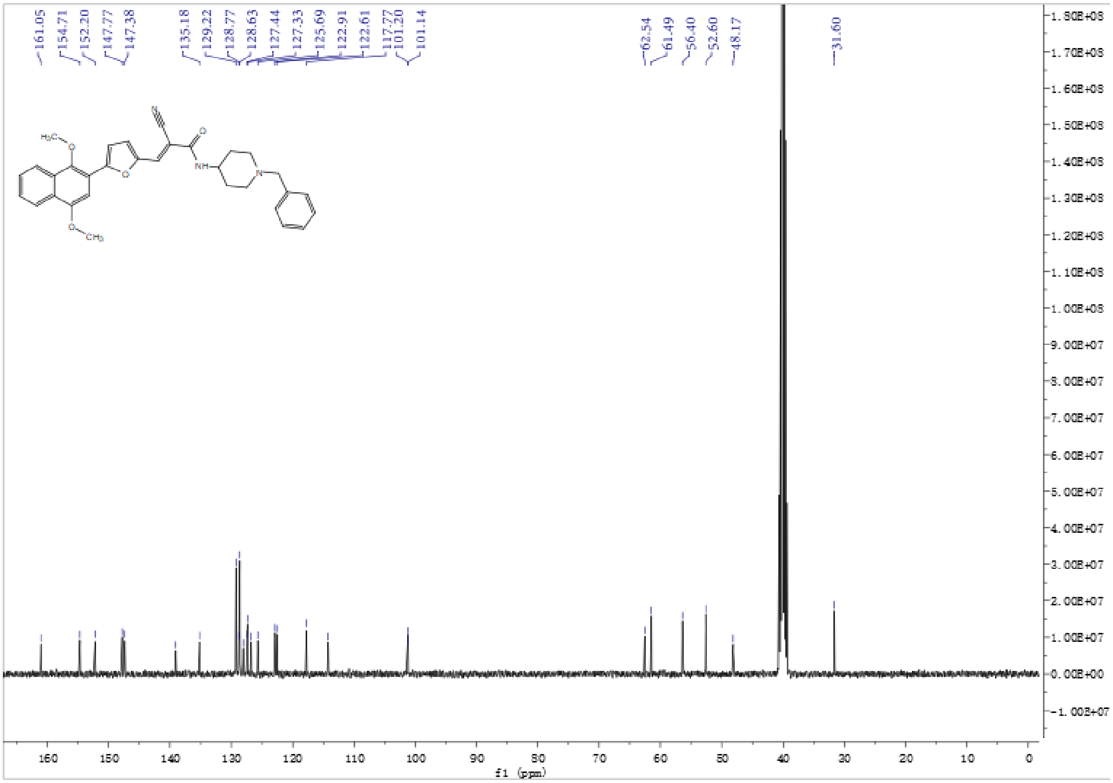

^{13}C-NMR of compound **4a**

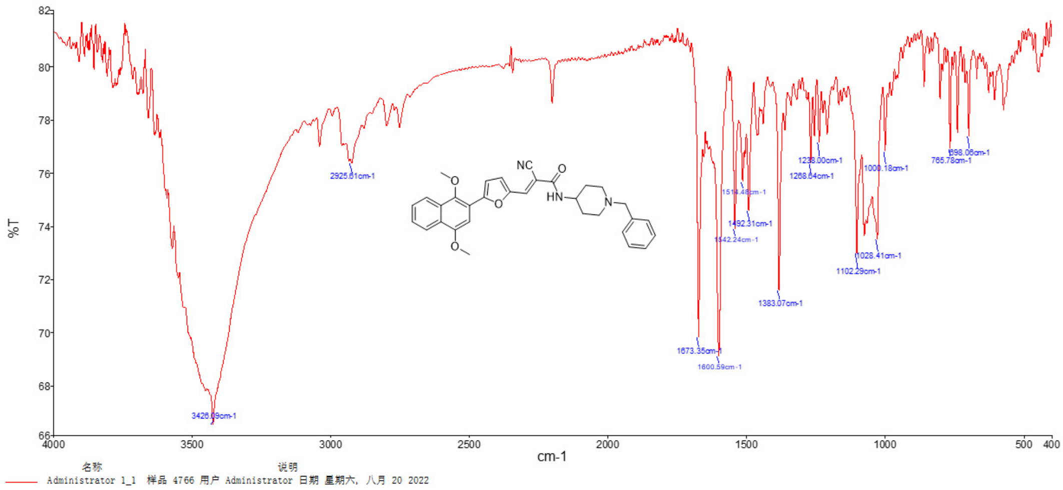

IR of compound **4a**

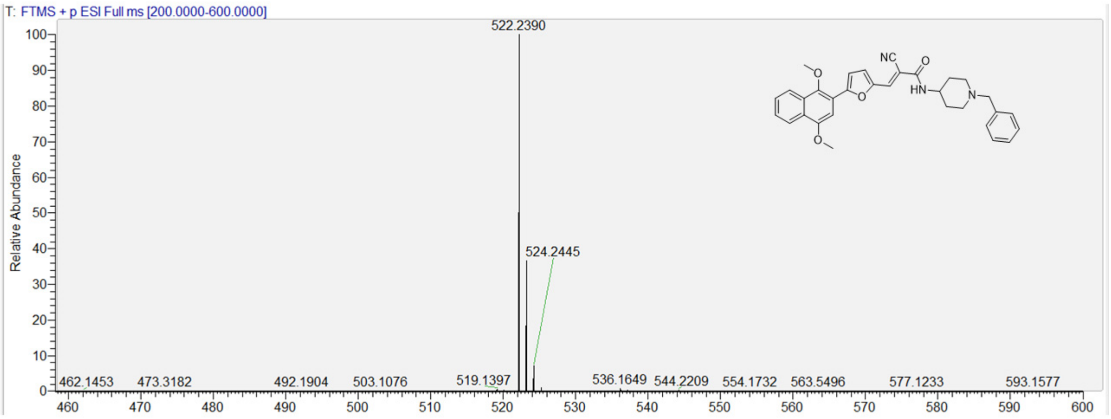

HRMS of compound **4a**

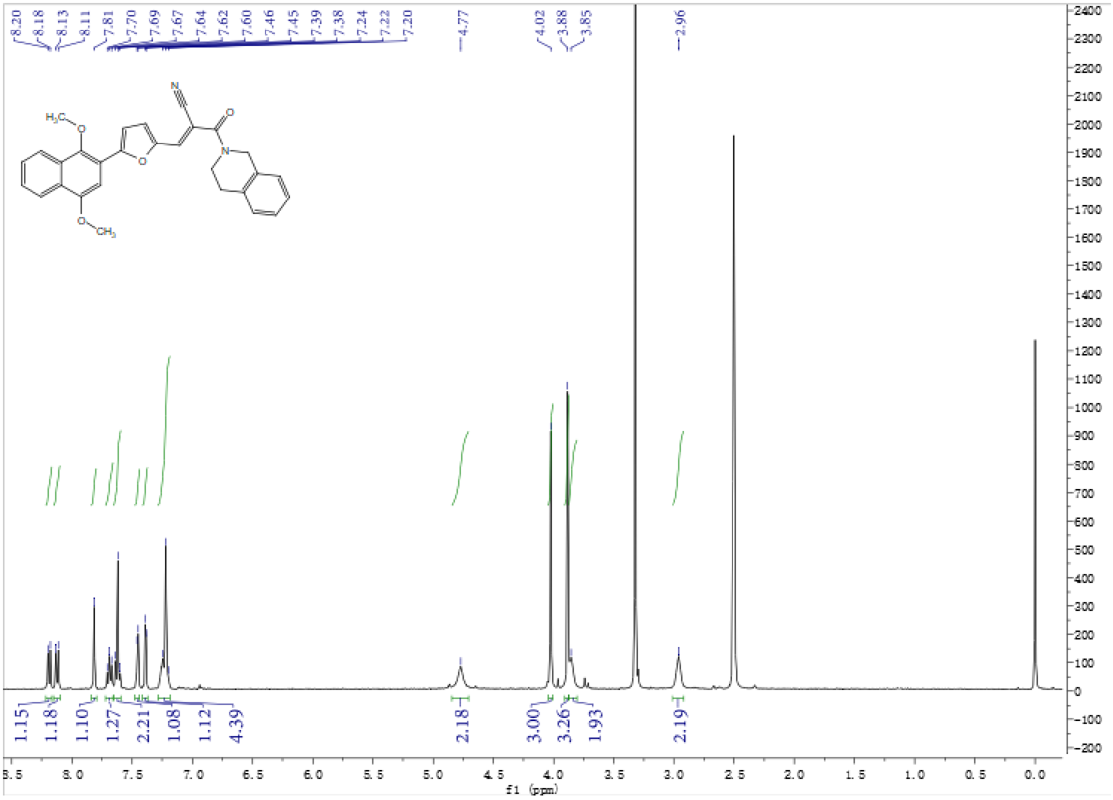

^{1}H-NMR of compound **4b**

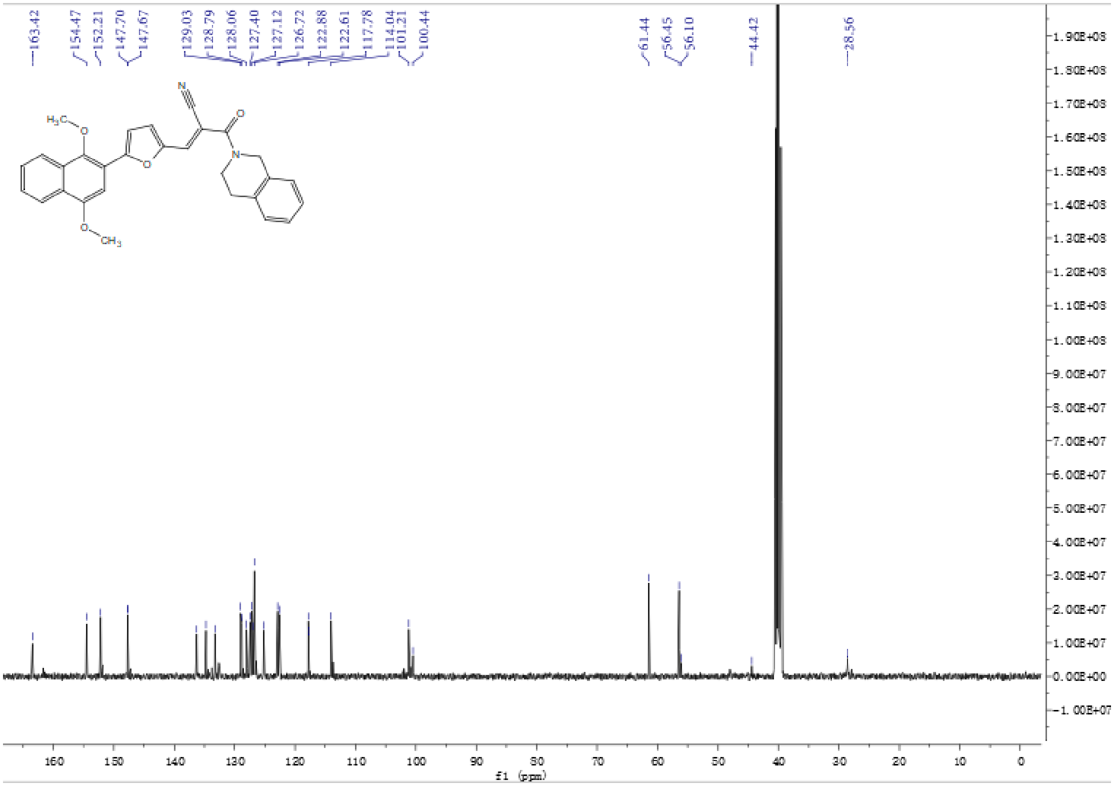

^{13}C-NMR of compound **4b**

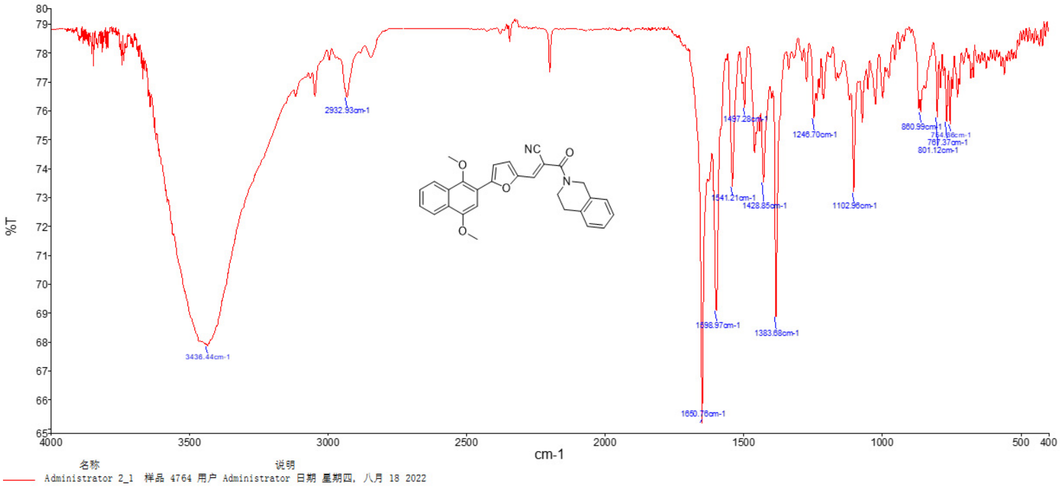

IR of compound **4b**

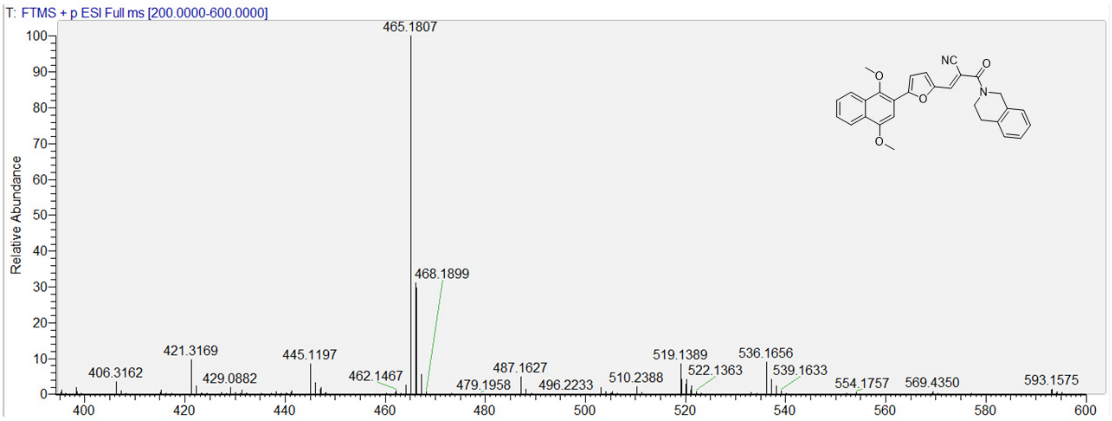

HRMS of compound **4b**

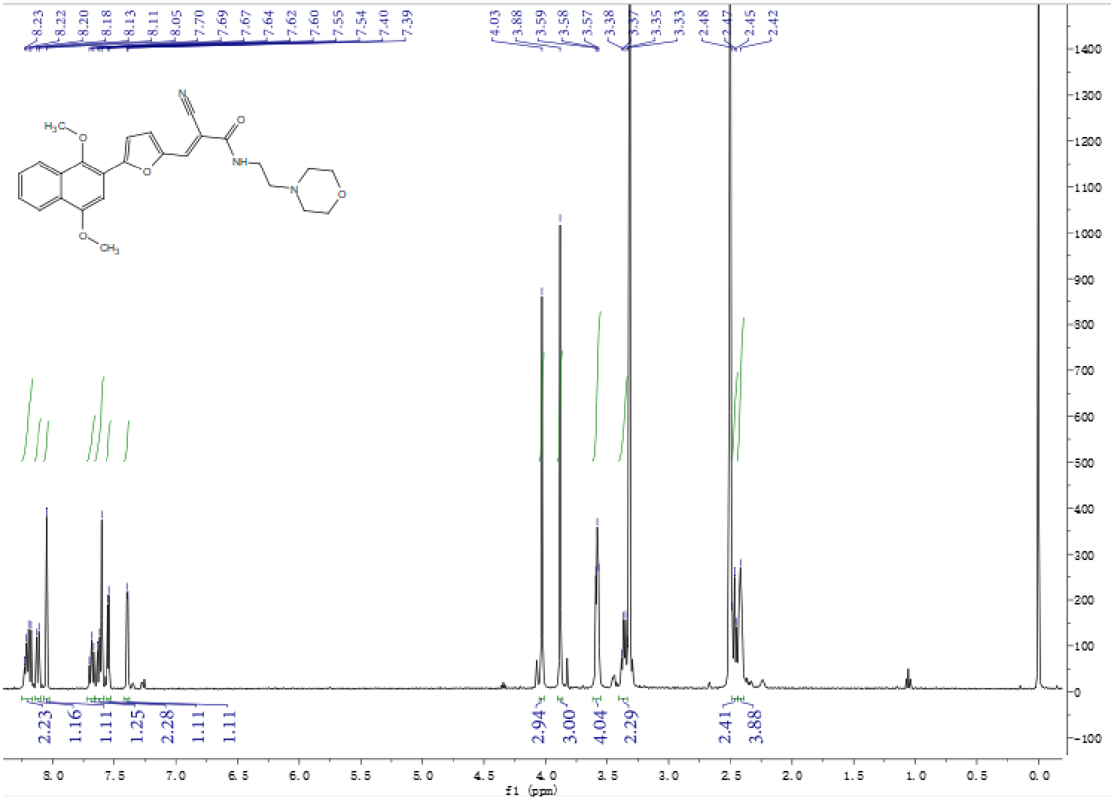

^{1}H-NMR of compound **4c**

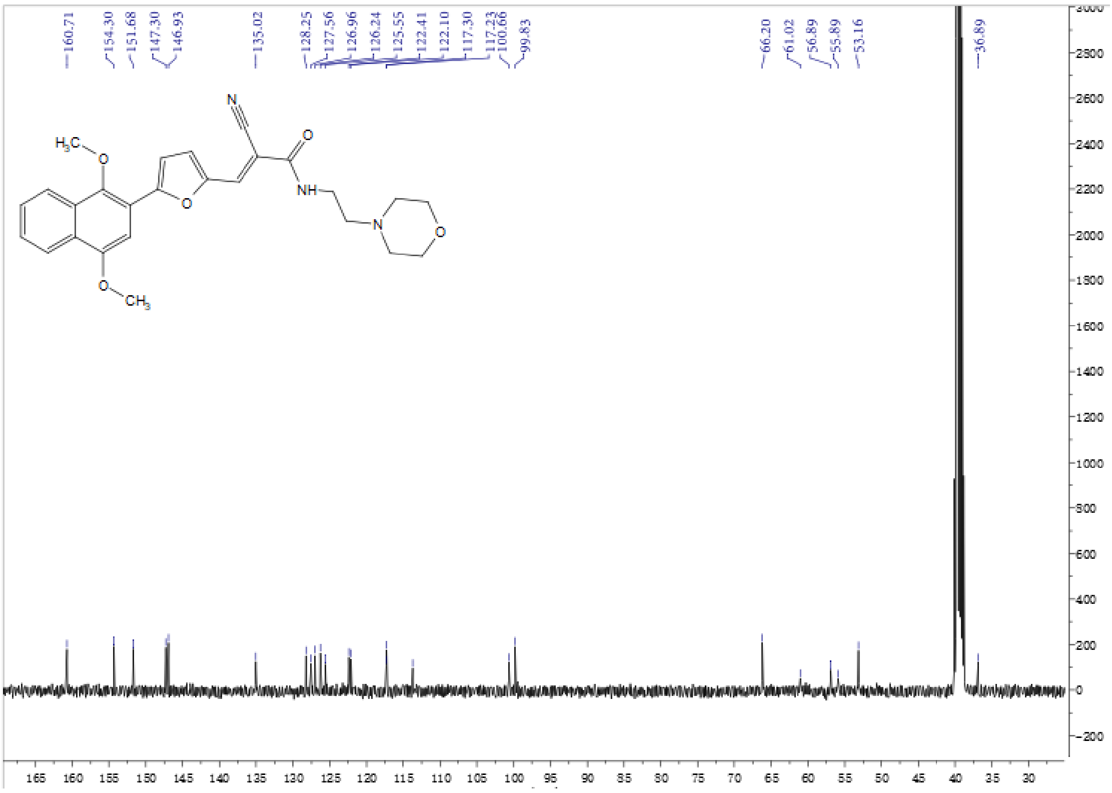

^{13}C-NMR of compound **4c**

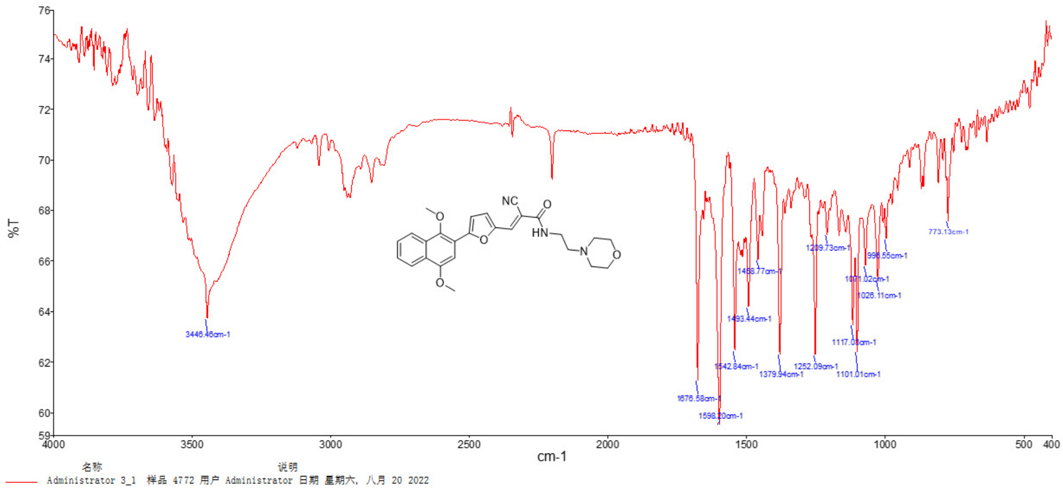

IR of compound **4c**

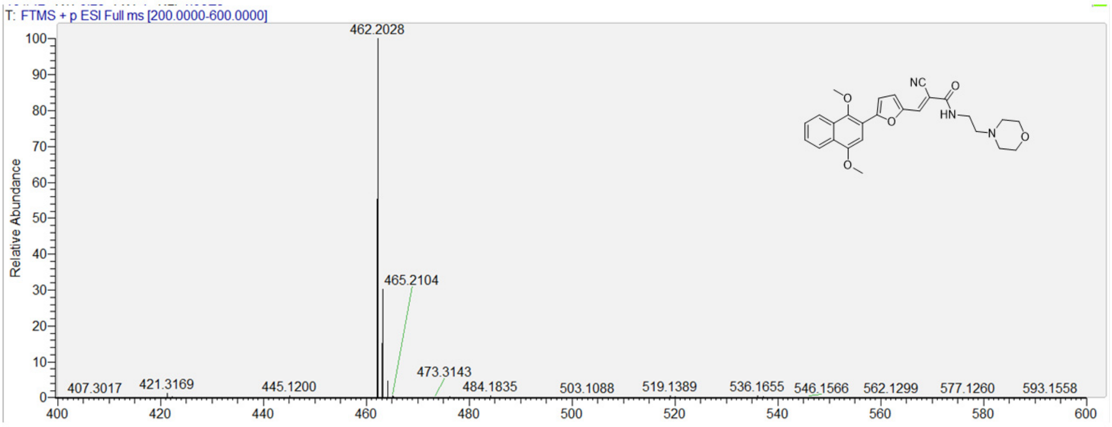

HRMS of compound **4c**

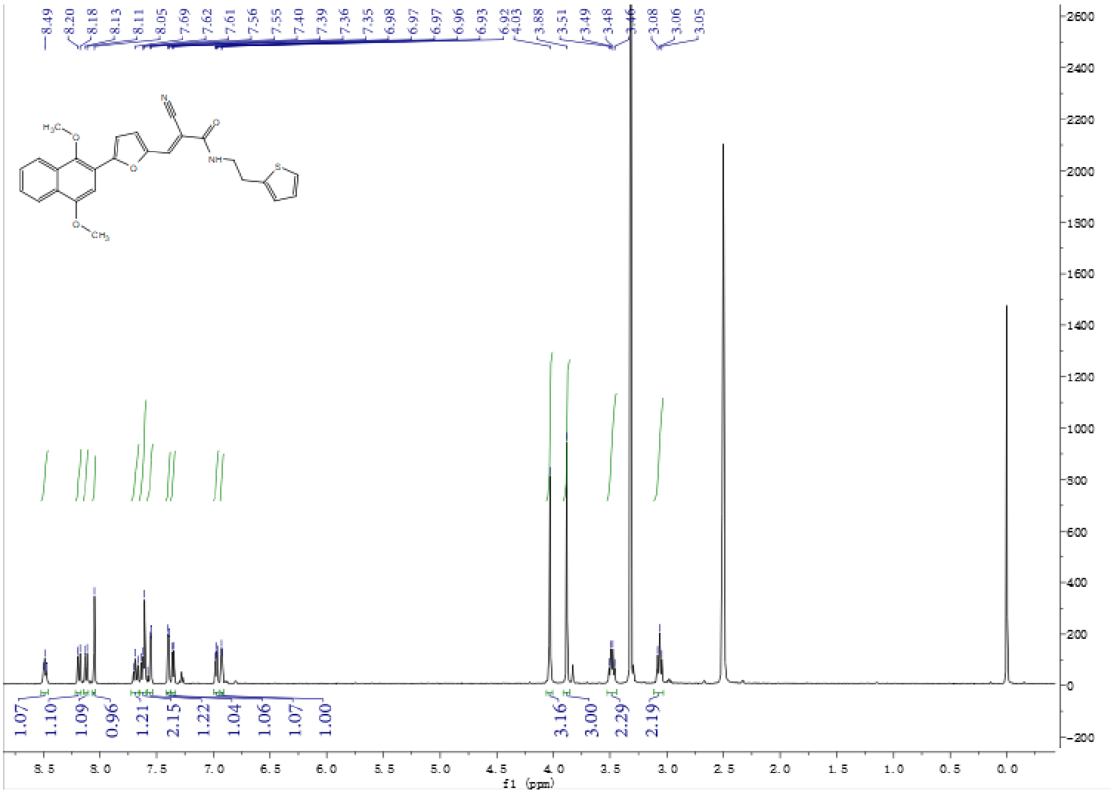

^{1}H-NMR of compound **4d**

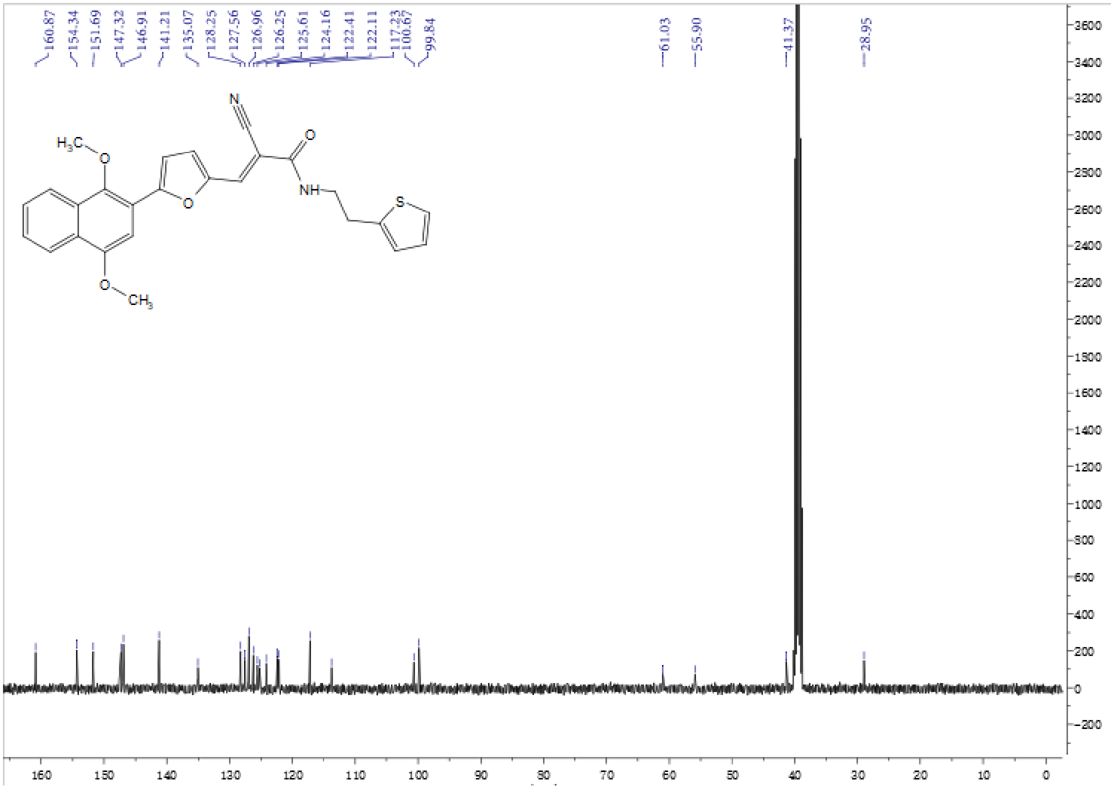

^{13}C-NMR of compound **4d**

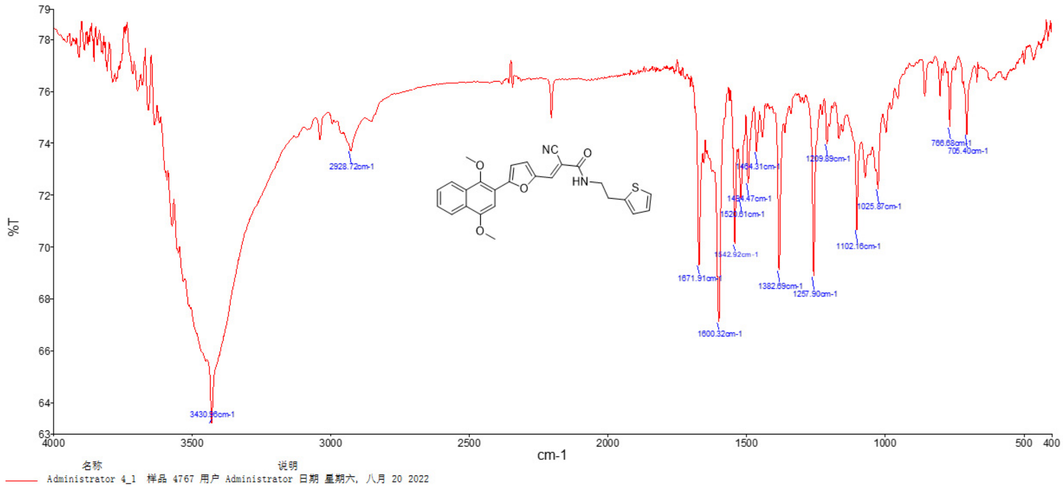

IR of compound **4d**

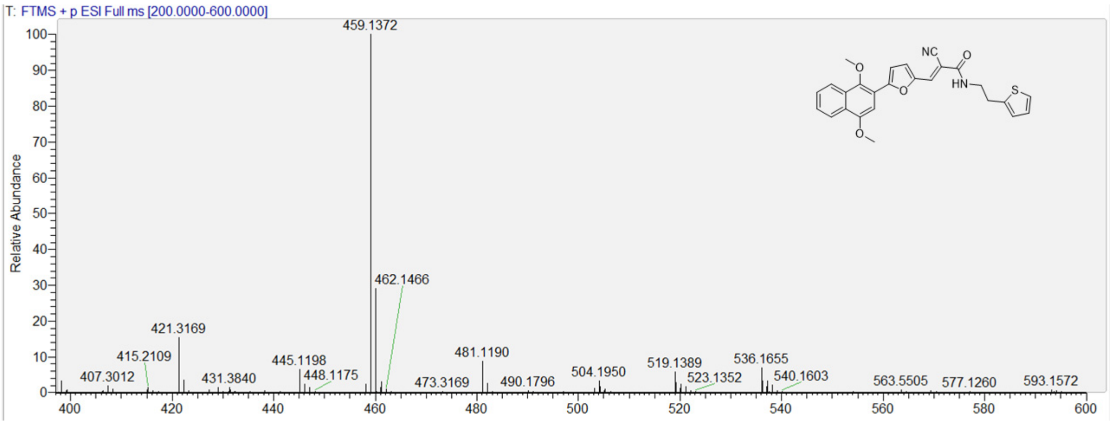

HRMS of compound **4d**

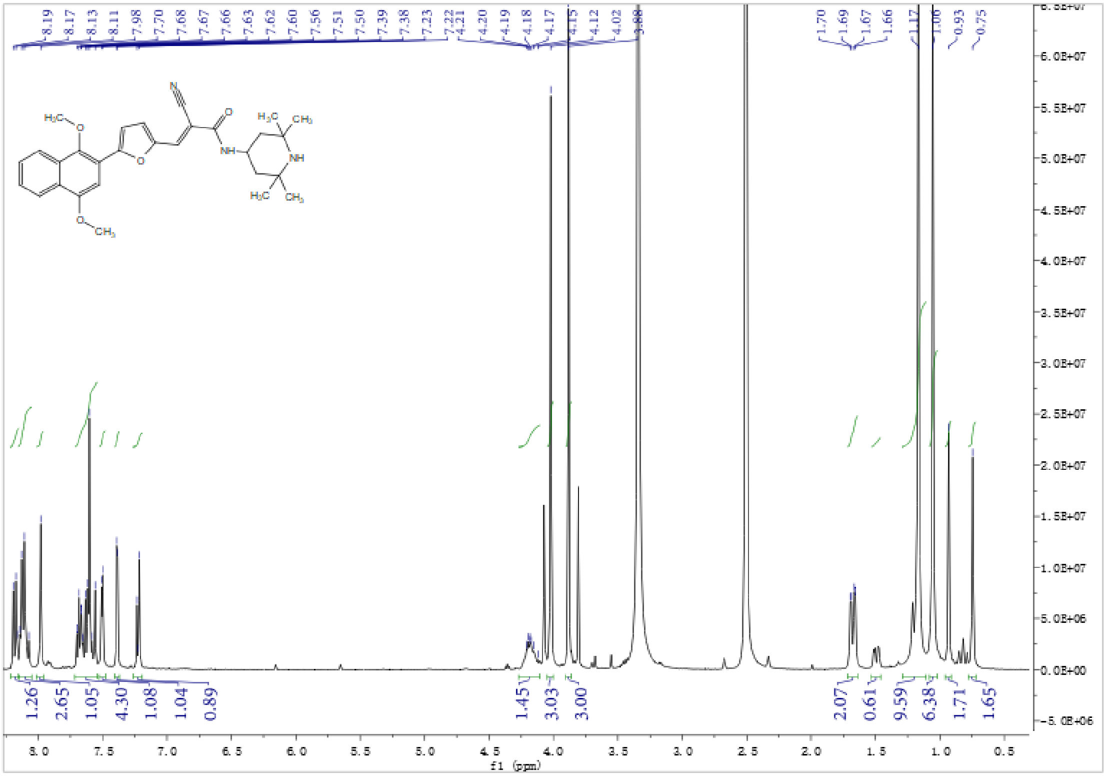

^{1}H-NMR of compound **4e**

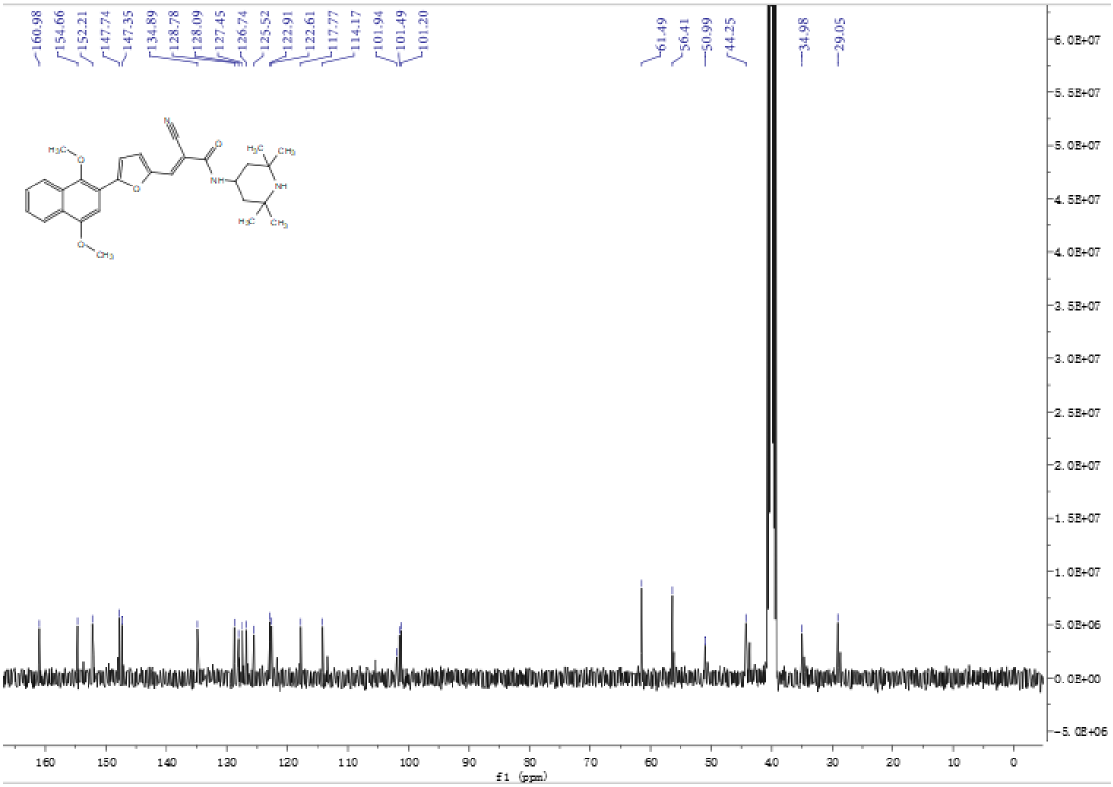

^{13}C-NMR of compound **4e**

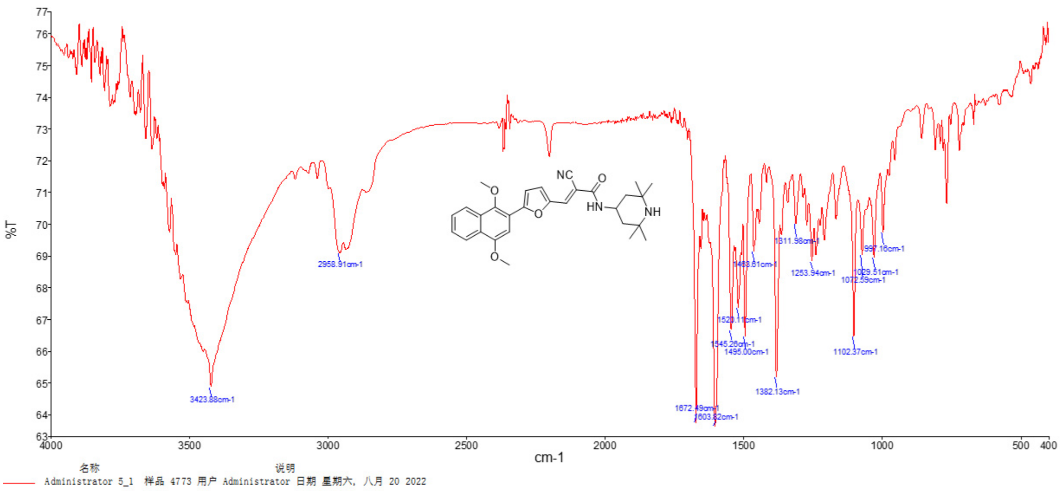

IR of compound **4e**

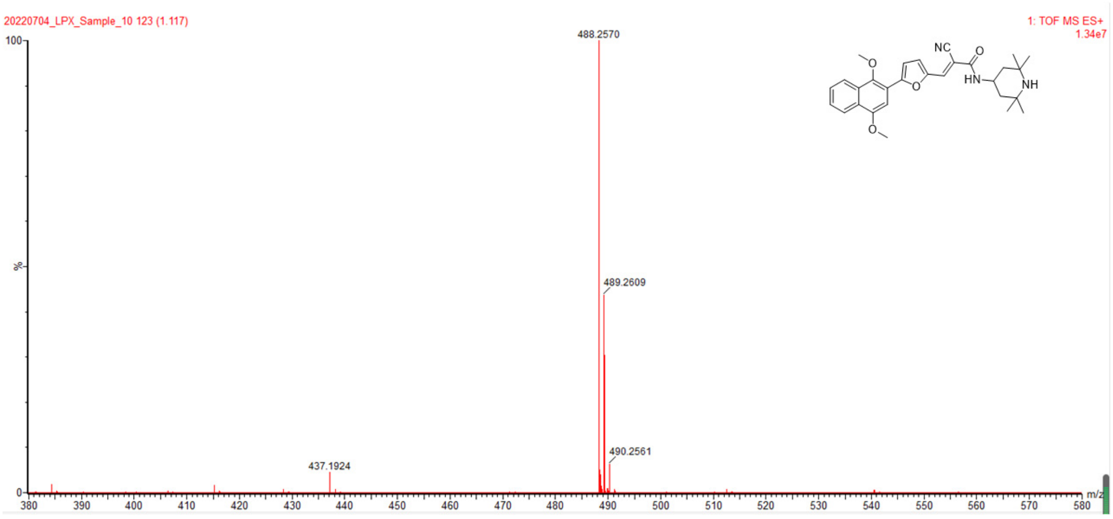

HRMS of compound **4e**

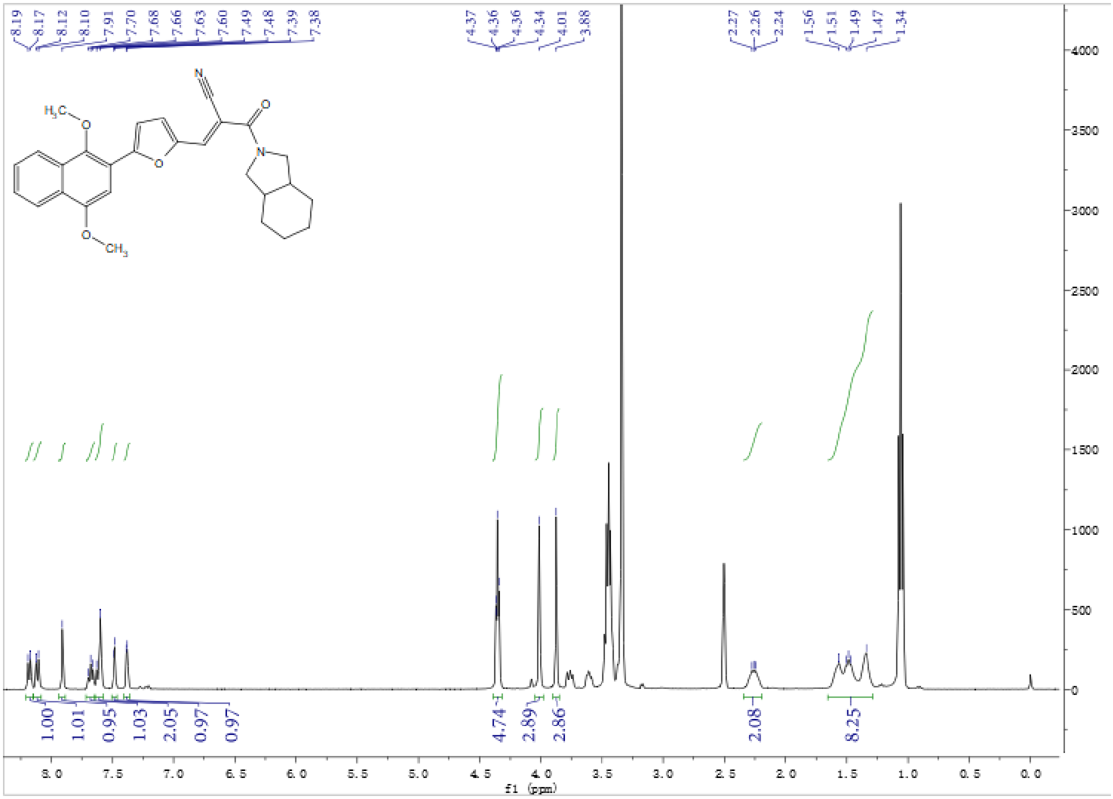

^{1}H-NMR of compound **4f**

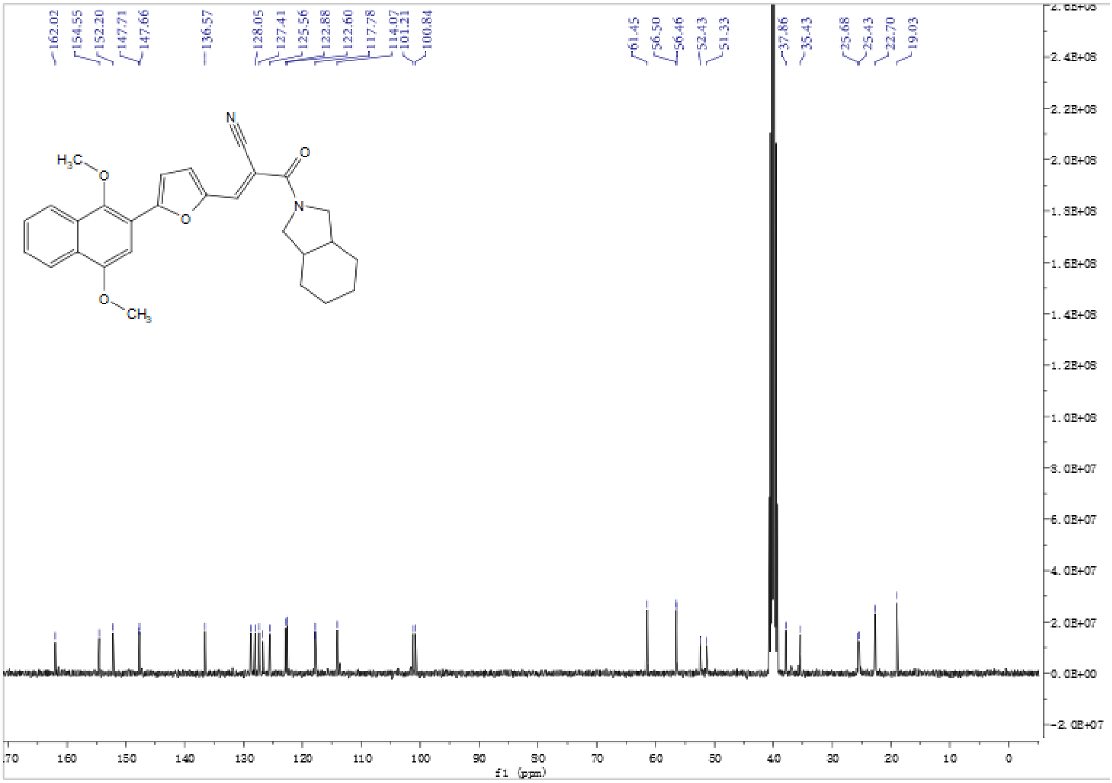

^{13}C-NMR of compound **4f**

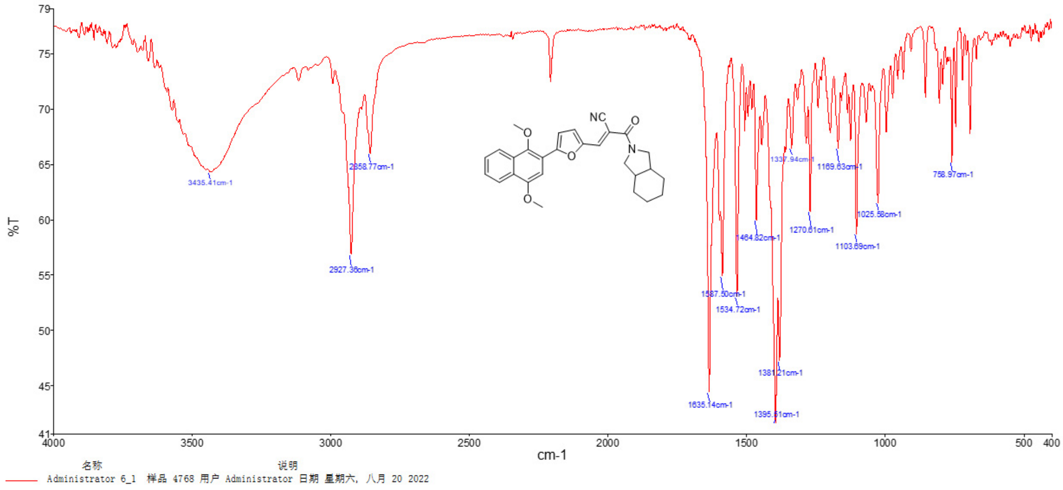

IR of compound **4f**

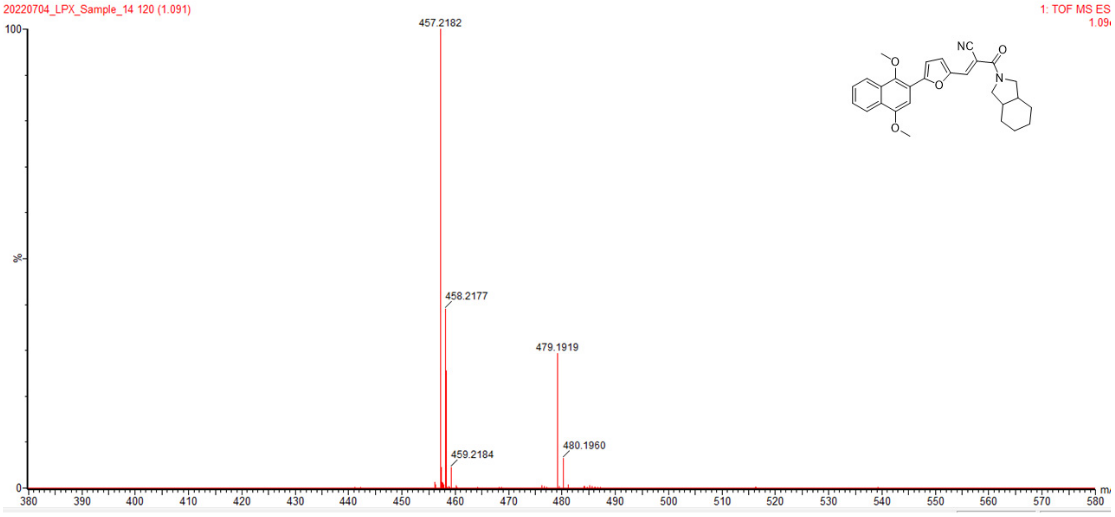

HRMS of compound **4f**

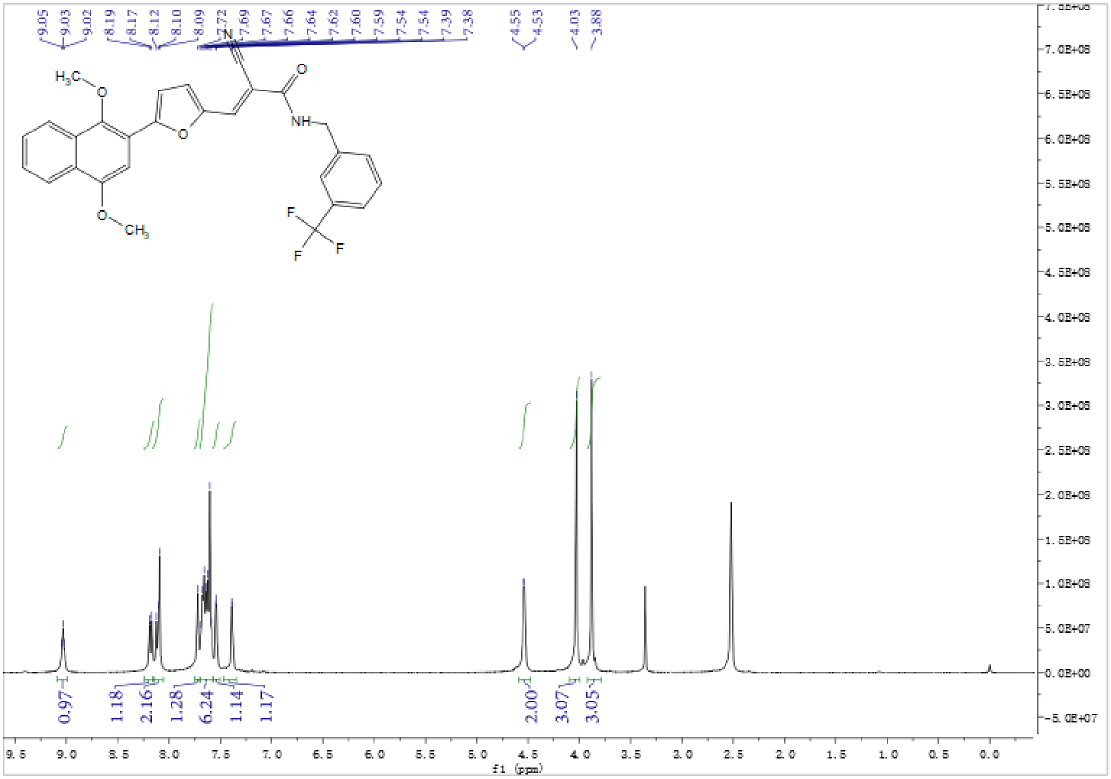

^{1}H-NMR of compound **4g**

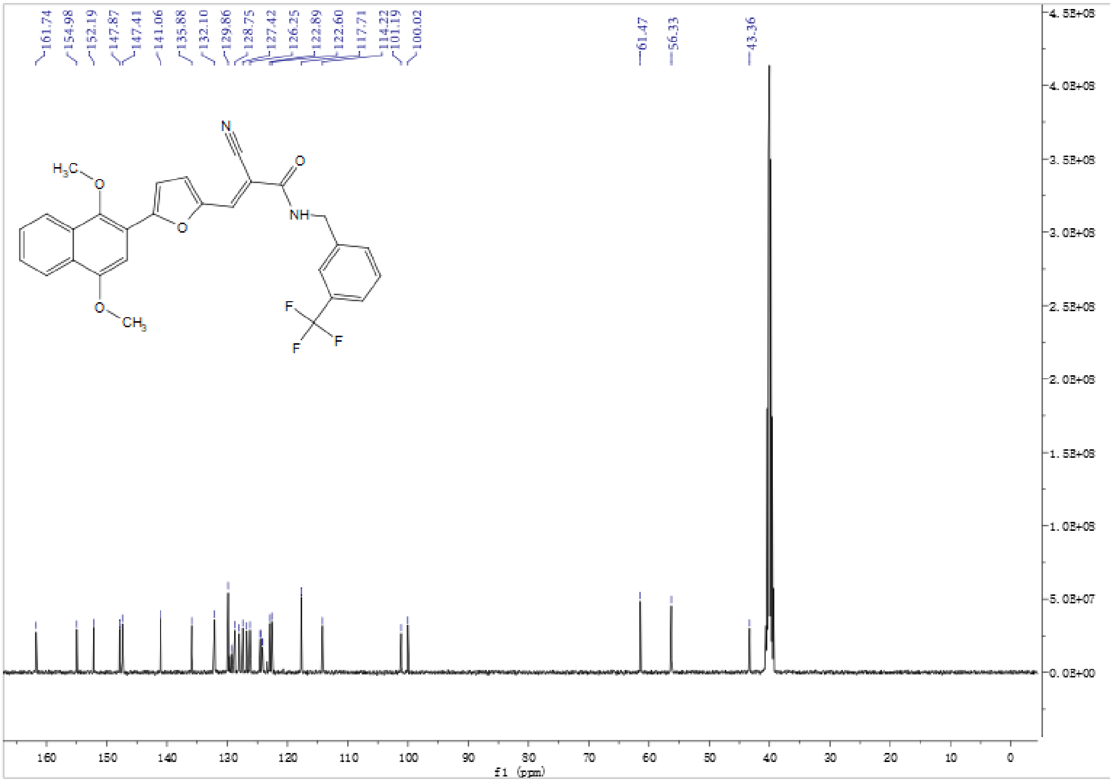

^{13}C-NMR of compound **4g**

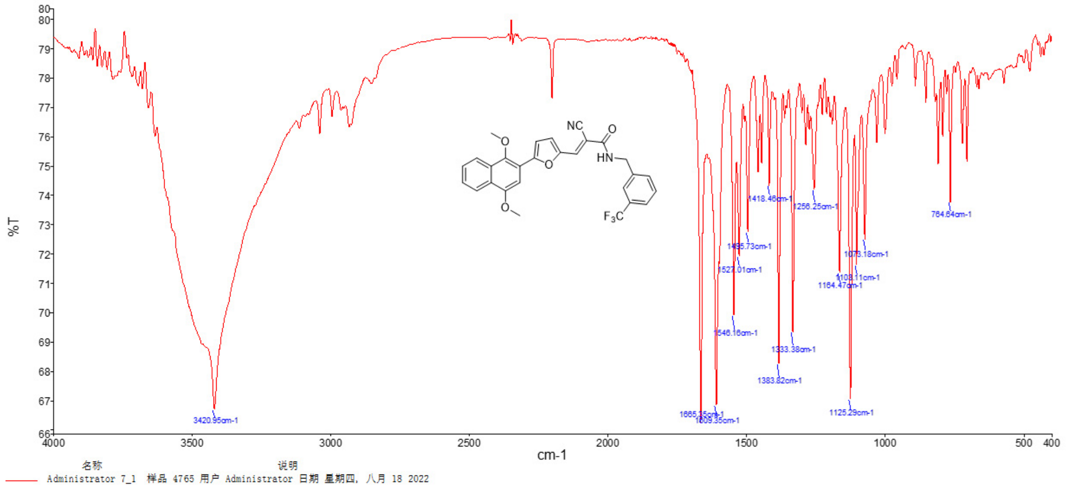

IR of compound **4g**

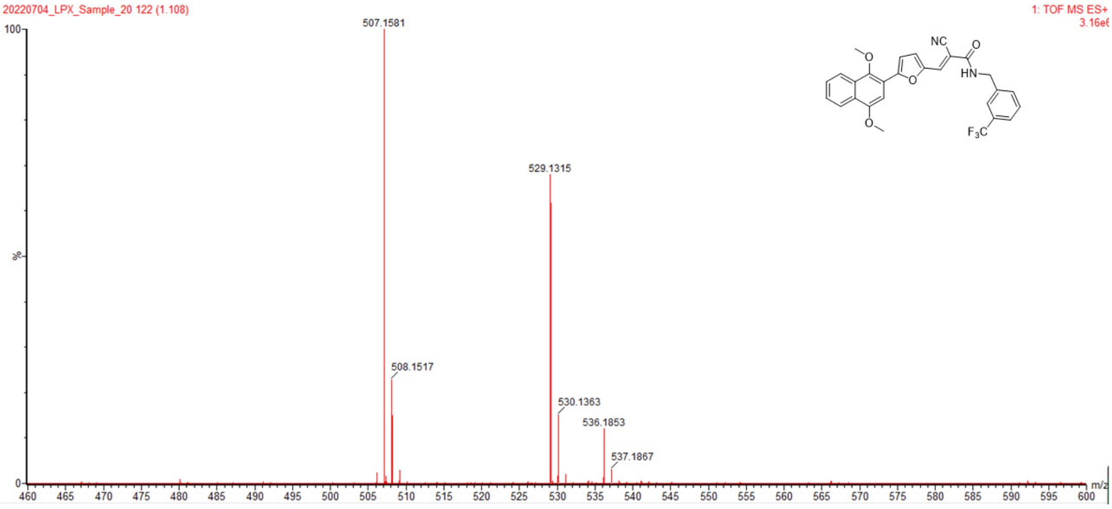

HRMS of compound **4g**

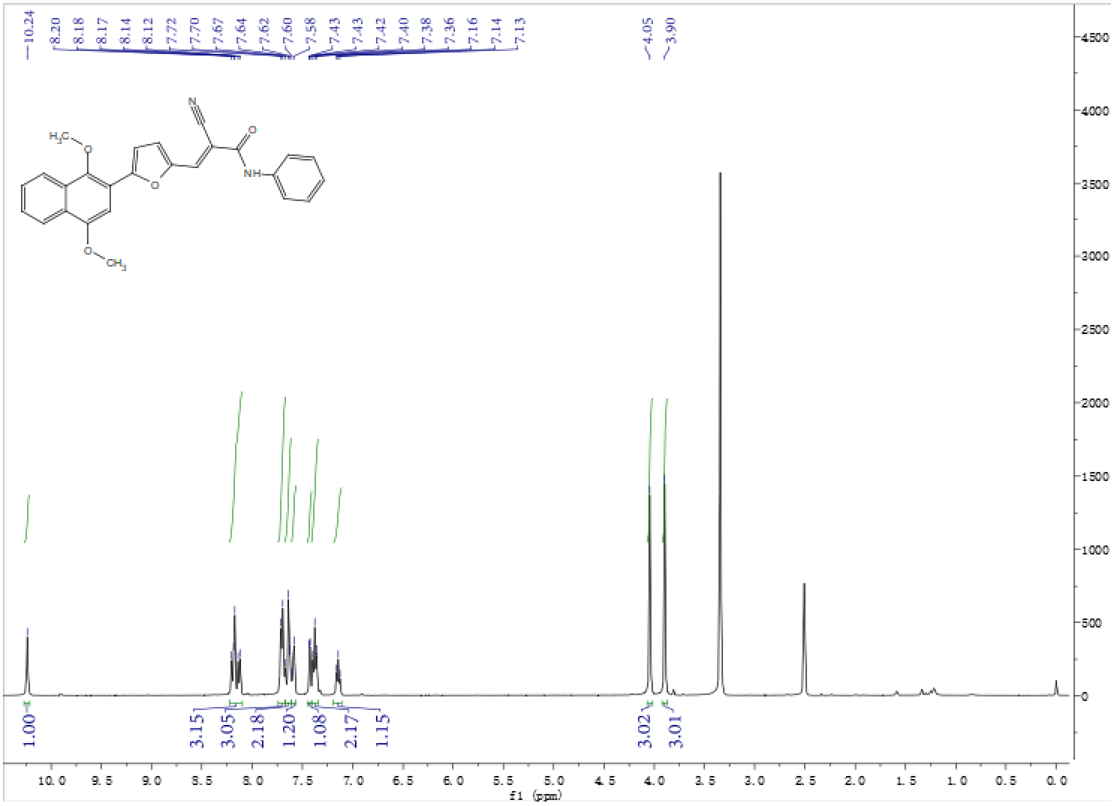

^{1}H-NMR of compound **4h**

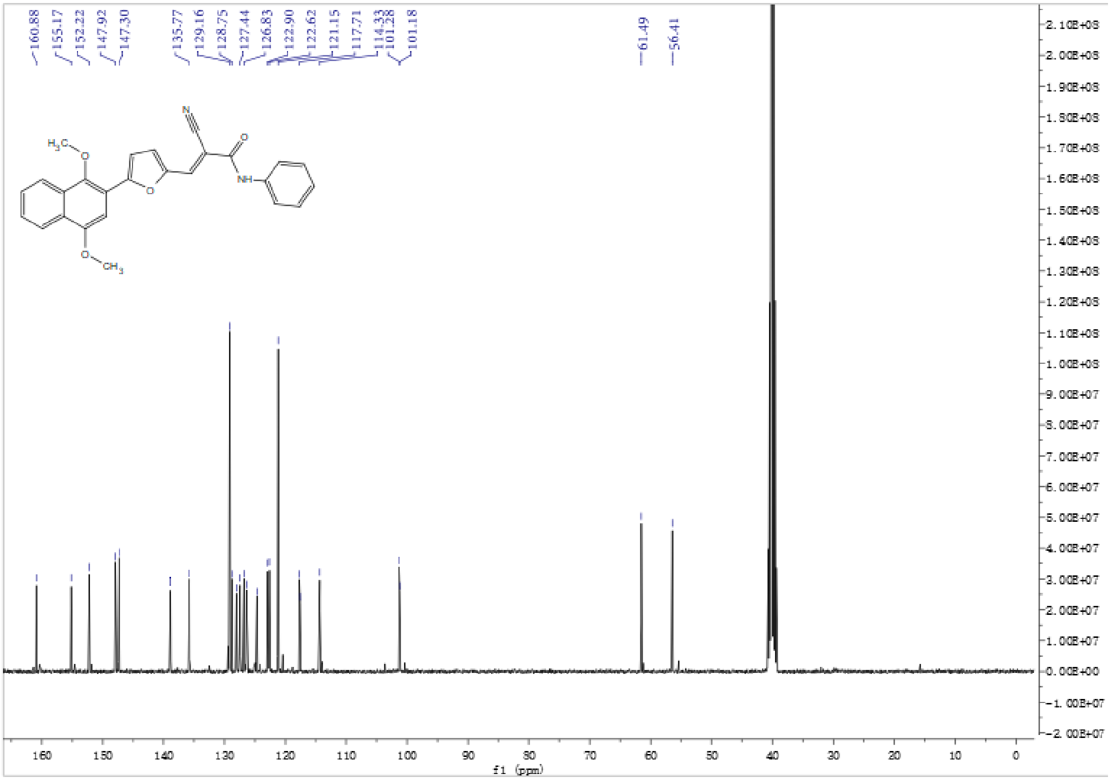

^{13}C-NMR of compound **4h**

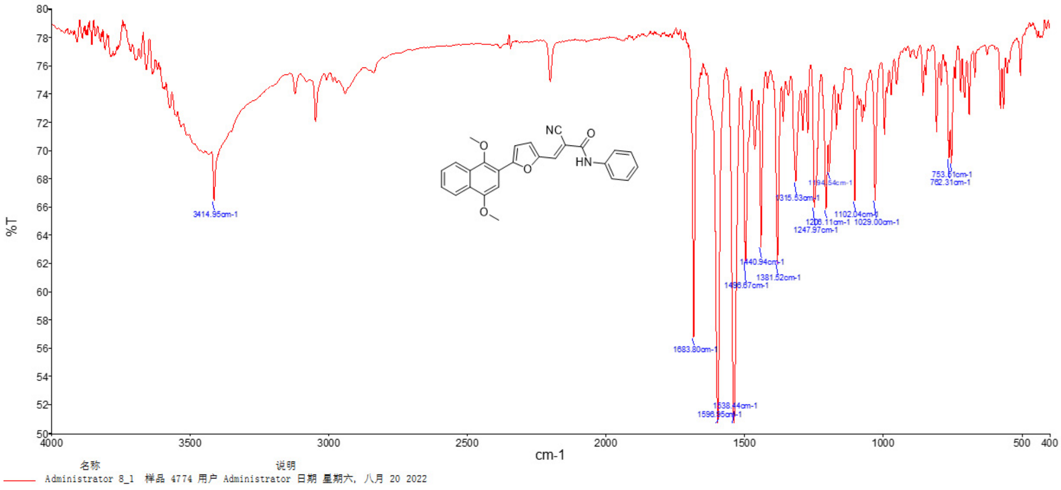

IR of compound **4h**

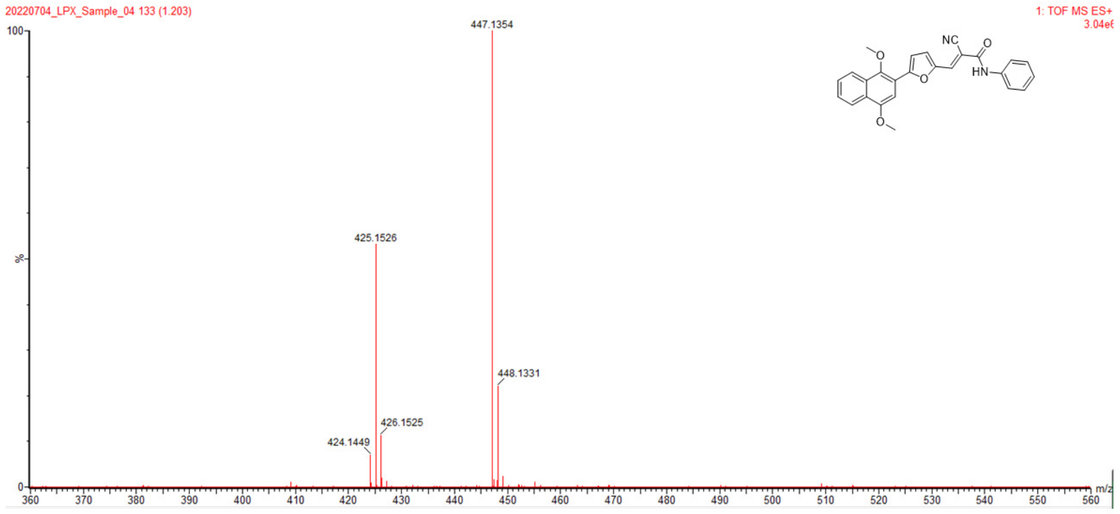

HRMS of compound **4h**

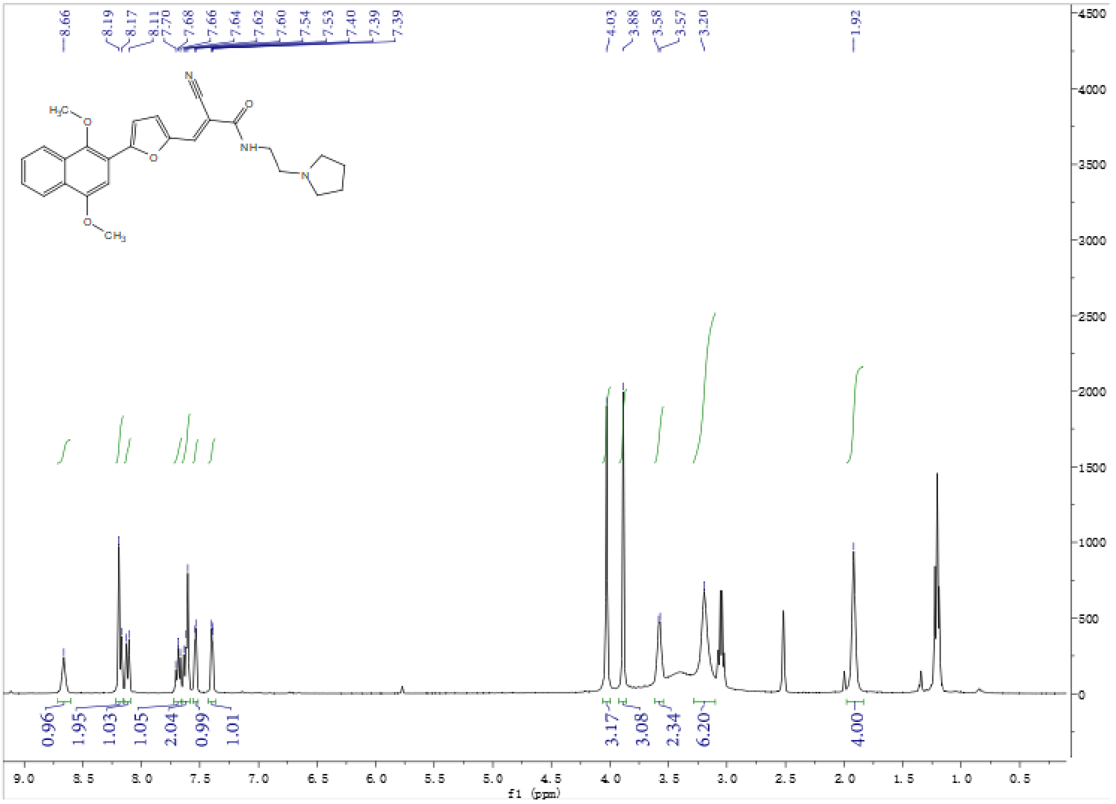

^{1}H-NMR of compound **4i**

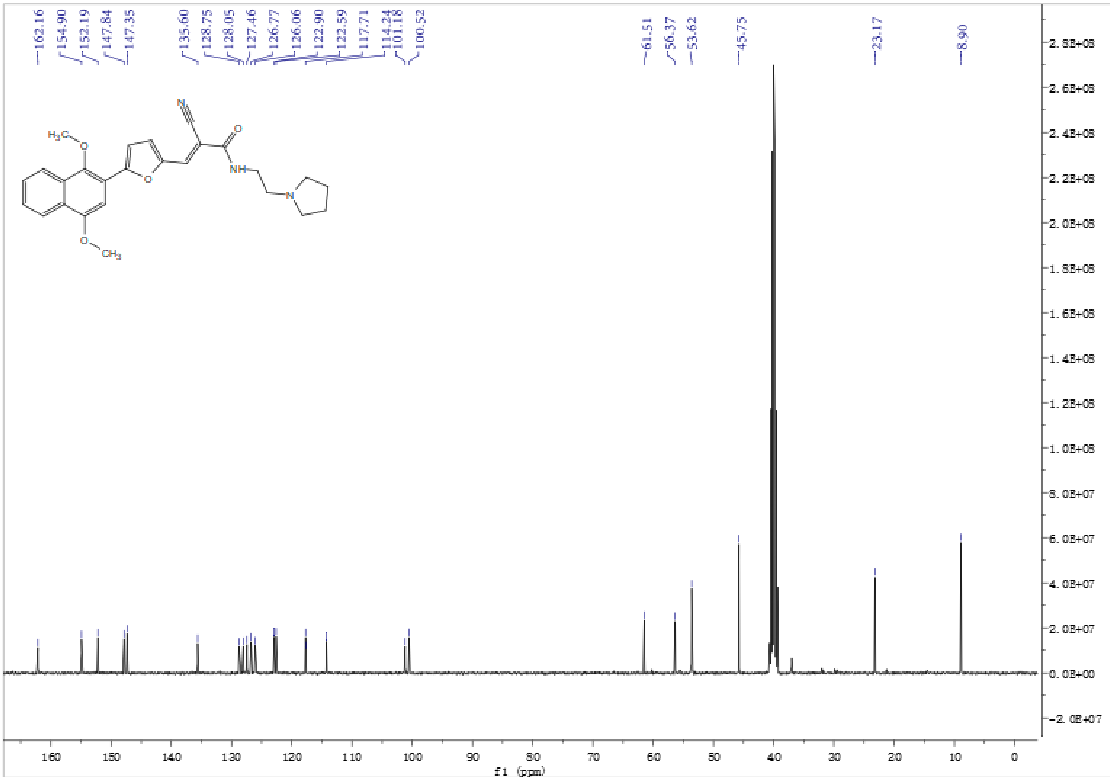

^{13}C-NMR of compound **4i**

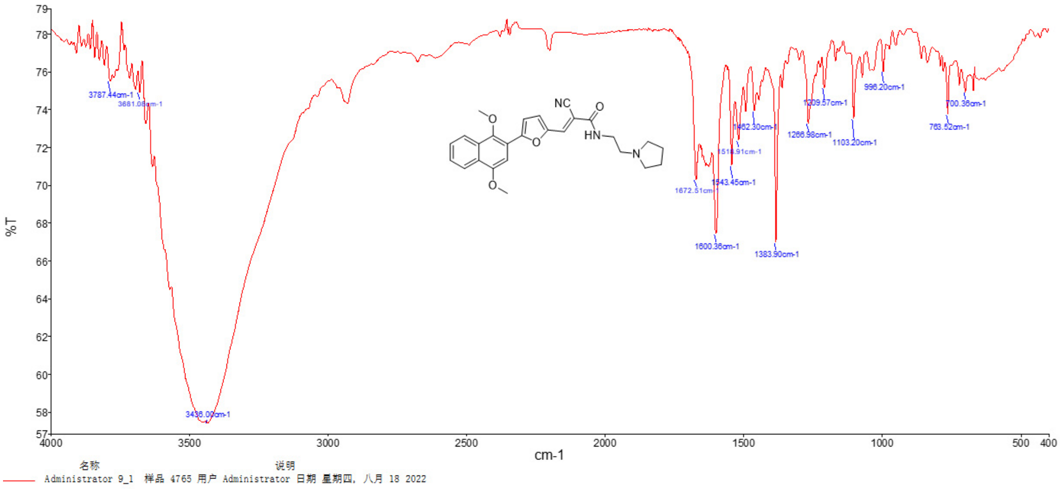

IR of compound **4i**

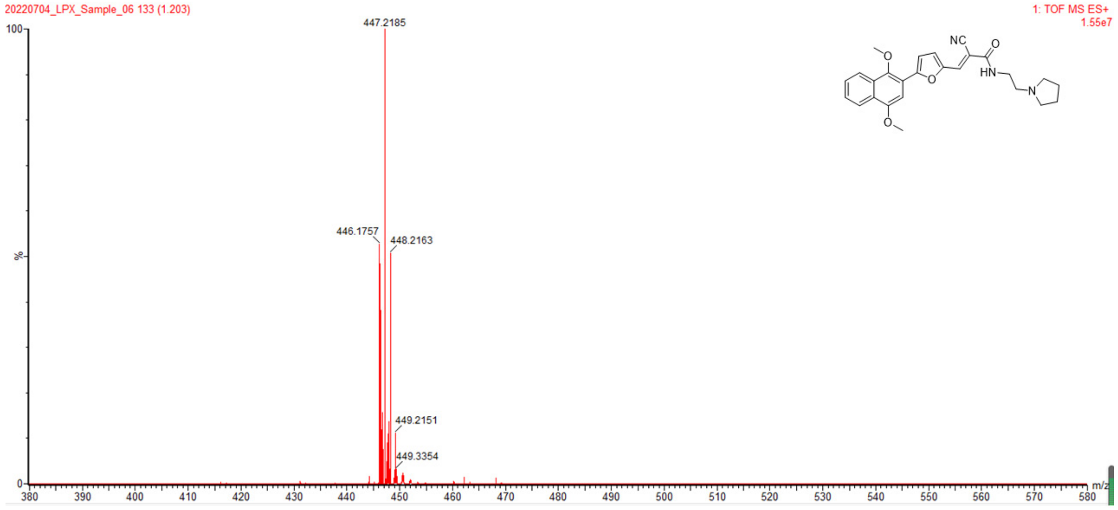

HRMS of compound **4i**

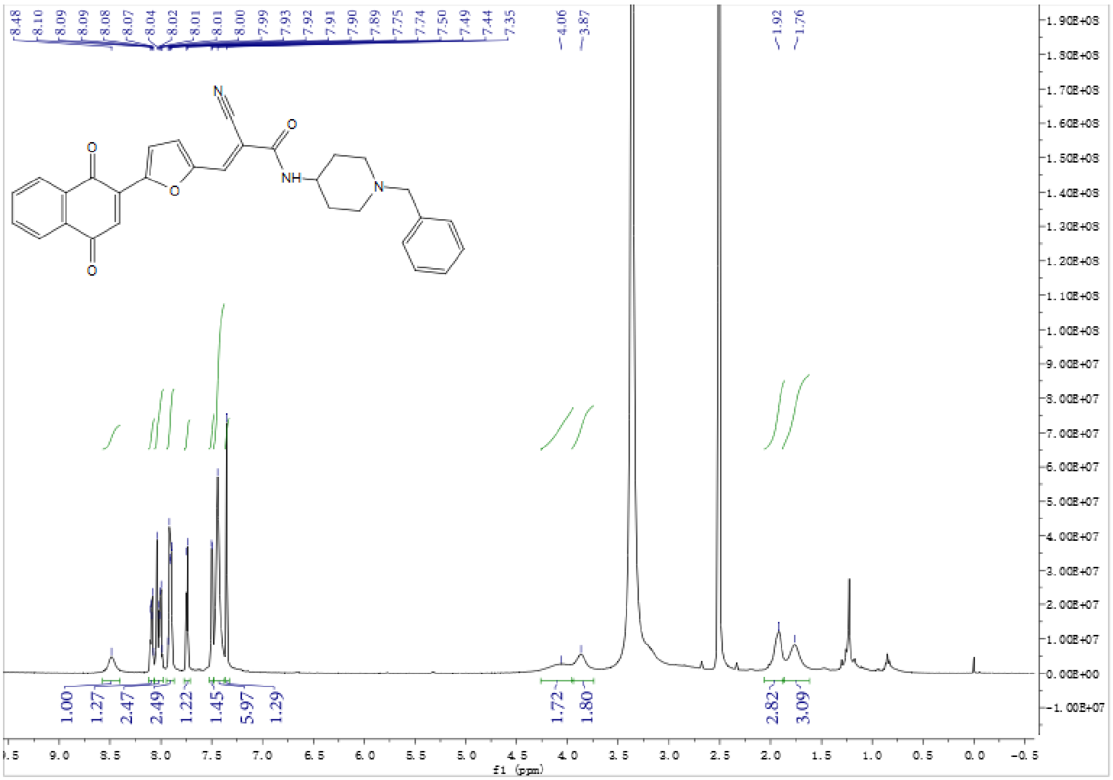

^{1}H-NMR of compound **5a**

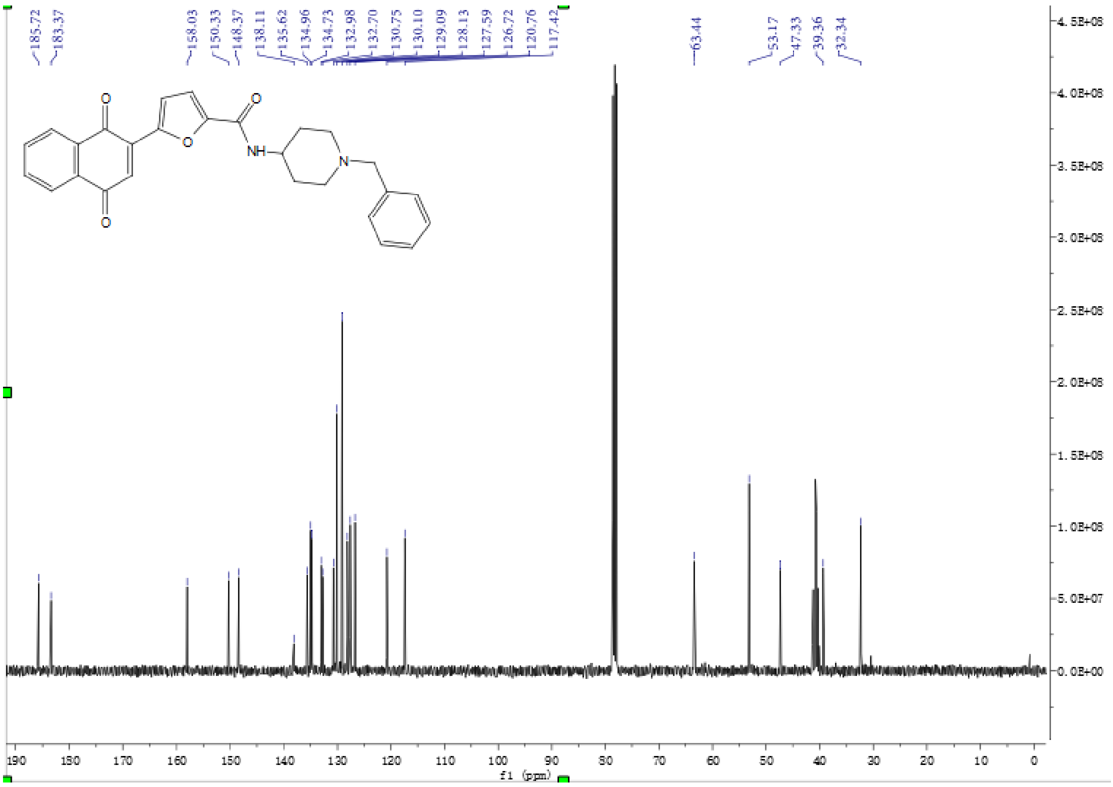

^{13}C-NMR of compound **5a**

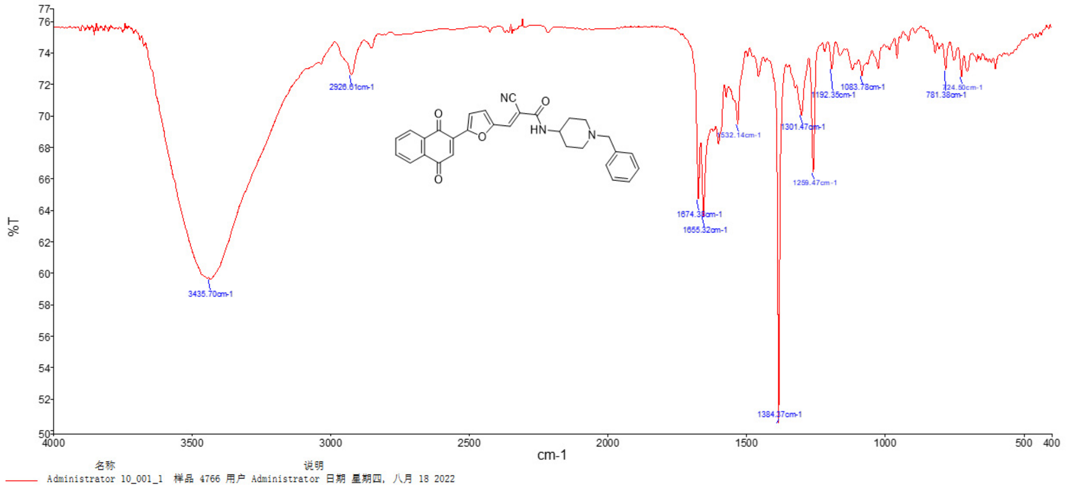

IR of compound **5a**

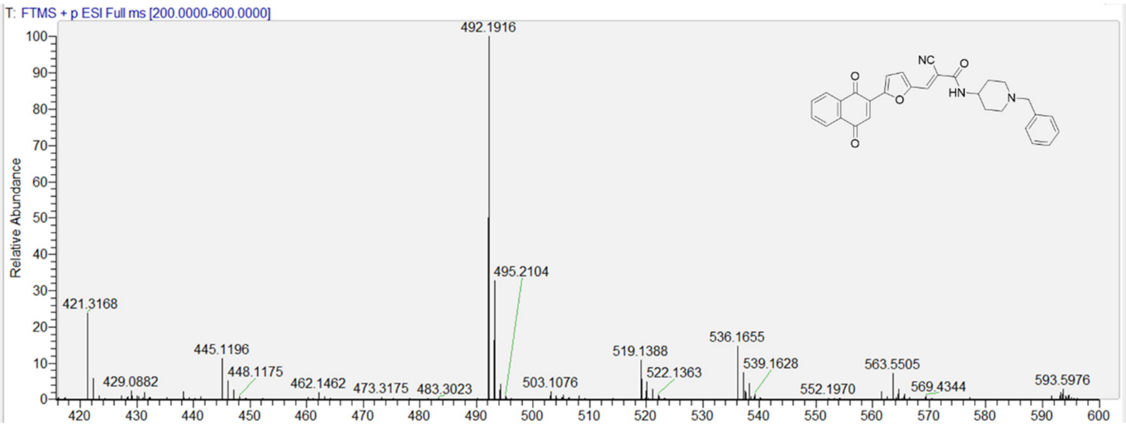

HRMS of compound **5a**

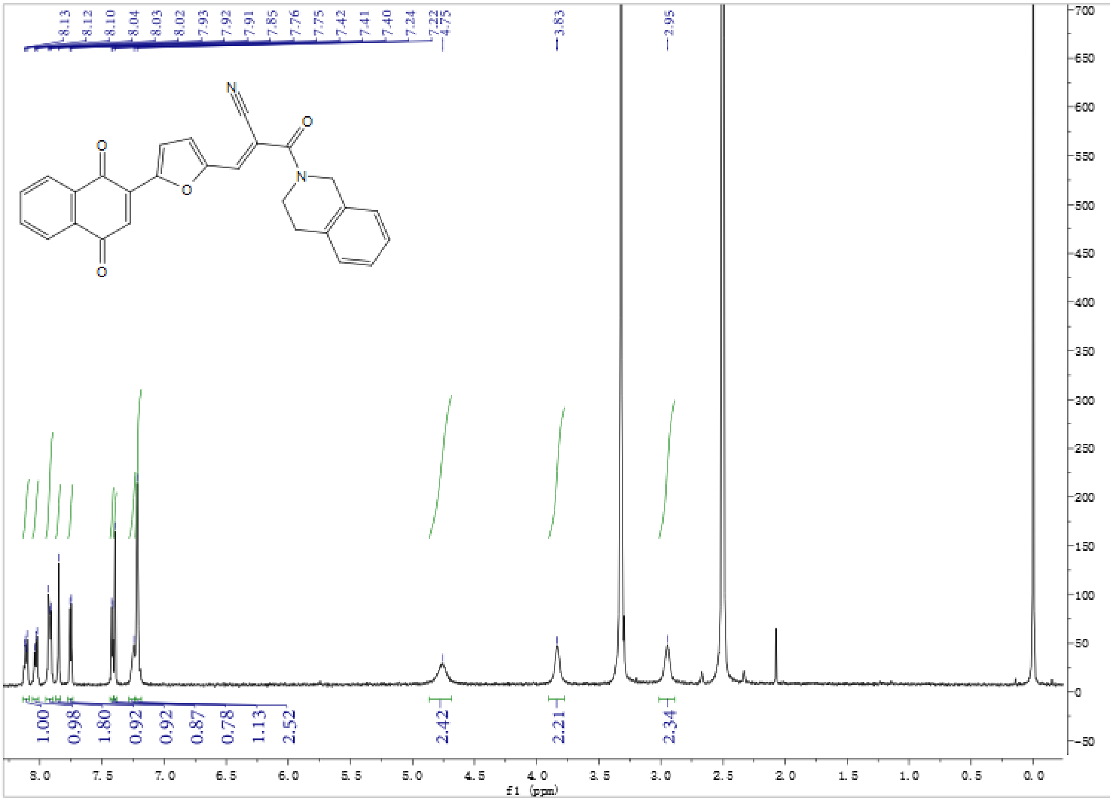

^{1}H-NMR of compound **5b**

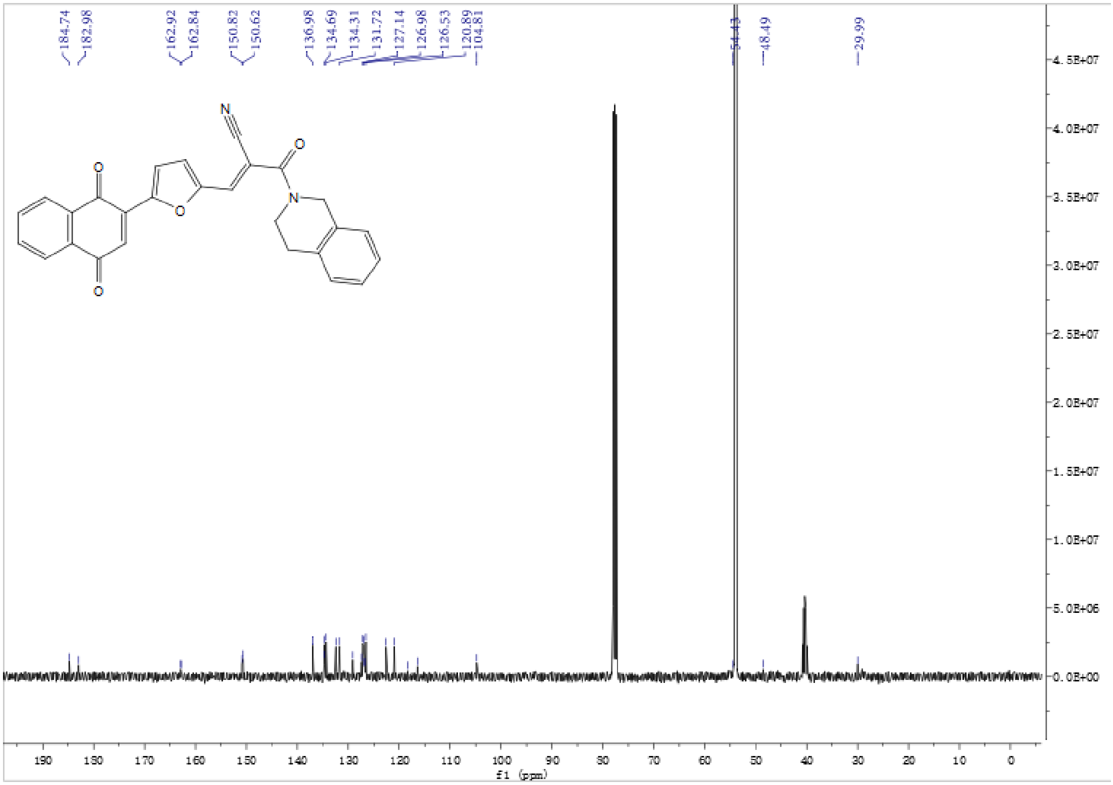

^{13}C-NMR of compound **5b**

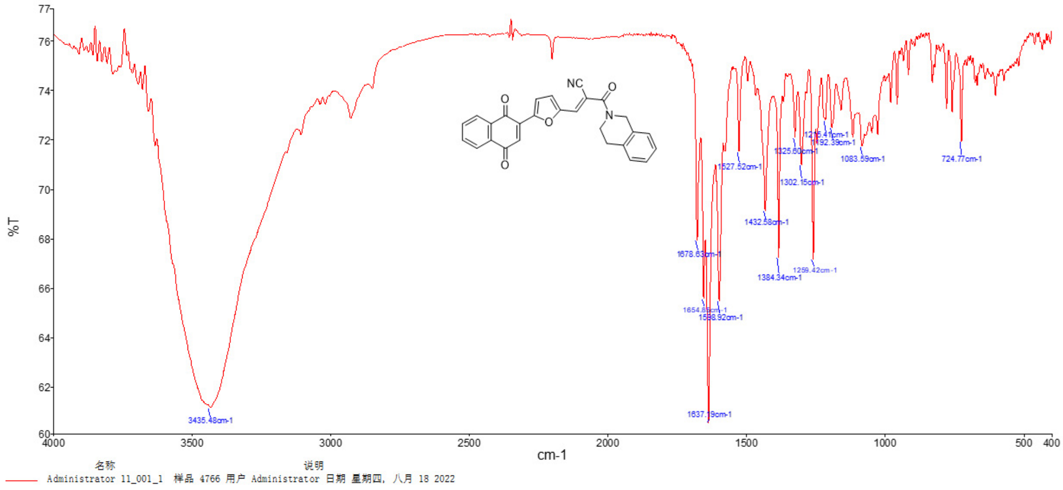

IR of compound **5b**

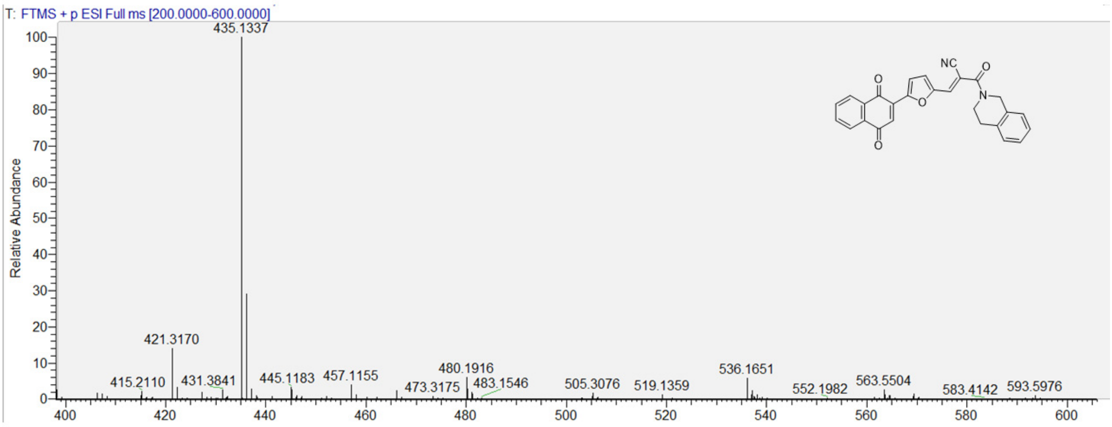

HRMS of compound **5b**

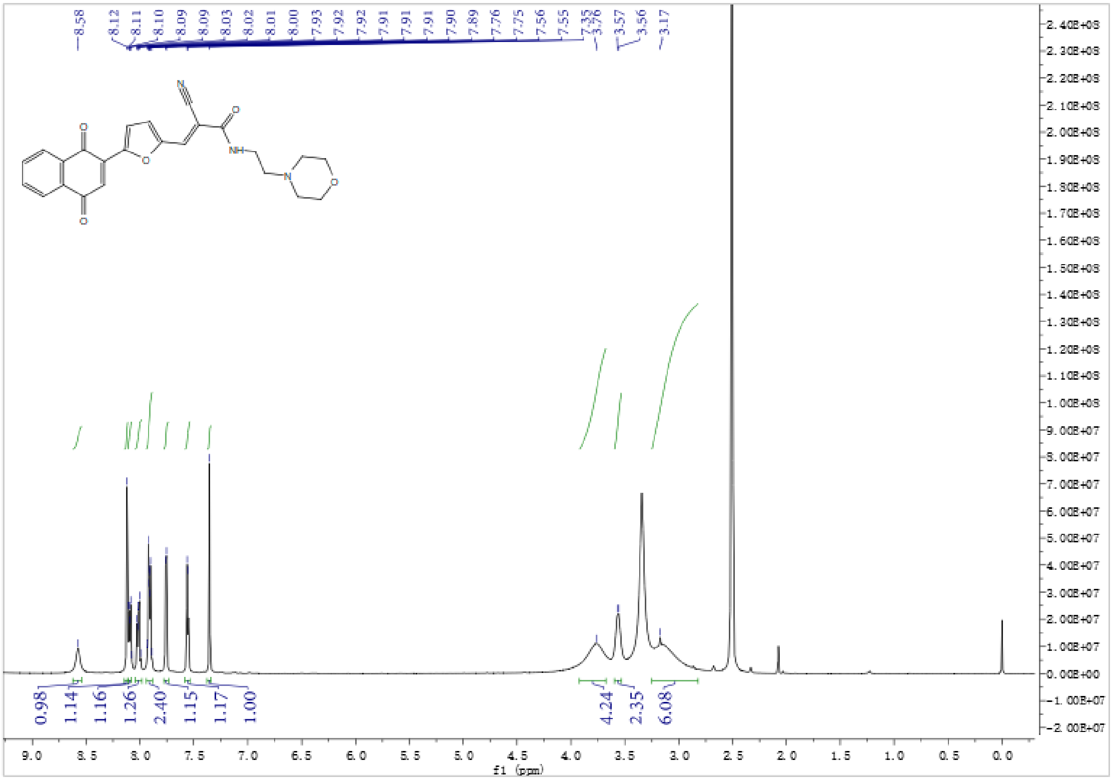

^{1}H-NMR of compound **5c**

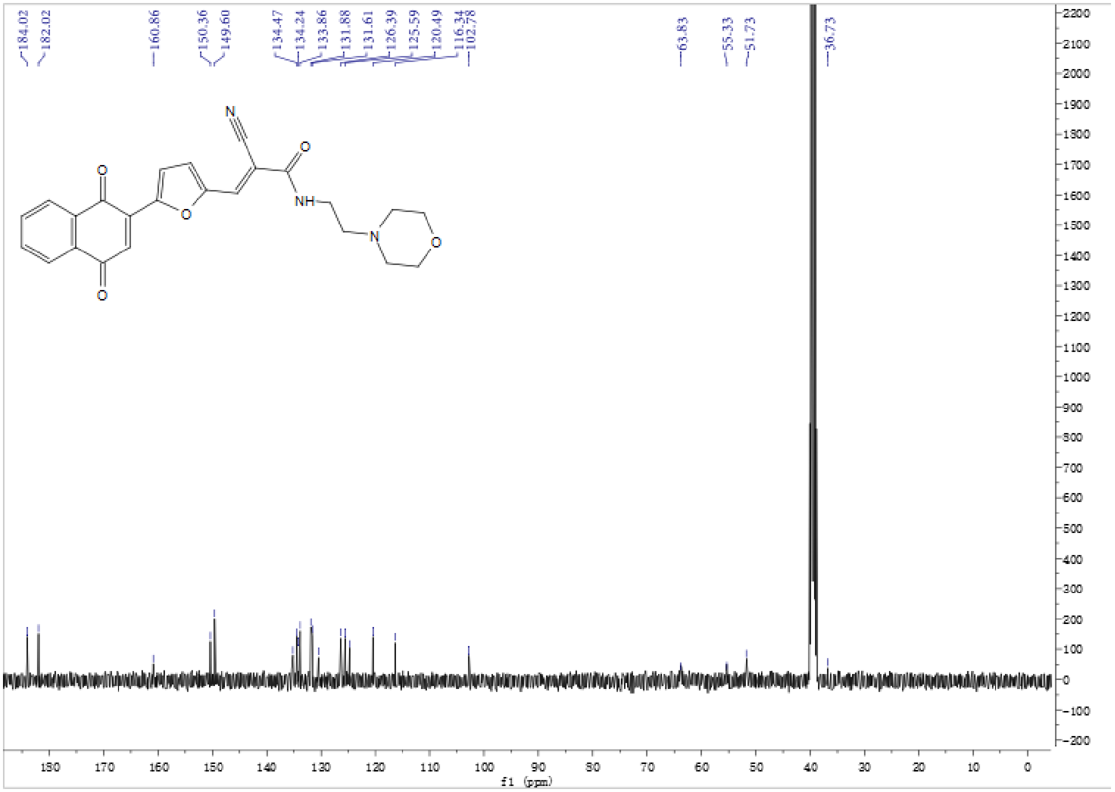

^{13}C-NMR of compound **5c**

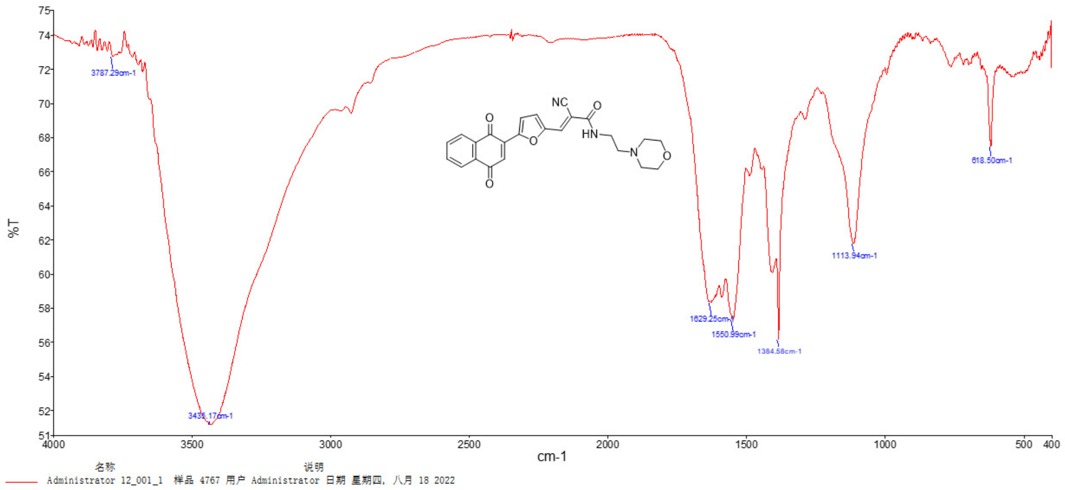

IR of compound **5c**

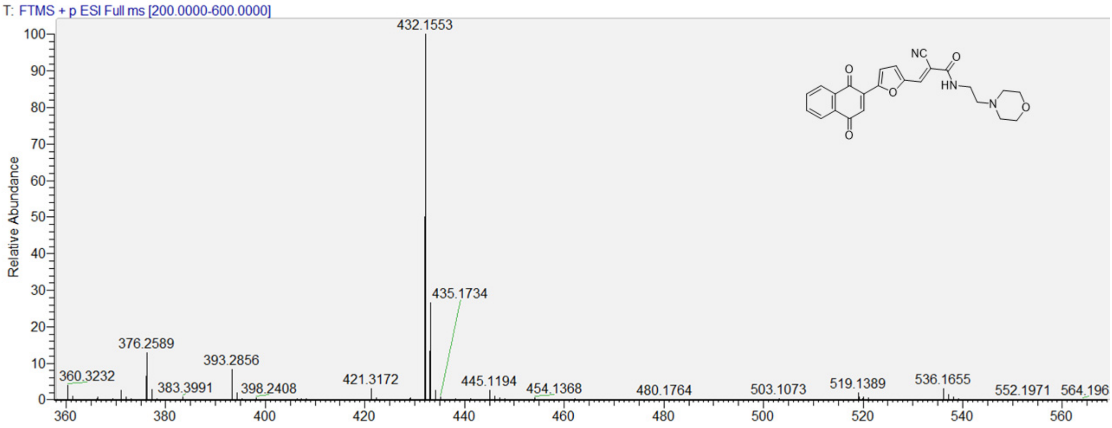

HRMS of compound **5c**

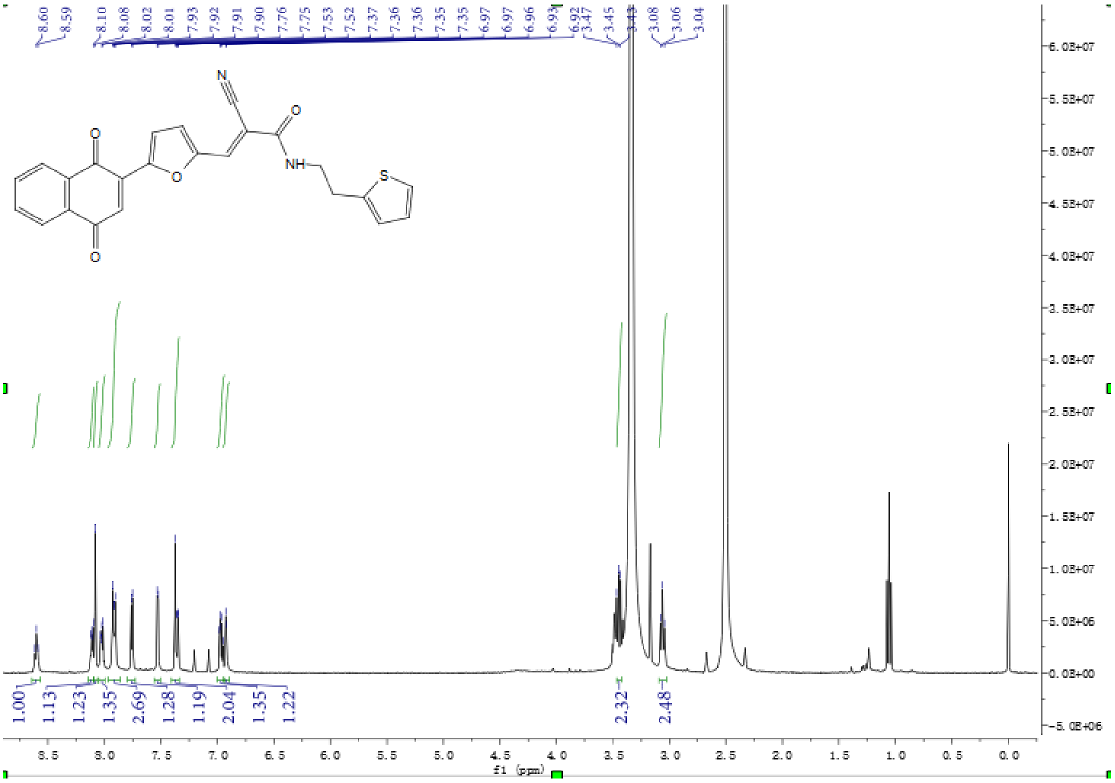

^{1}H-NMR of compound **5d**

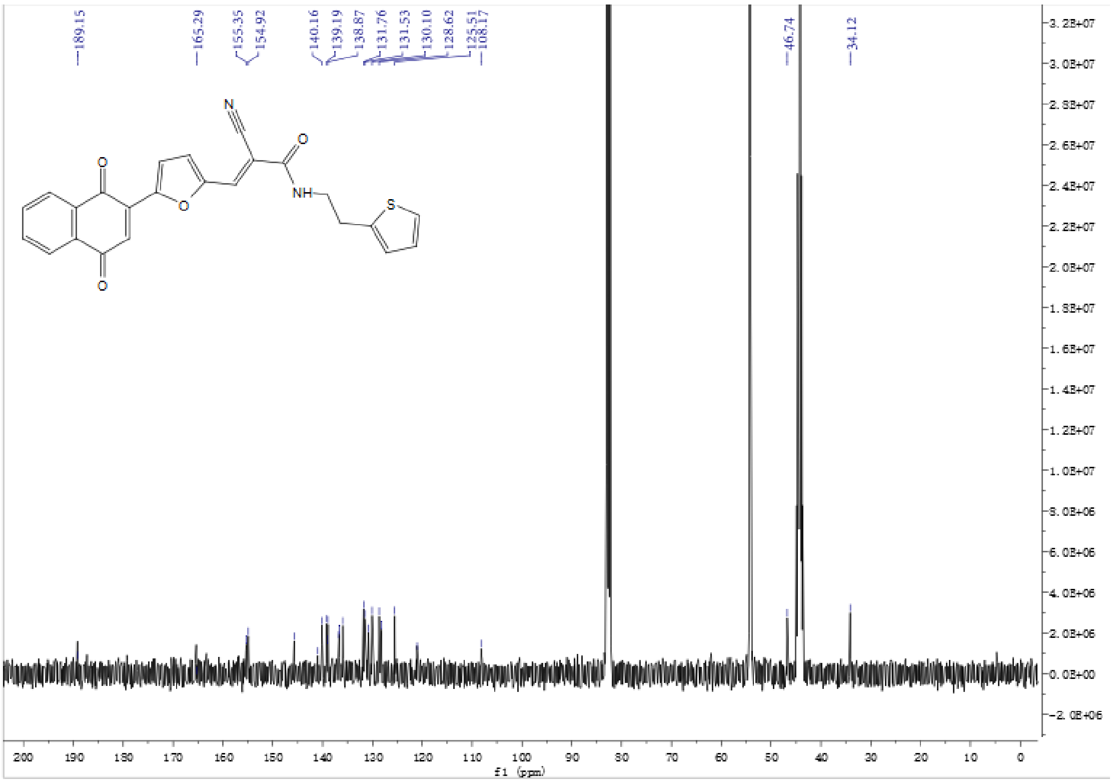

^{13}C-NMR of compound **5d**

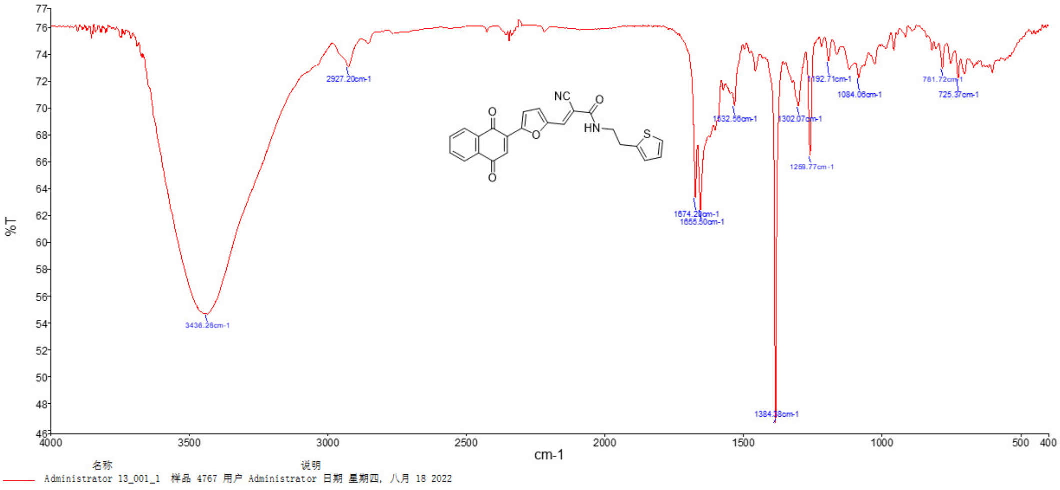

IR of compound **5d**

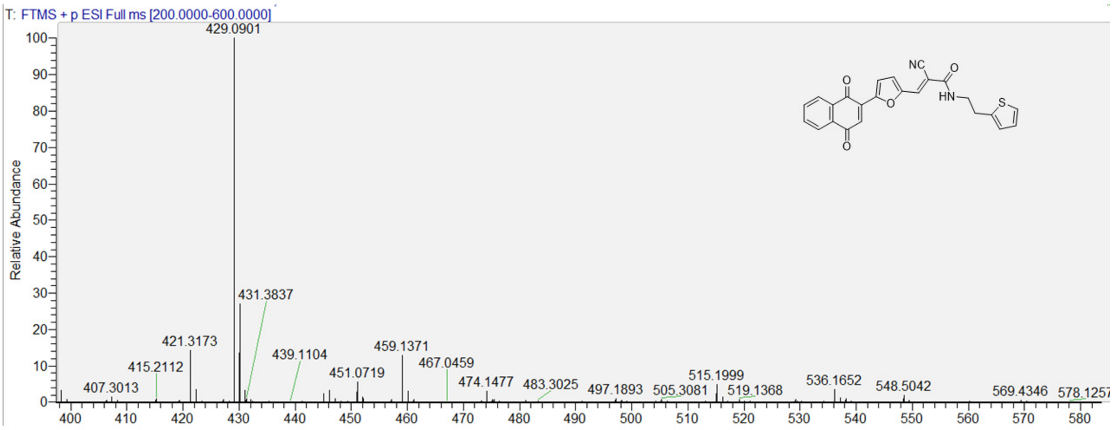

HRMS of compound **5d**

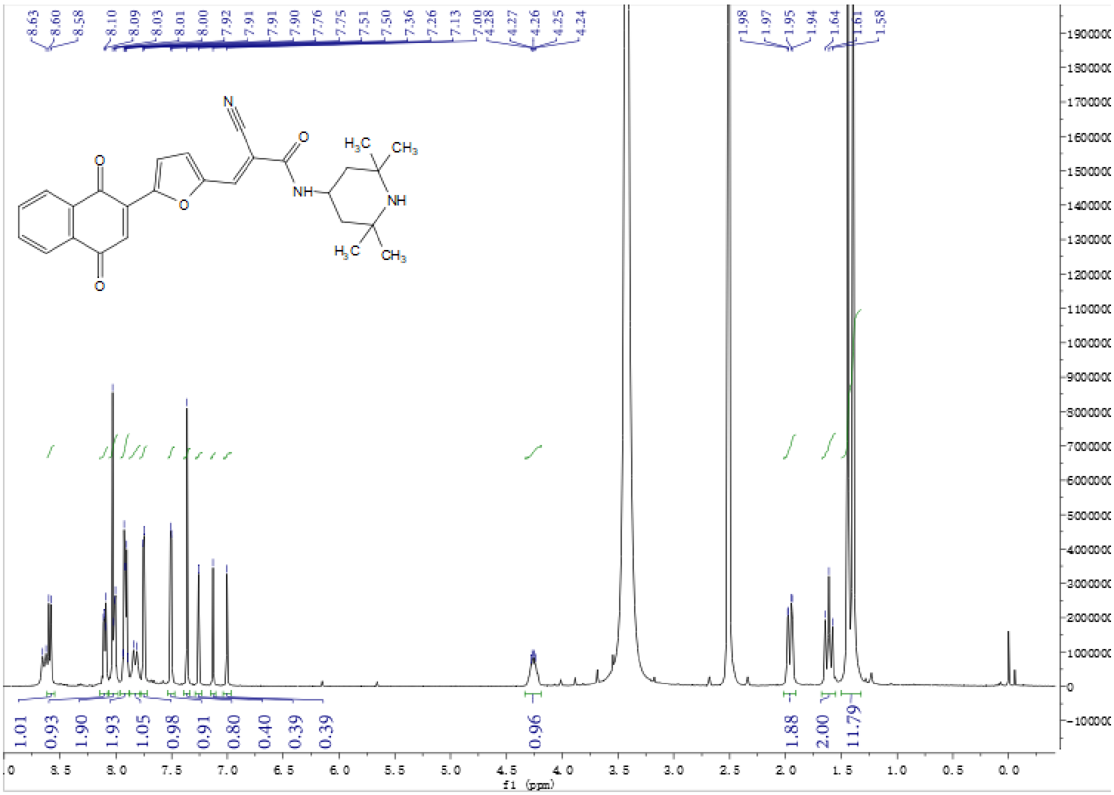

^{1}H-NMR of compound **5e**

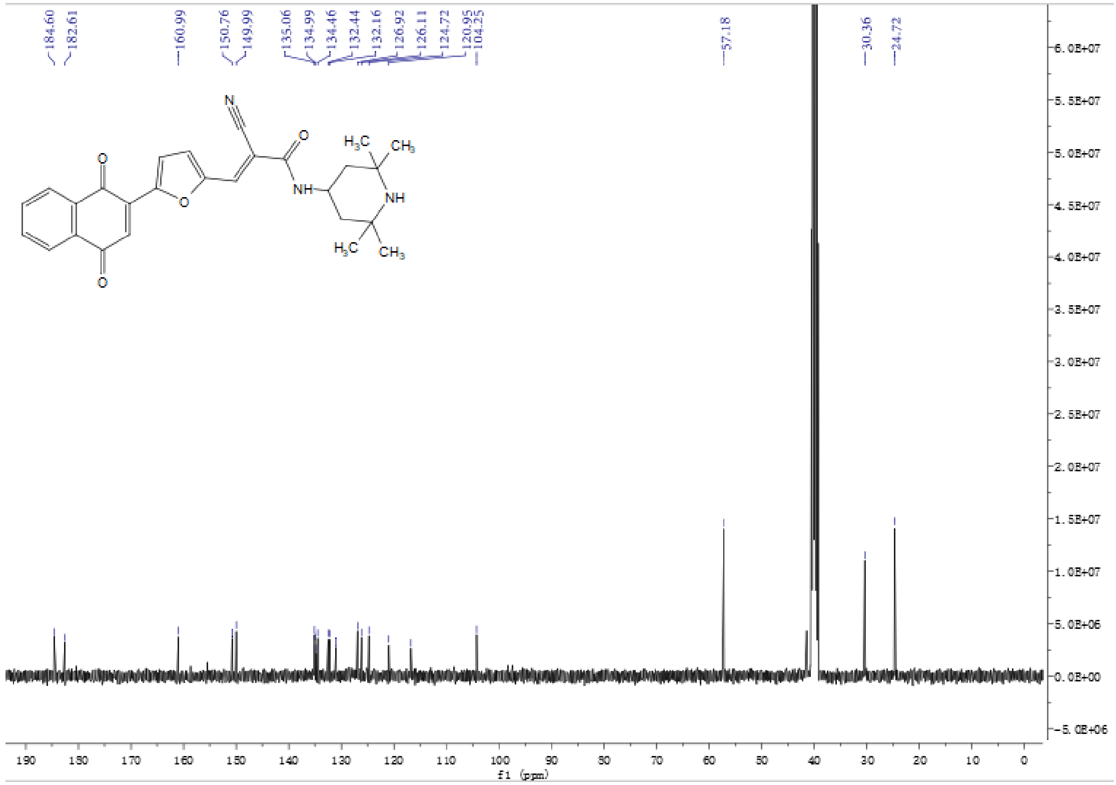

^{13}C-NMR of compound **5e**

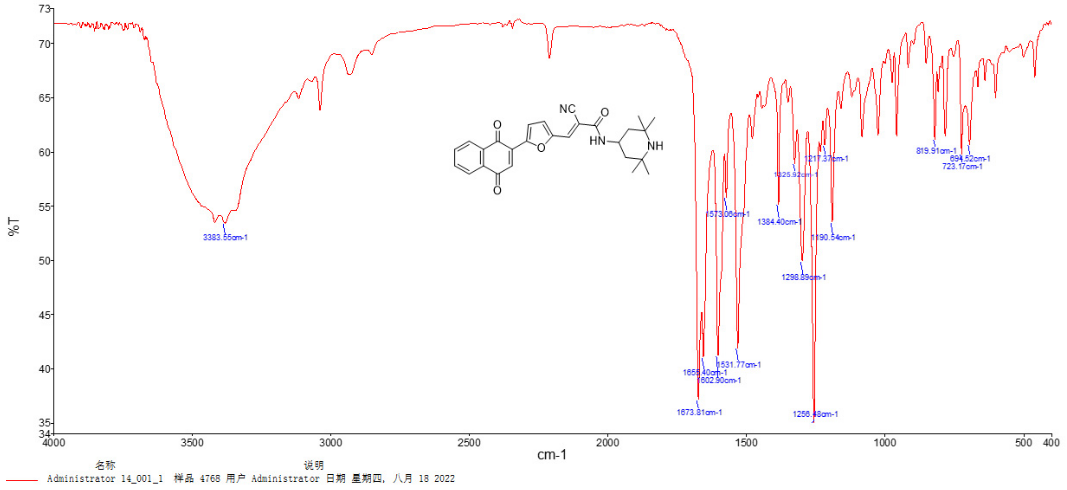

IR of compound **5e**

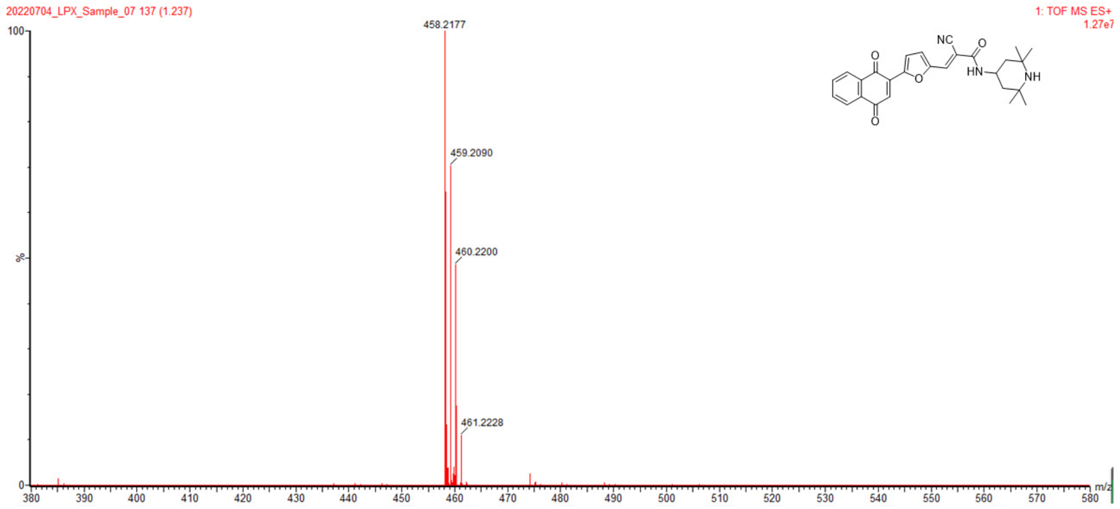

HRMS of compound **5e**

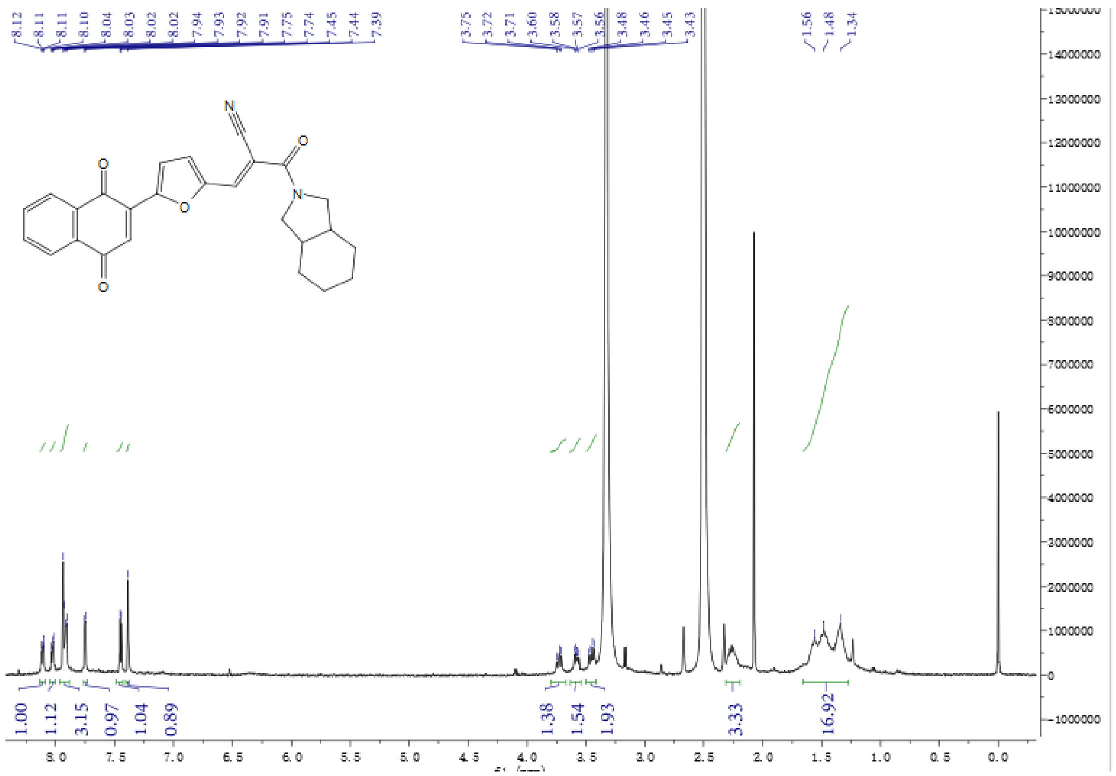

^{1}H-NMR of compound **5f**

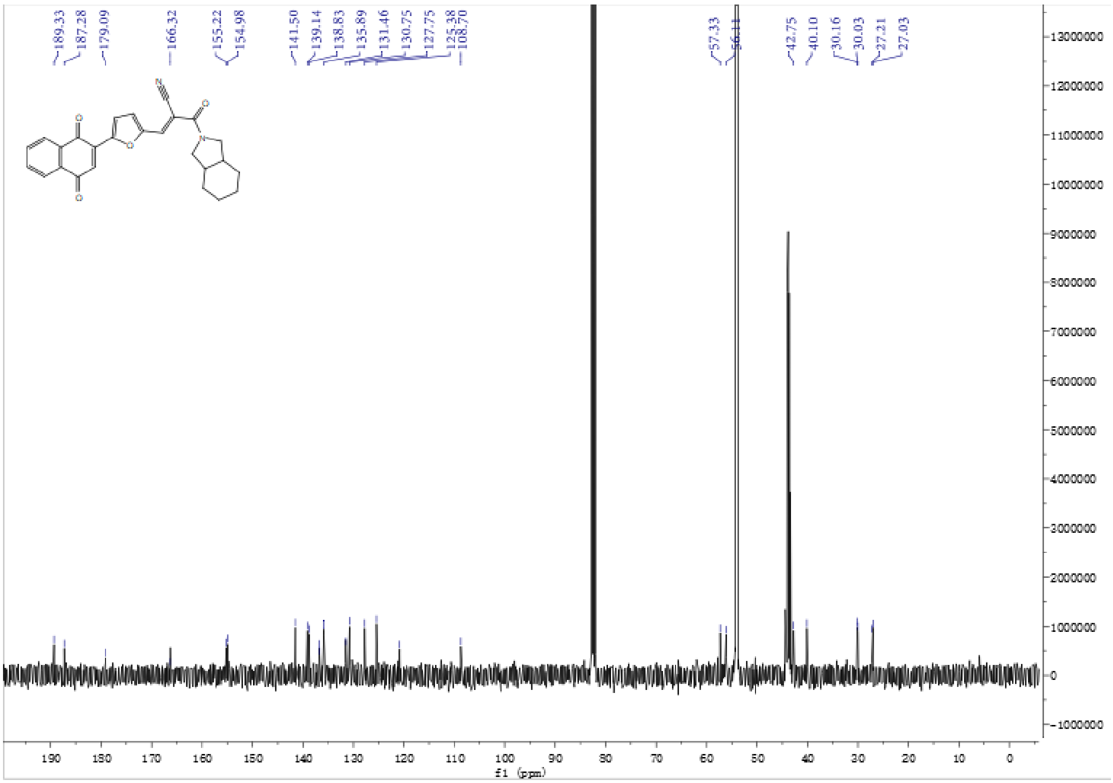

^{13}C-NMR of compound **5f**

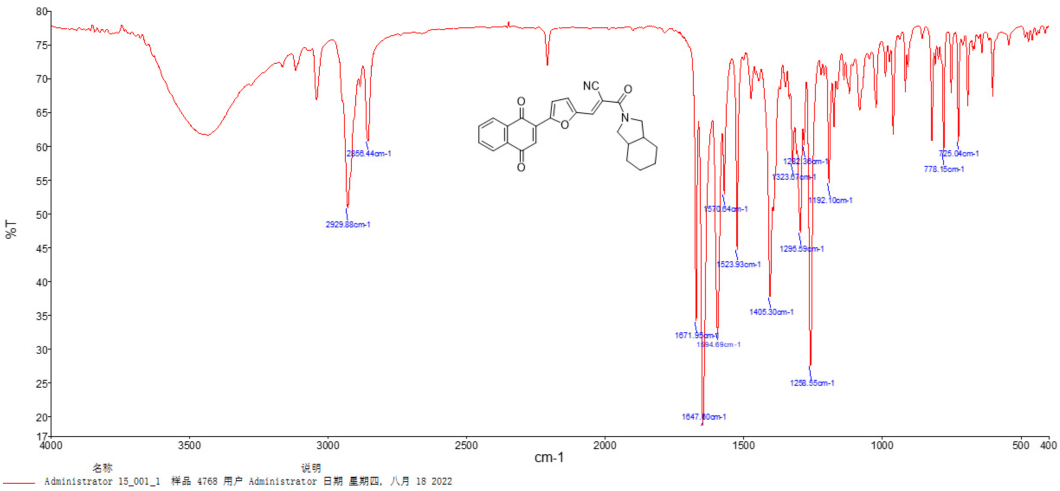

IR of compound **5f**

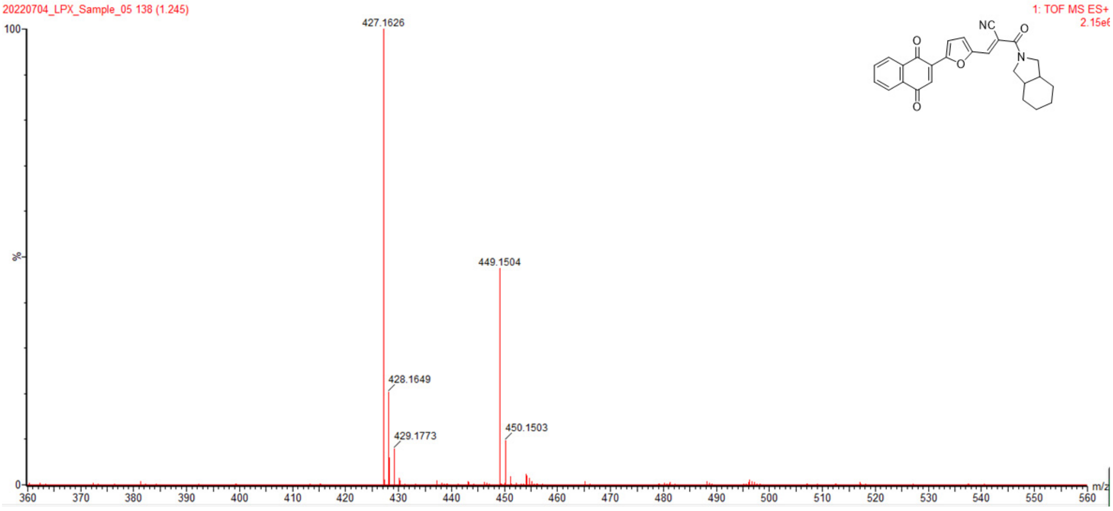

HRMS of compound **5f**

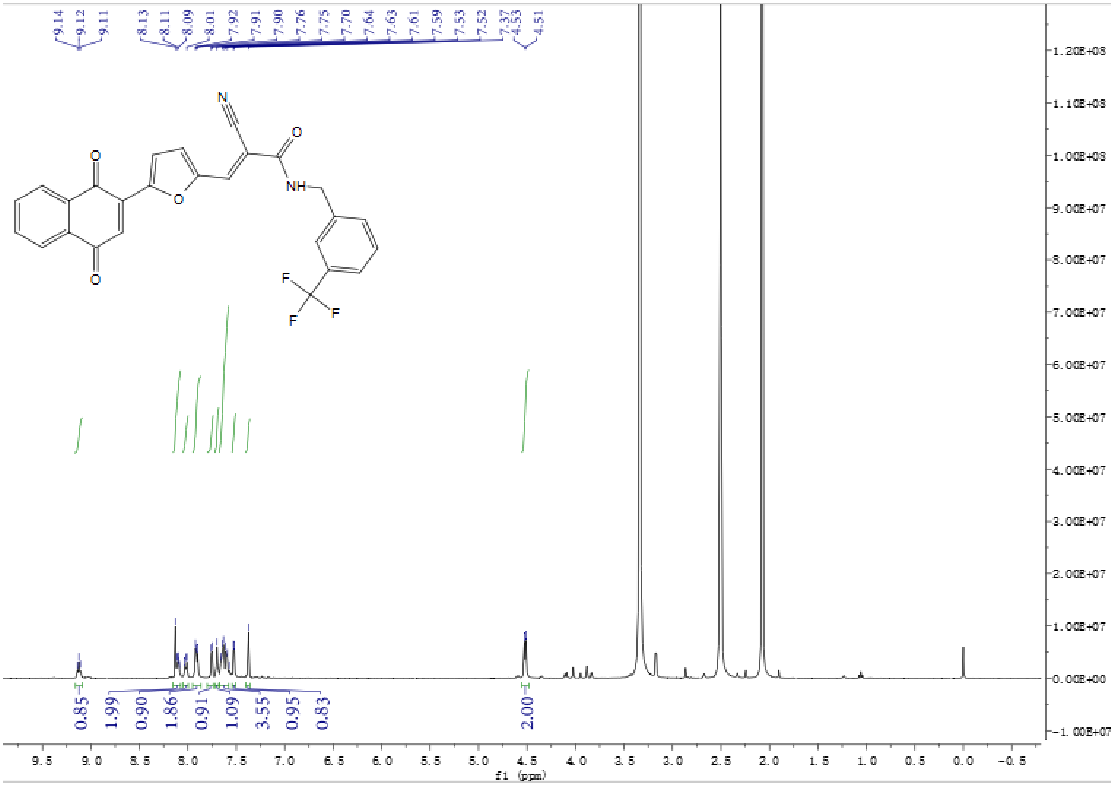

^{1}H-NMR of compound **5g**

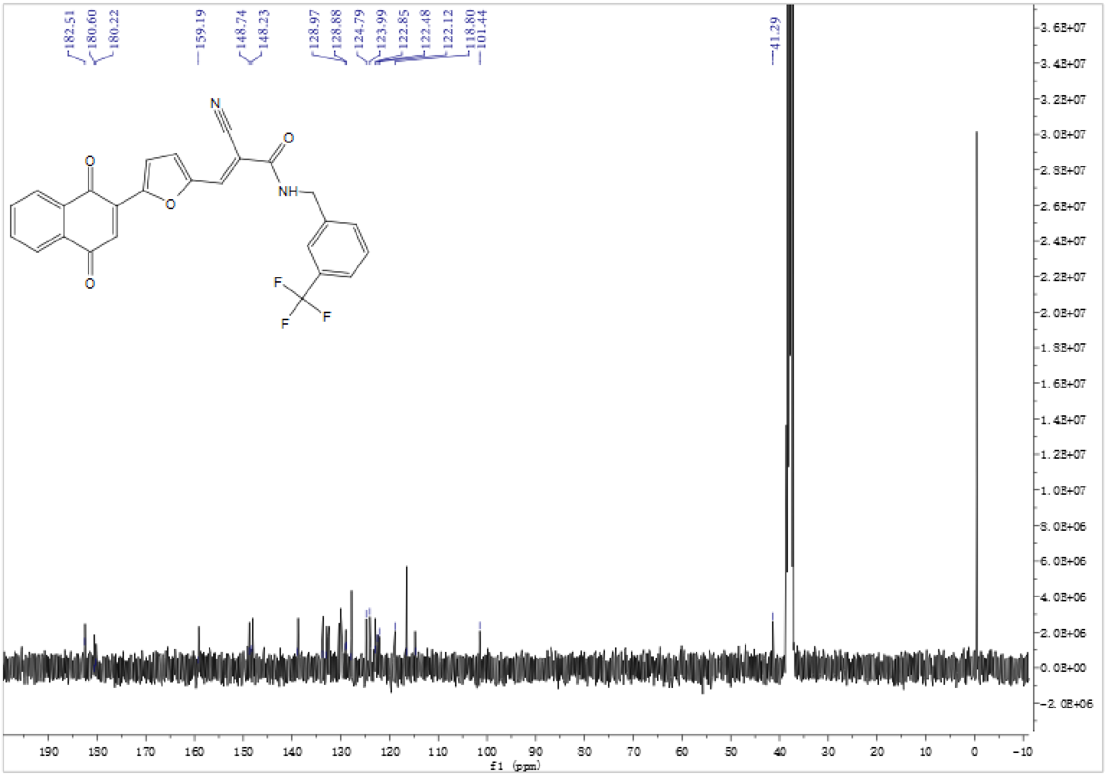

^{13}C-NMR of compound **5g**

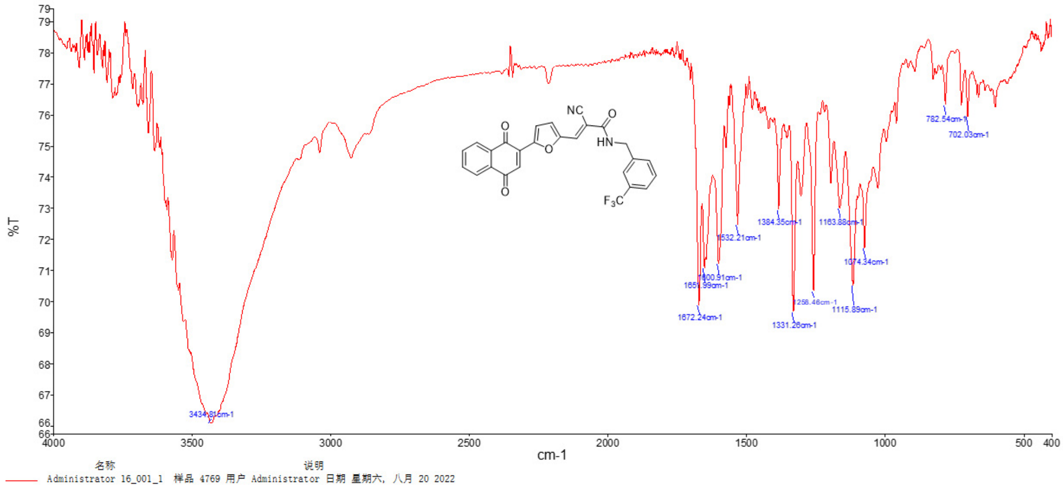

IR of compound **5g**

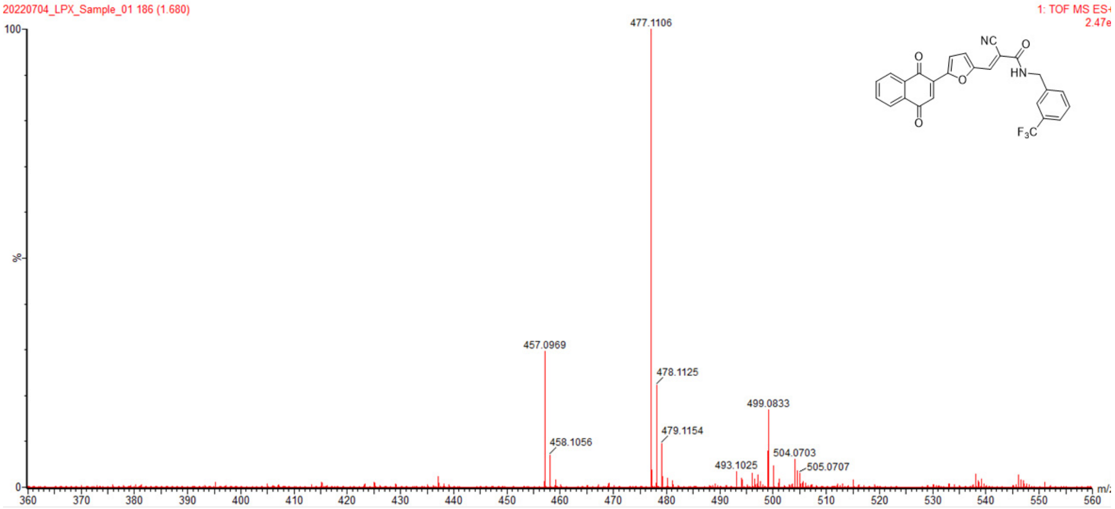

HRMS of compound **5g**

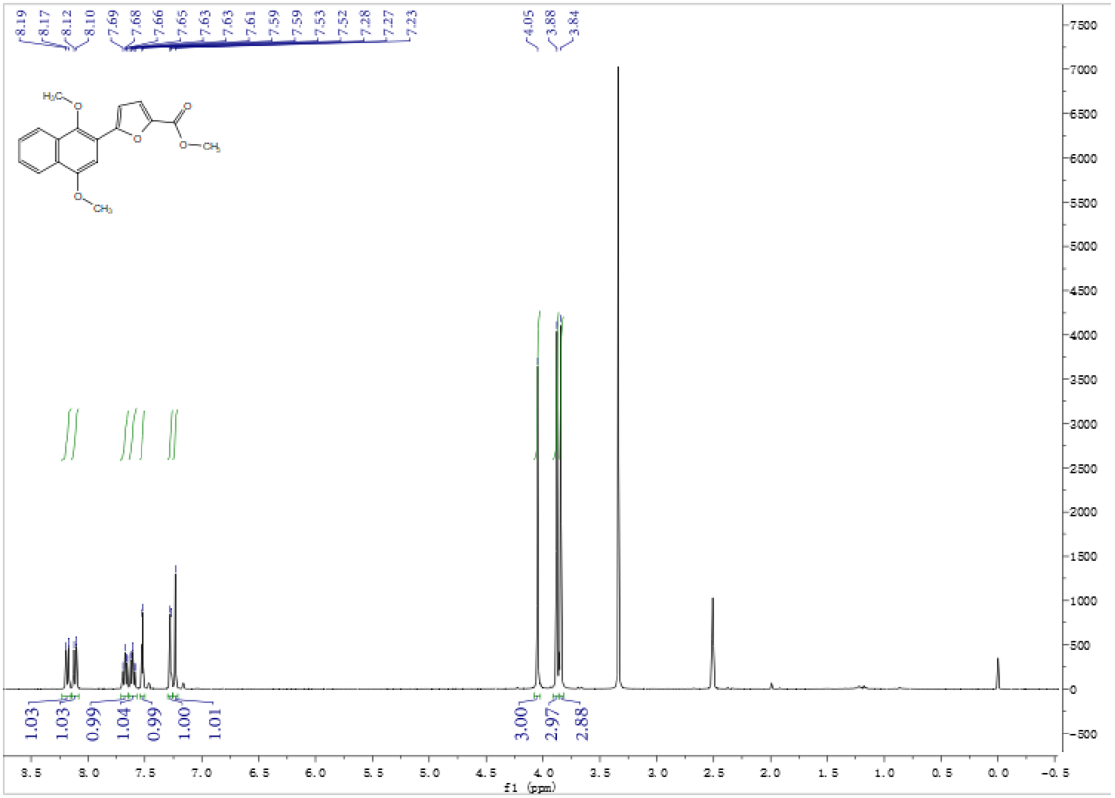

^{1}H-NMR of compound **6**

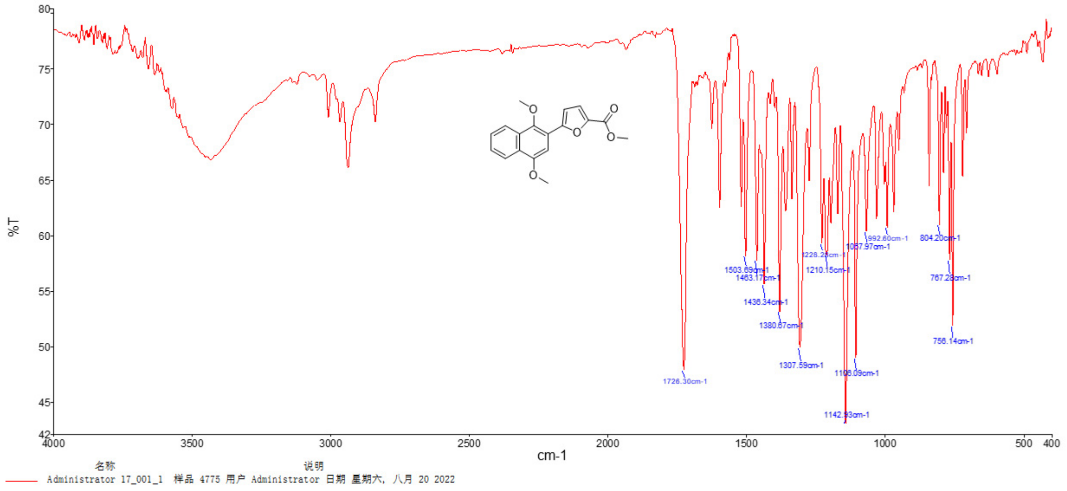

IR of compound **6**

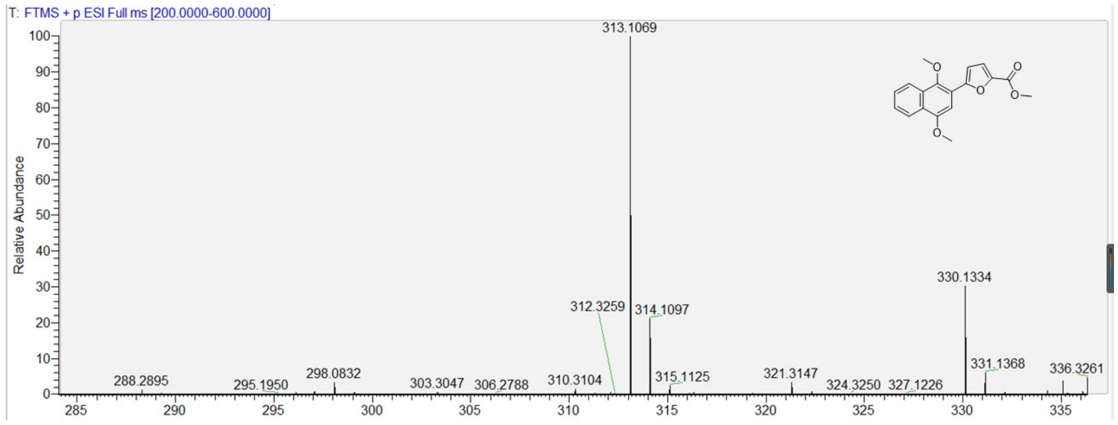

HRMS of compound **6**

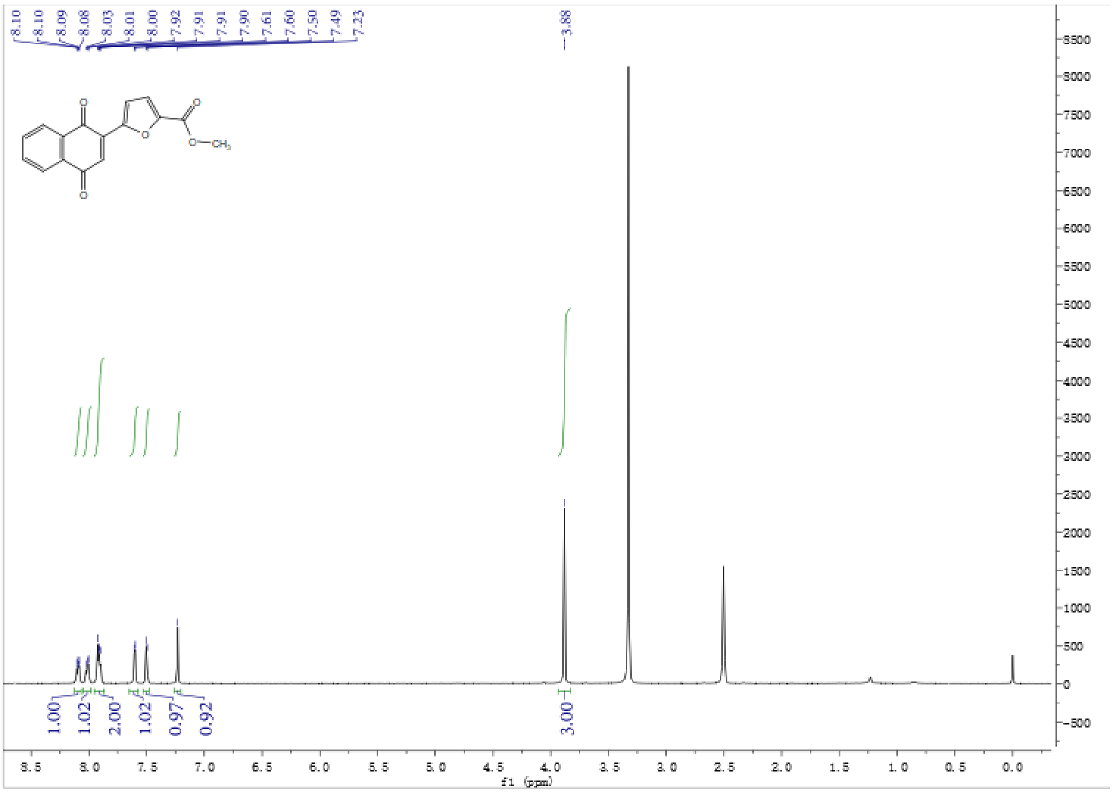

^{1}H-NMR of compound **6a**

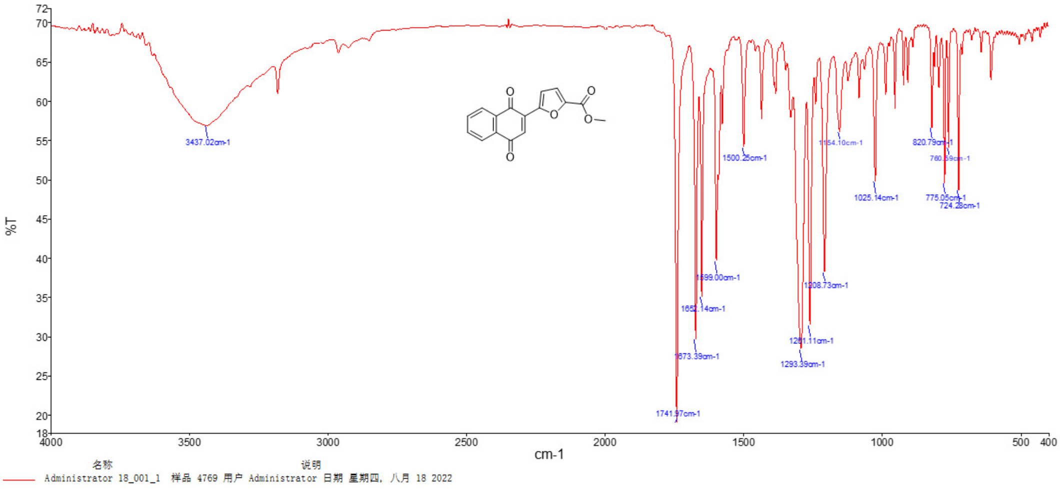

IR of compound **6a**

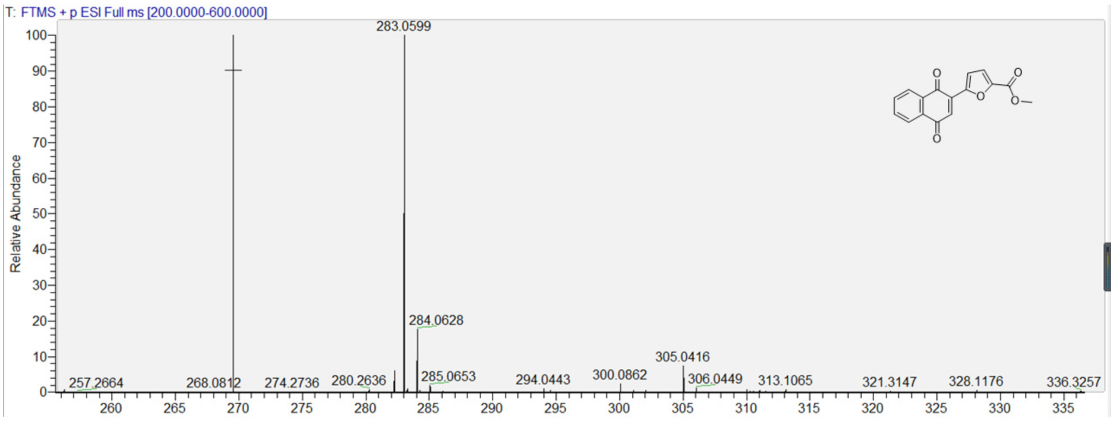

HRMS of compound **6a**

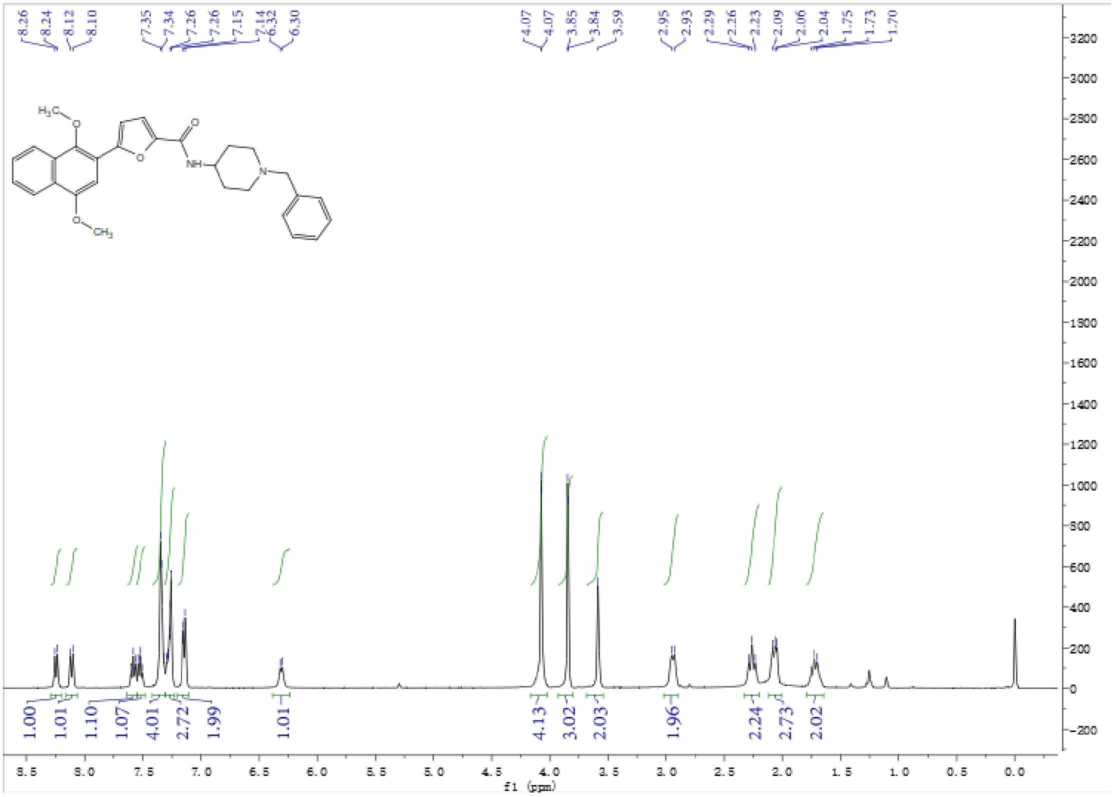

^{1}H-NMR of compound **8a**

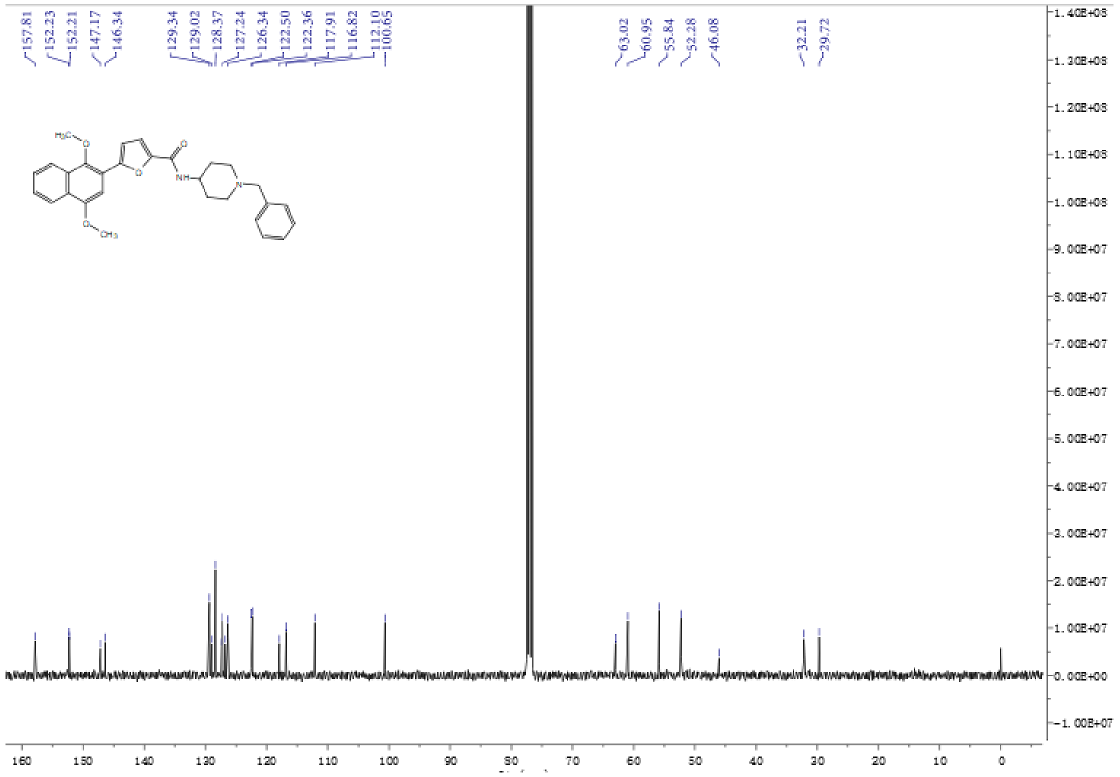

^{13}C-NMR of compound **8a**

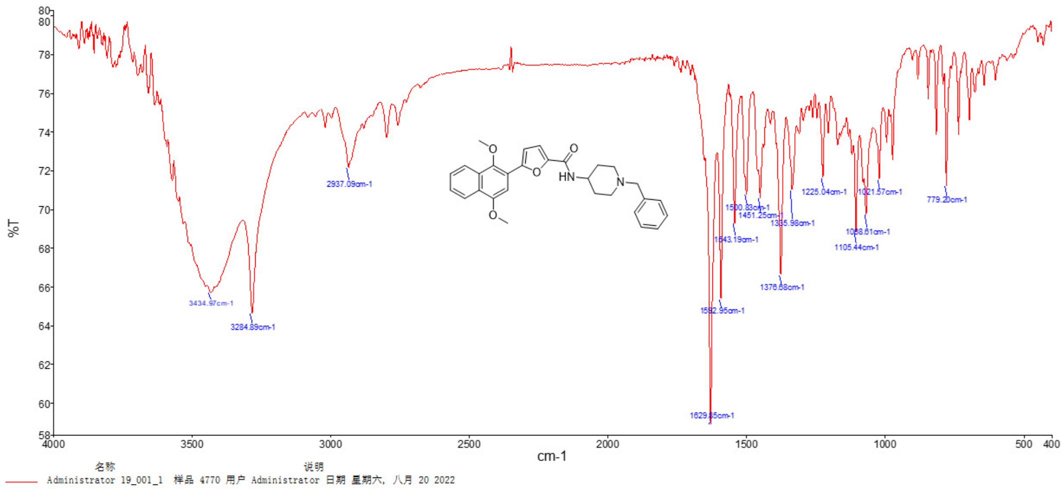

IR of compound **8a**

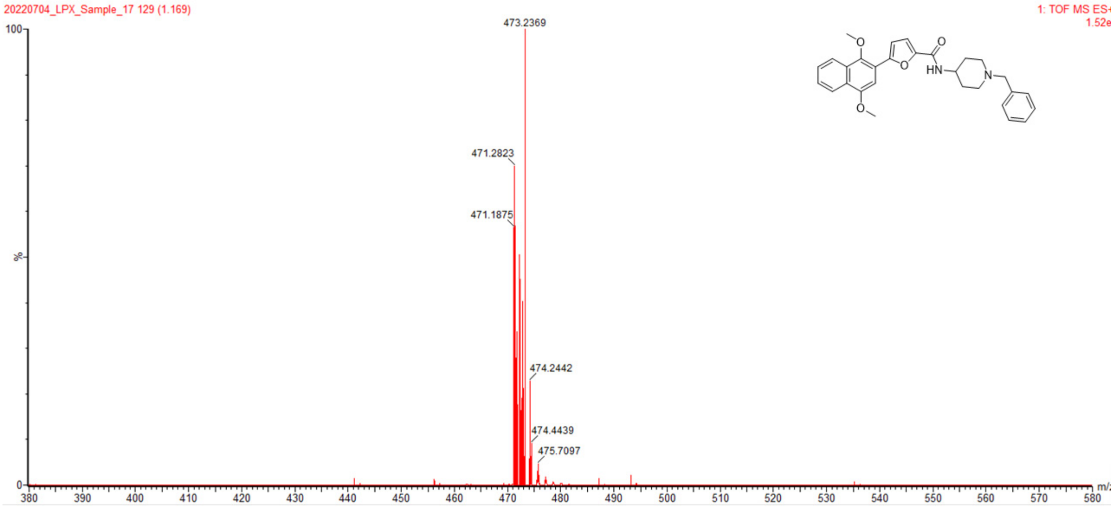

HRMS of compound **8a**

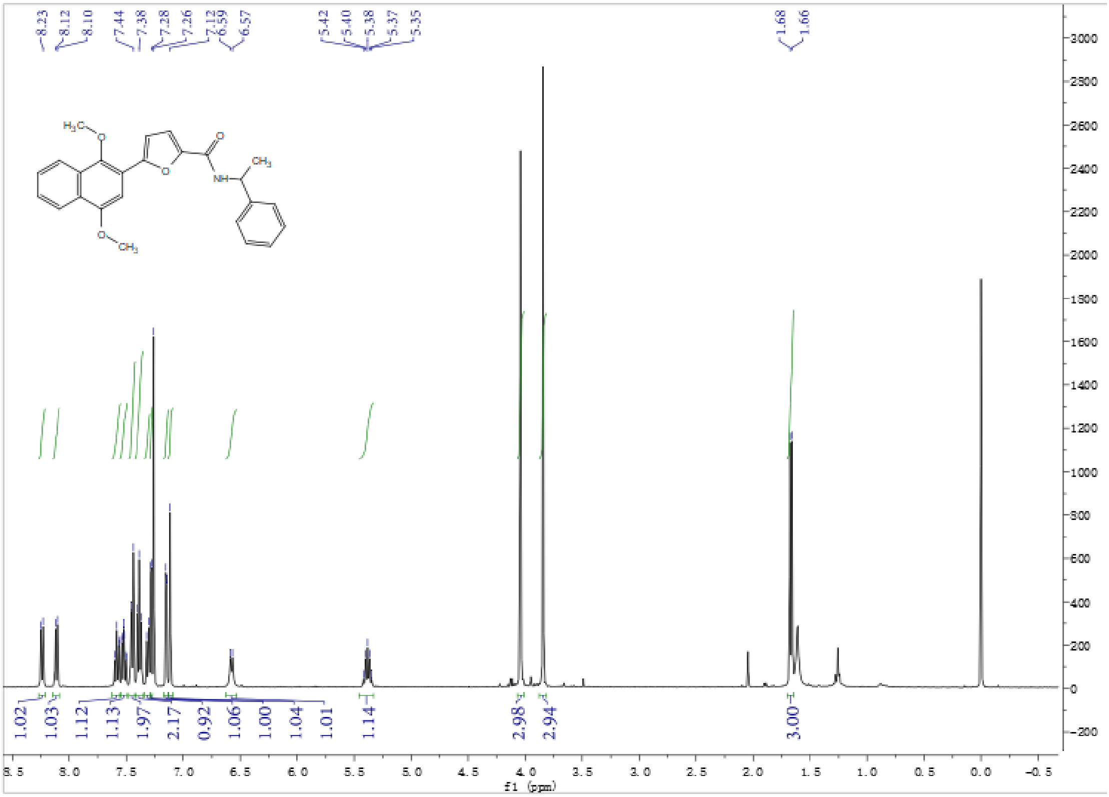

^{1}H-NMR of compound **8b**

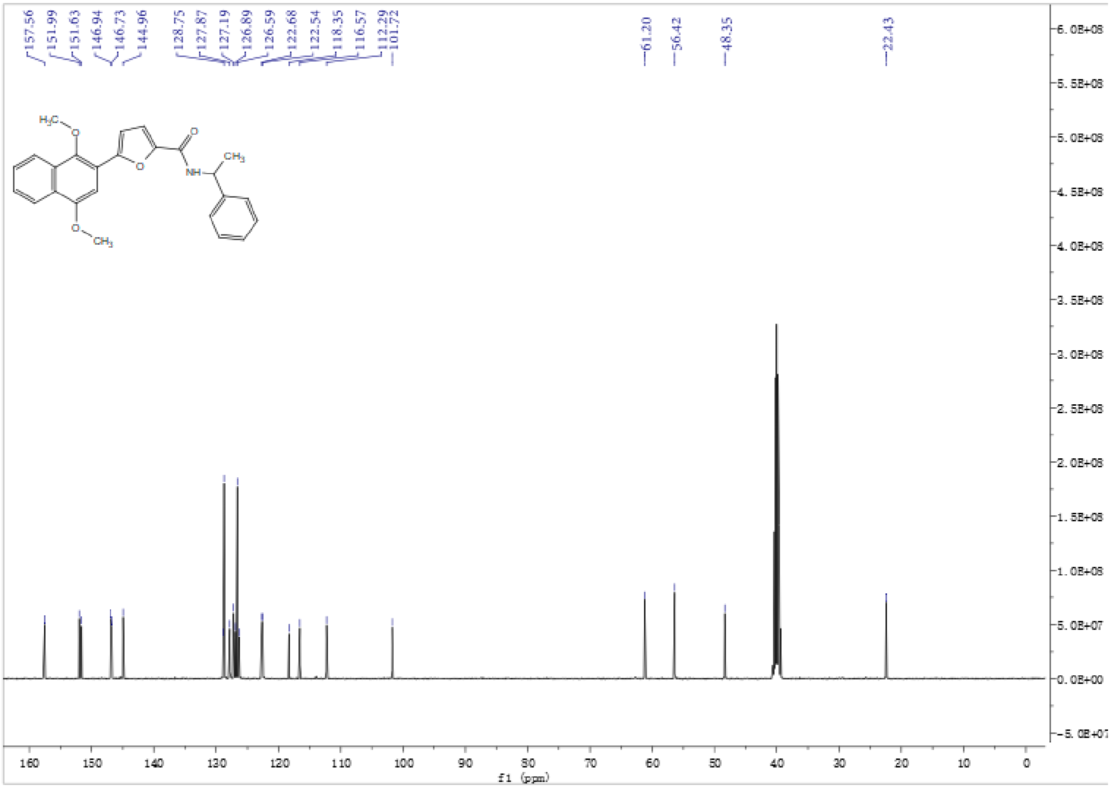

^{13}C-NMR of compound **8b**

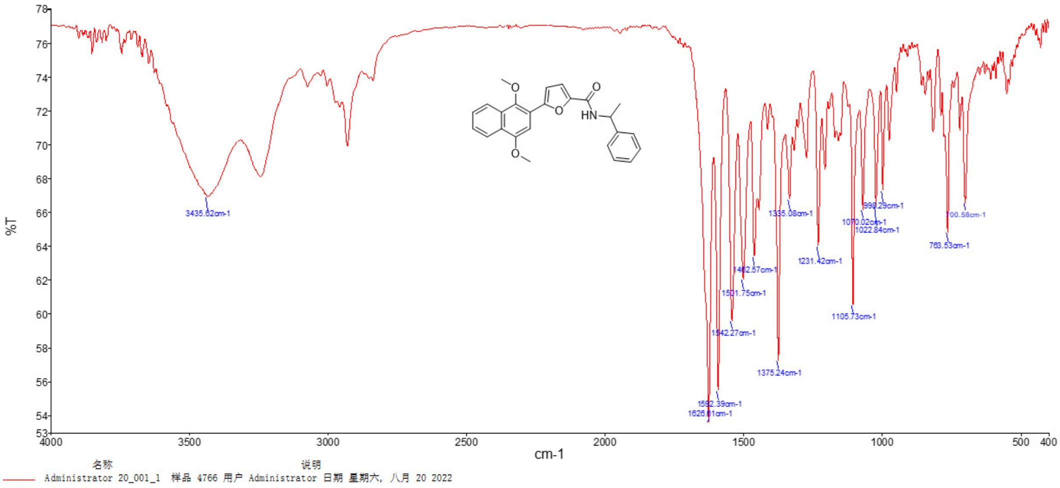

IR of compound **8b**

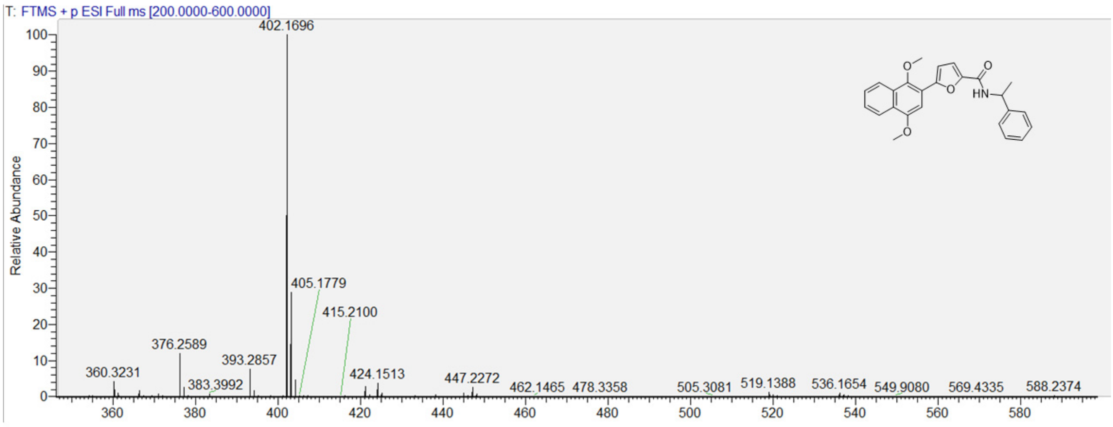

HRMS of compound **8b**

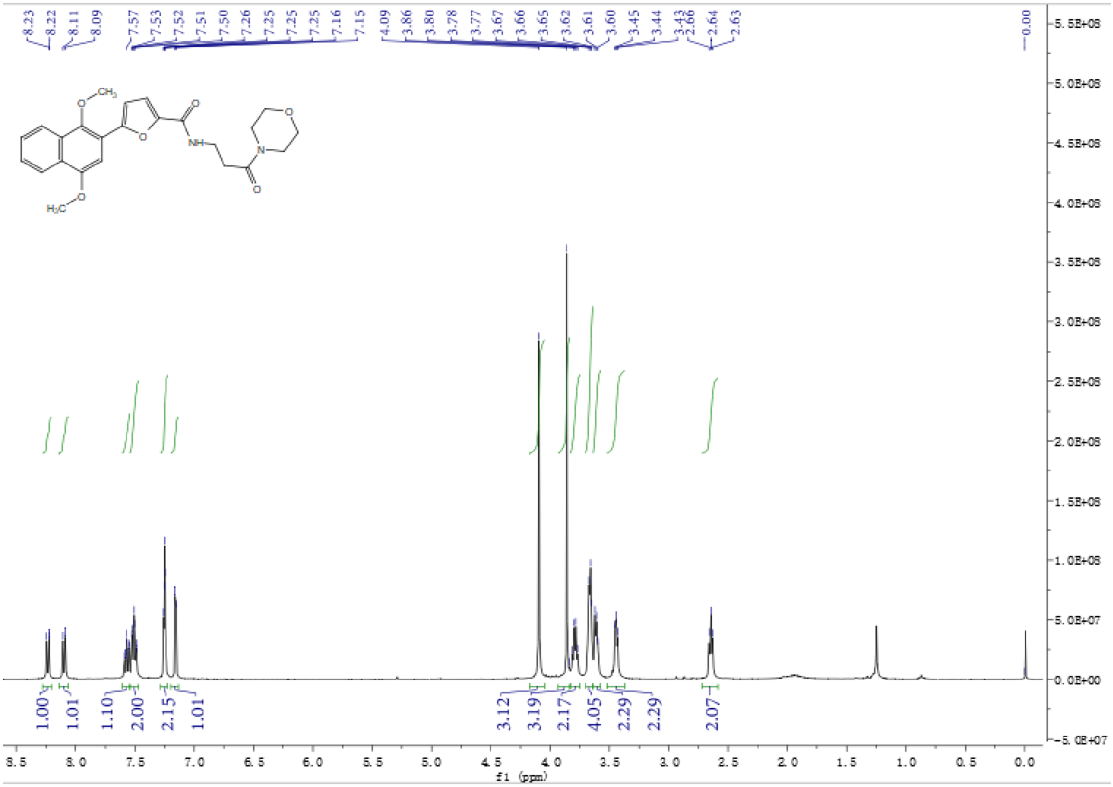

^{1}H-NMR of compound **8c**

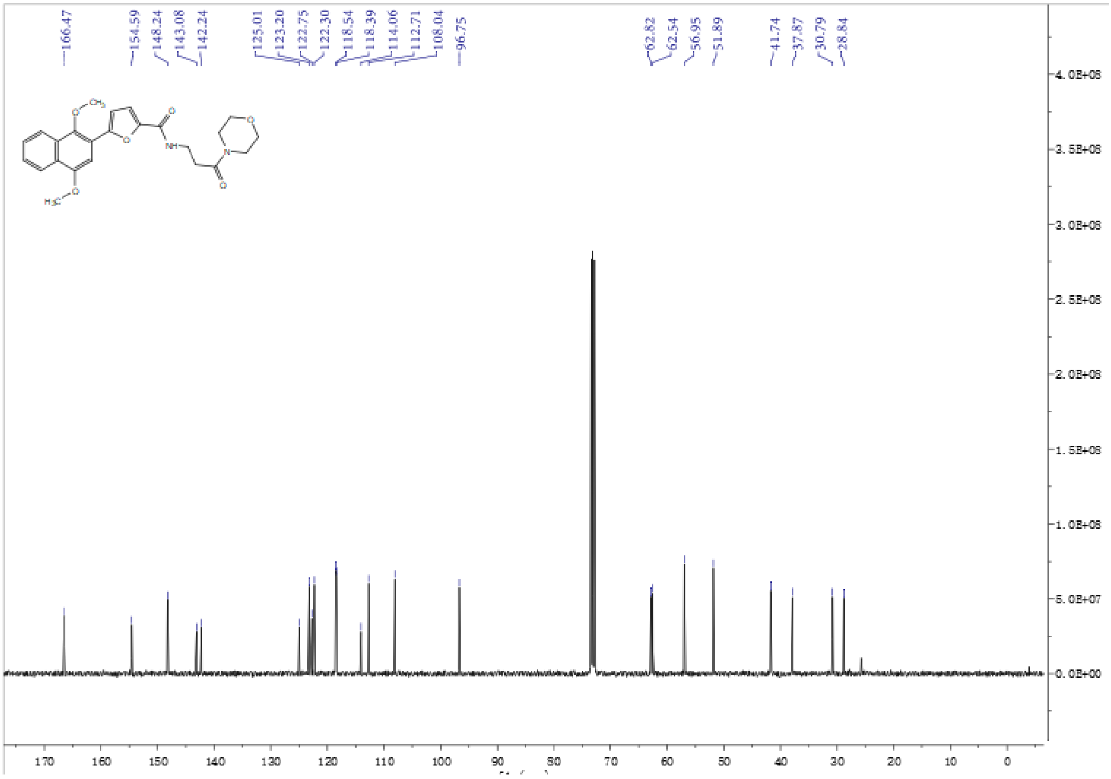

^{13}C-NMR of compound **8c**

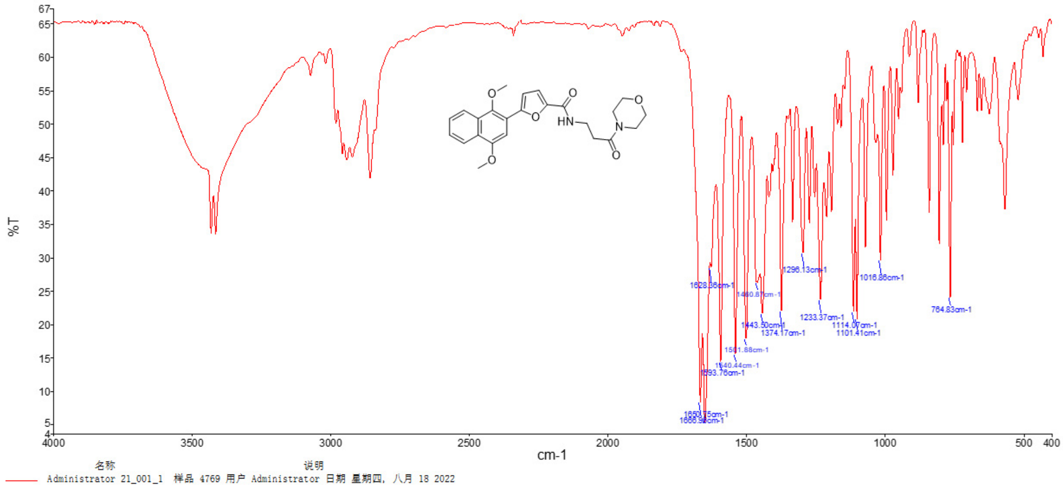

IR of compound **8c**

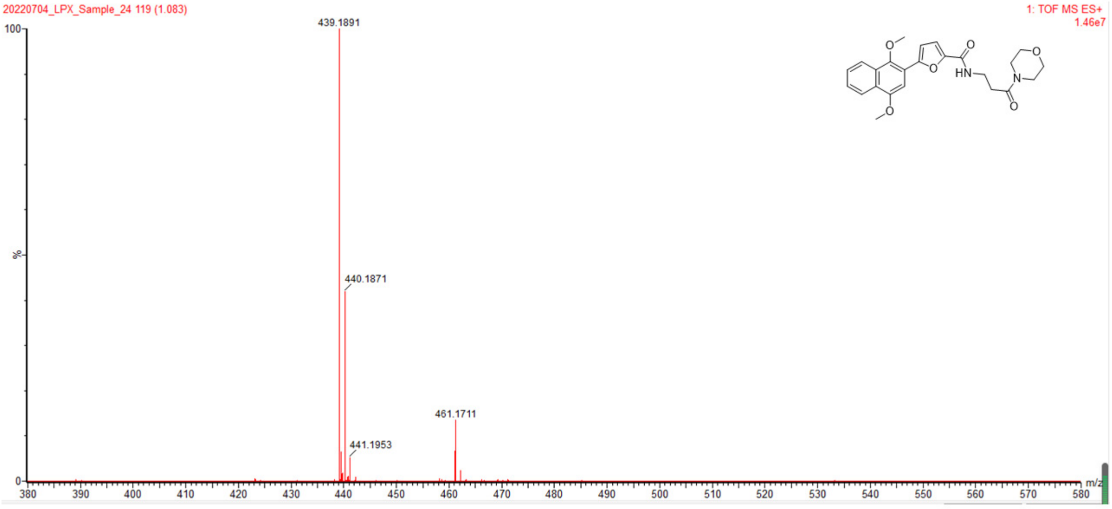

HRMS of compound **8c**

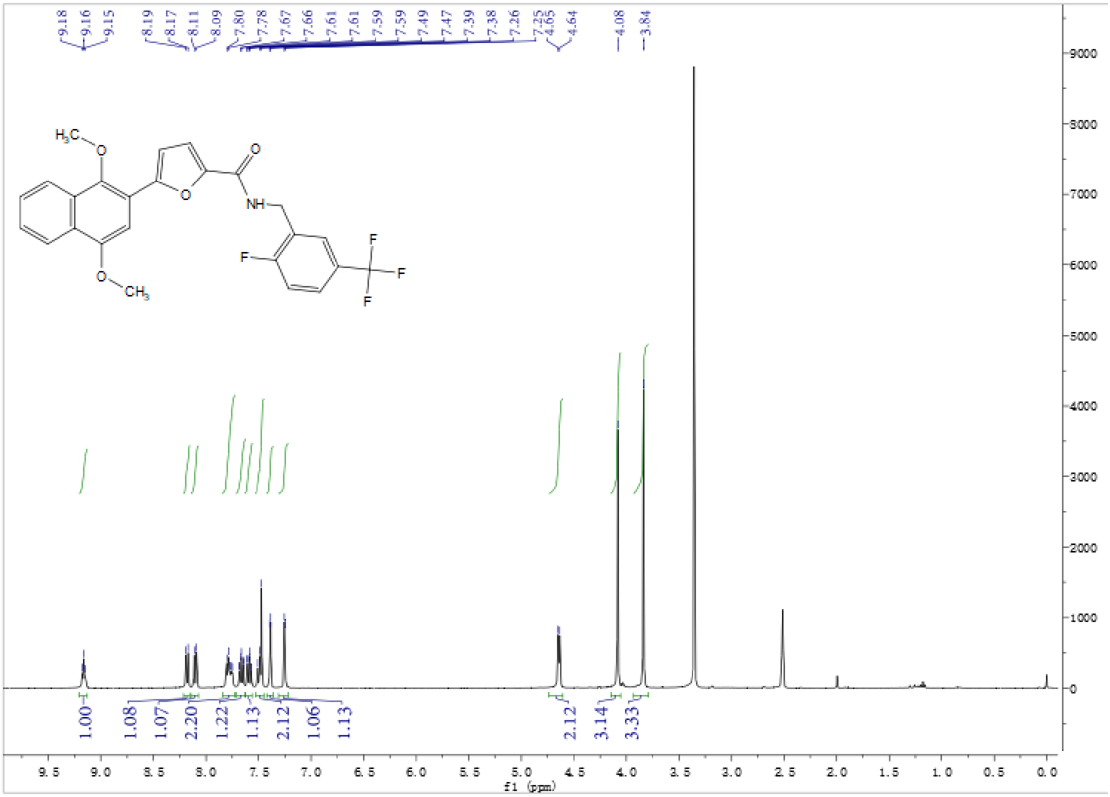

^{1}H-NMR of compound **8d**

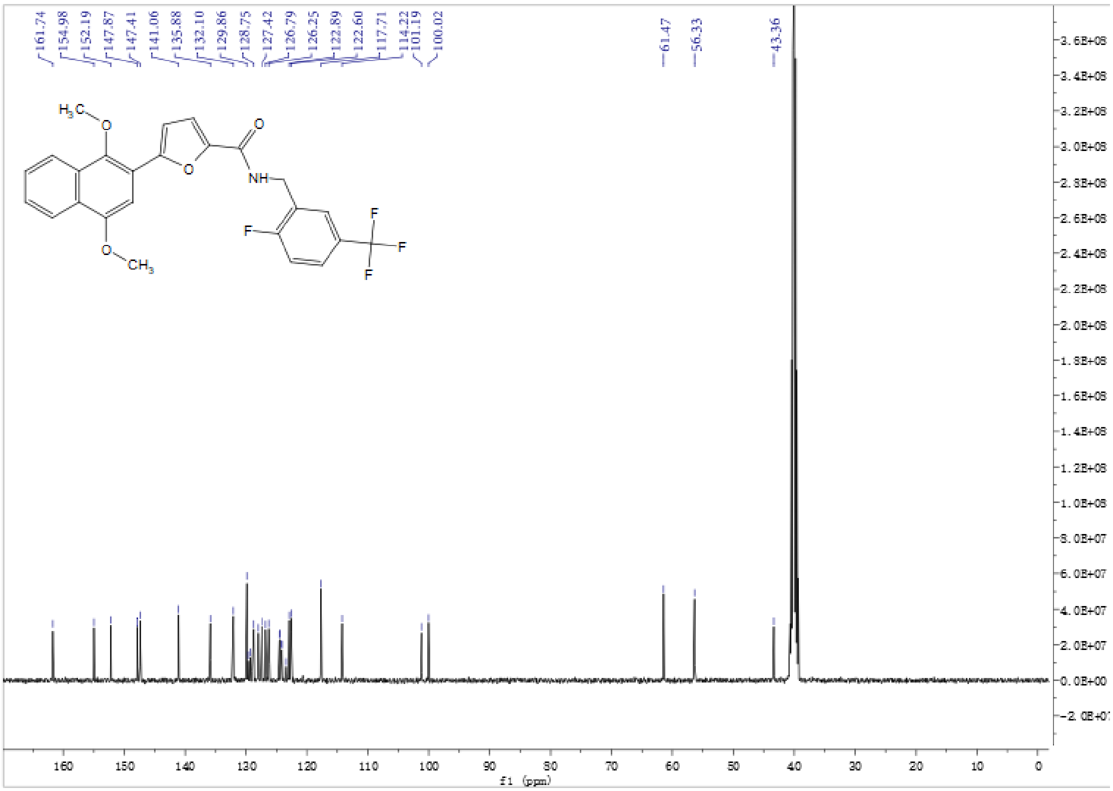

HRMS of compound **8d**

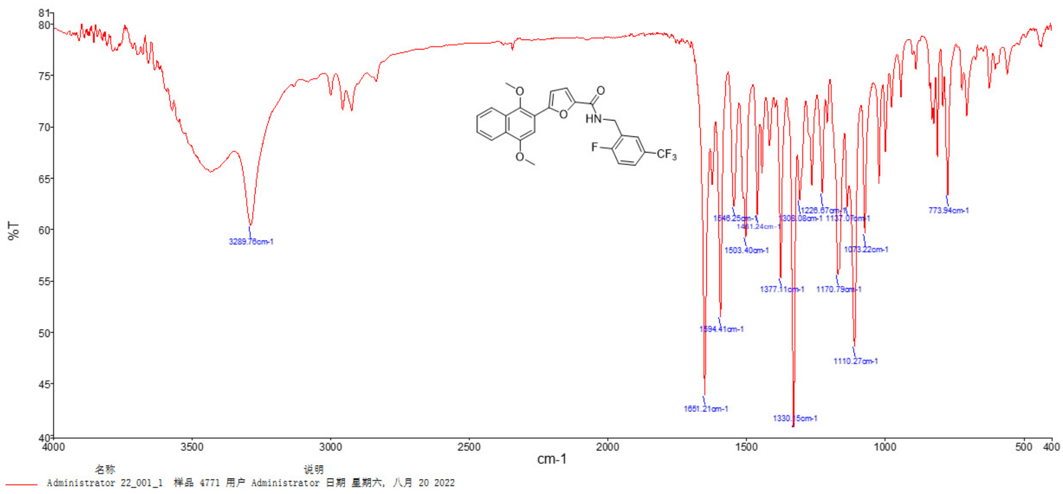

IR of compound **8d**

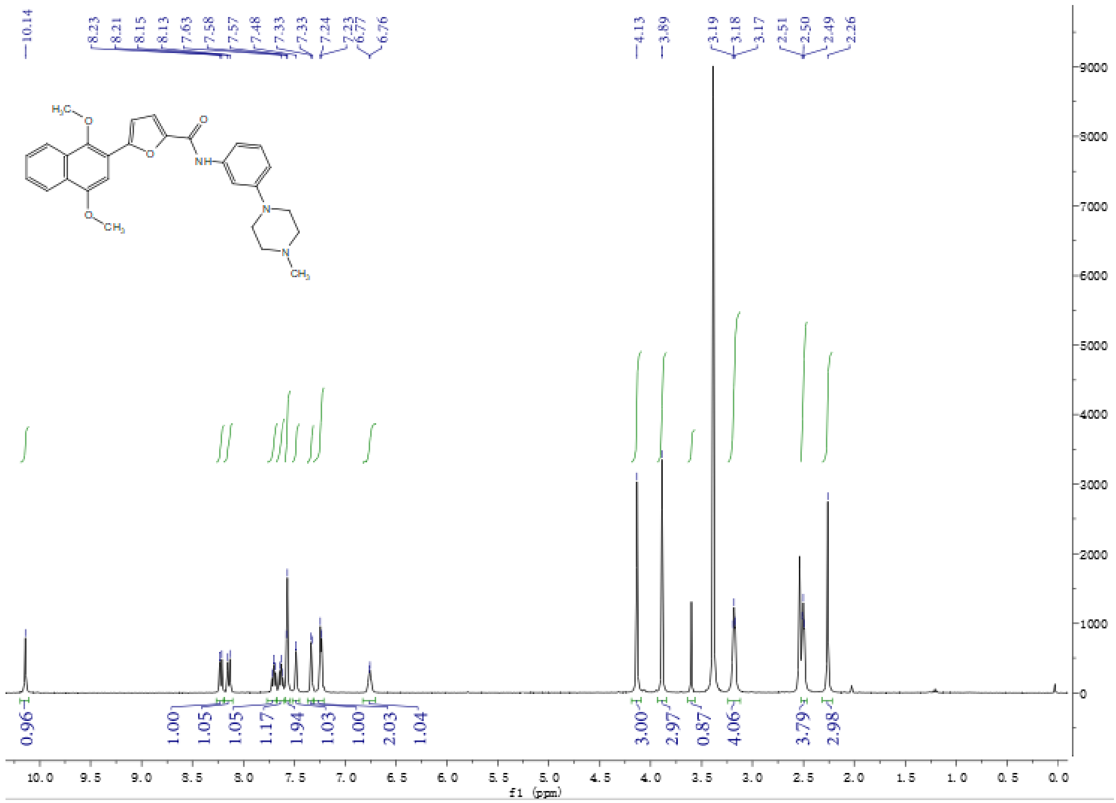

^{1}H-NMR of compound **8e**

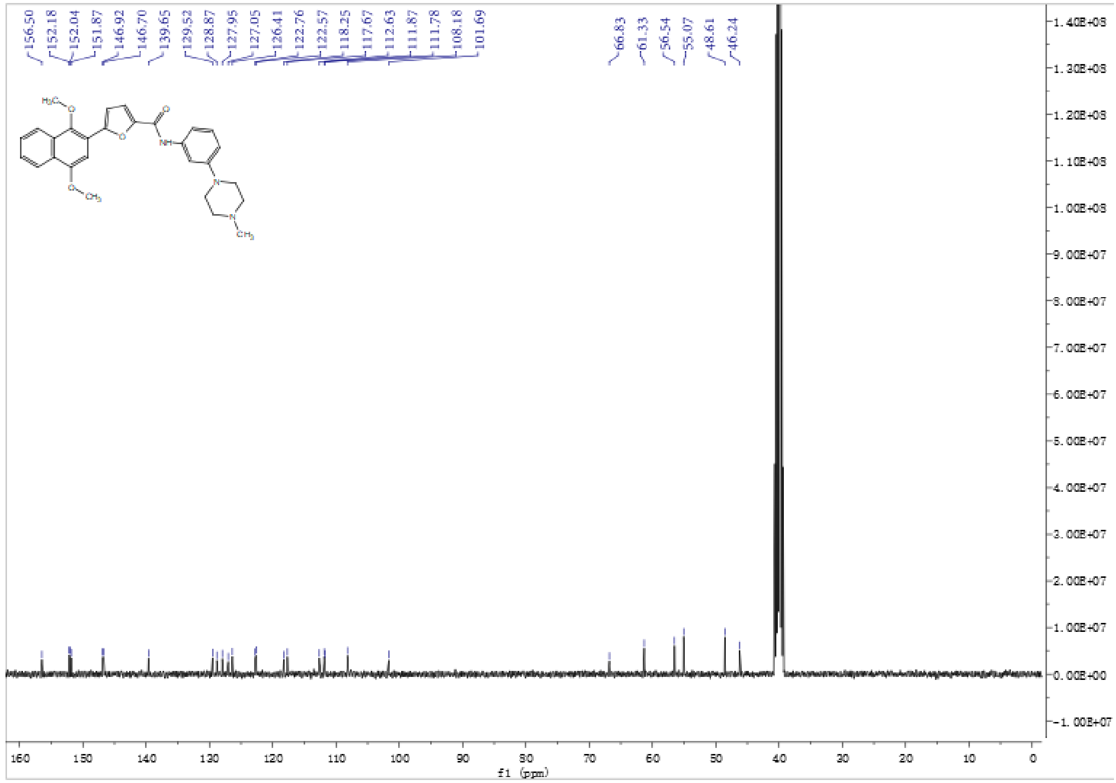

^{13}C-NMR of compound **8e**

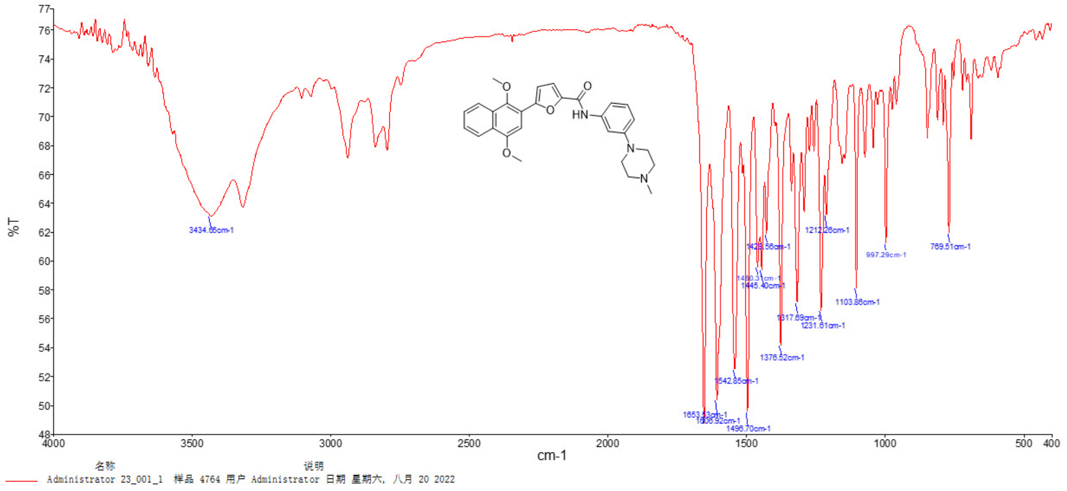

IR of compound **8e**

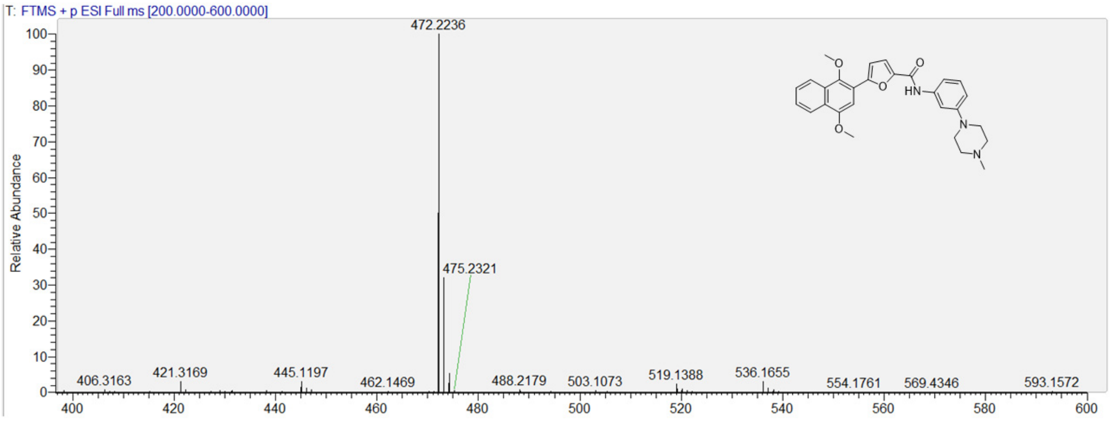

HRMS of compound **8e**

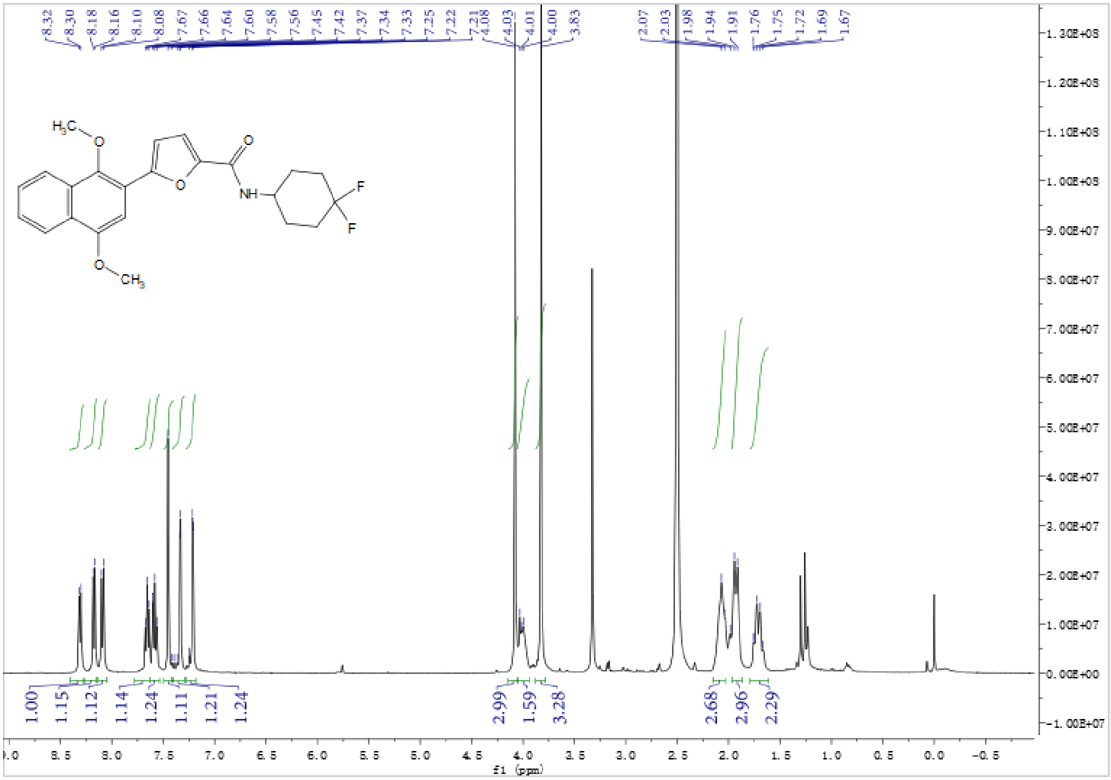

^{1}H-NMR of compound **8f**

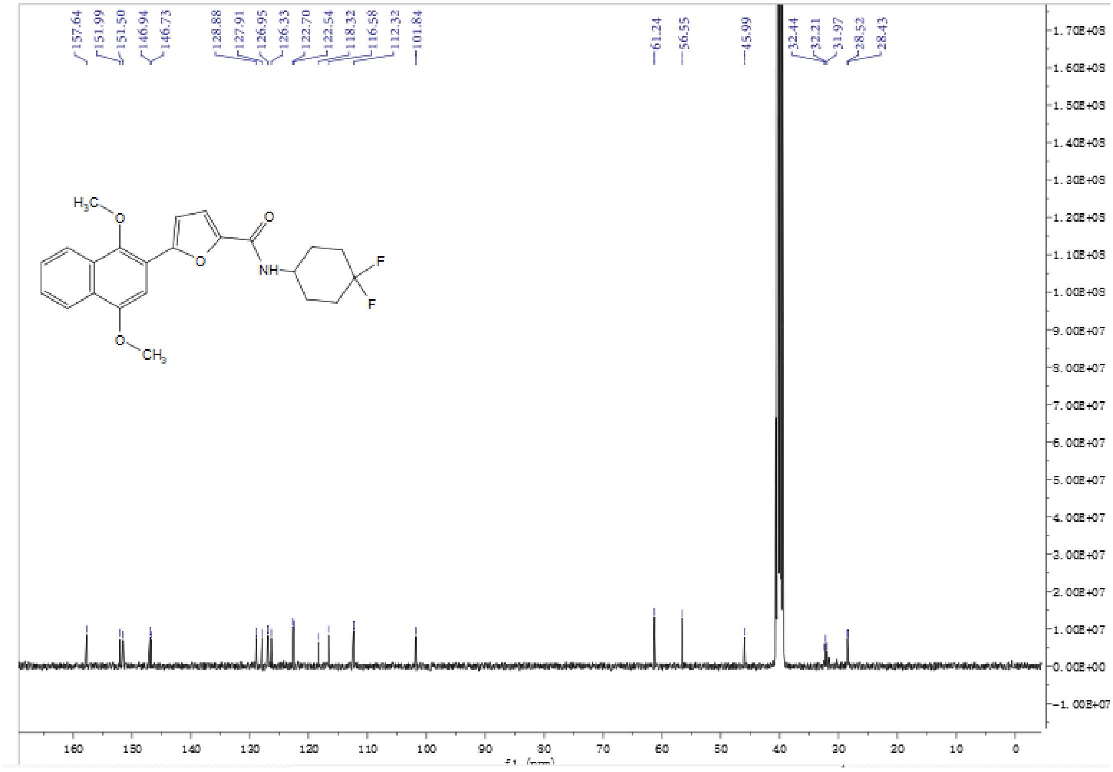

^{13}C-NMR of compound **8f**

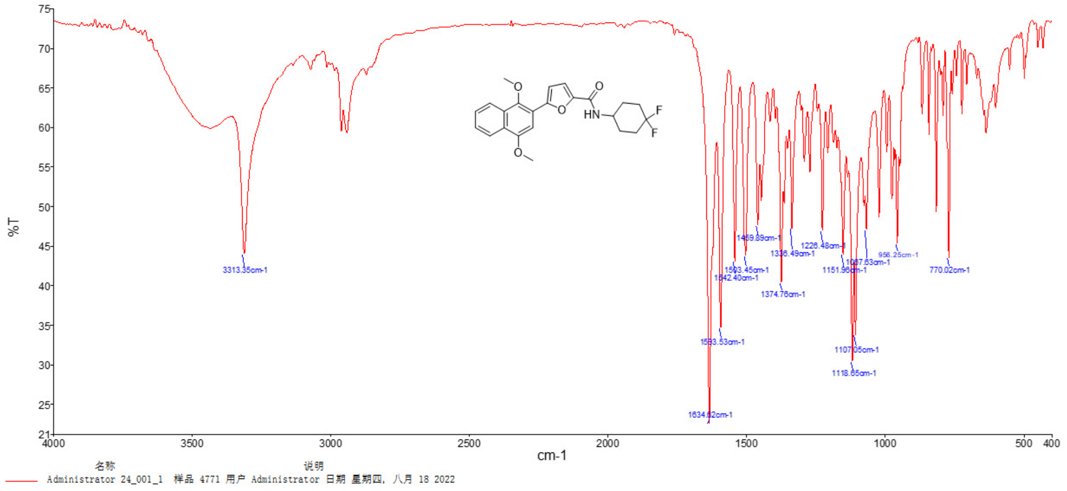

IR of compound **8f**

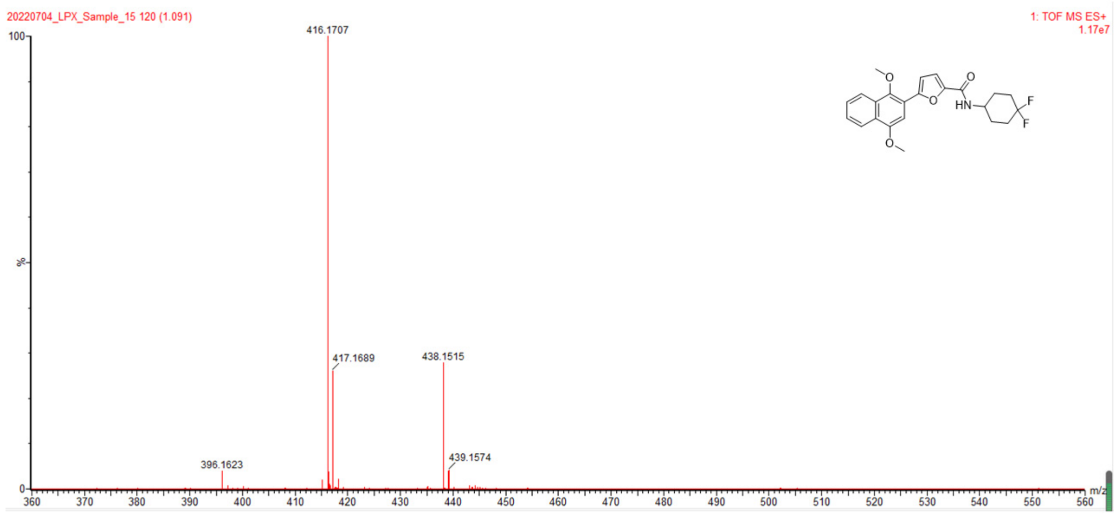

HRMS of compound **8f**

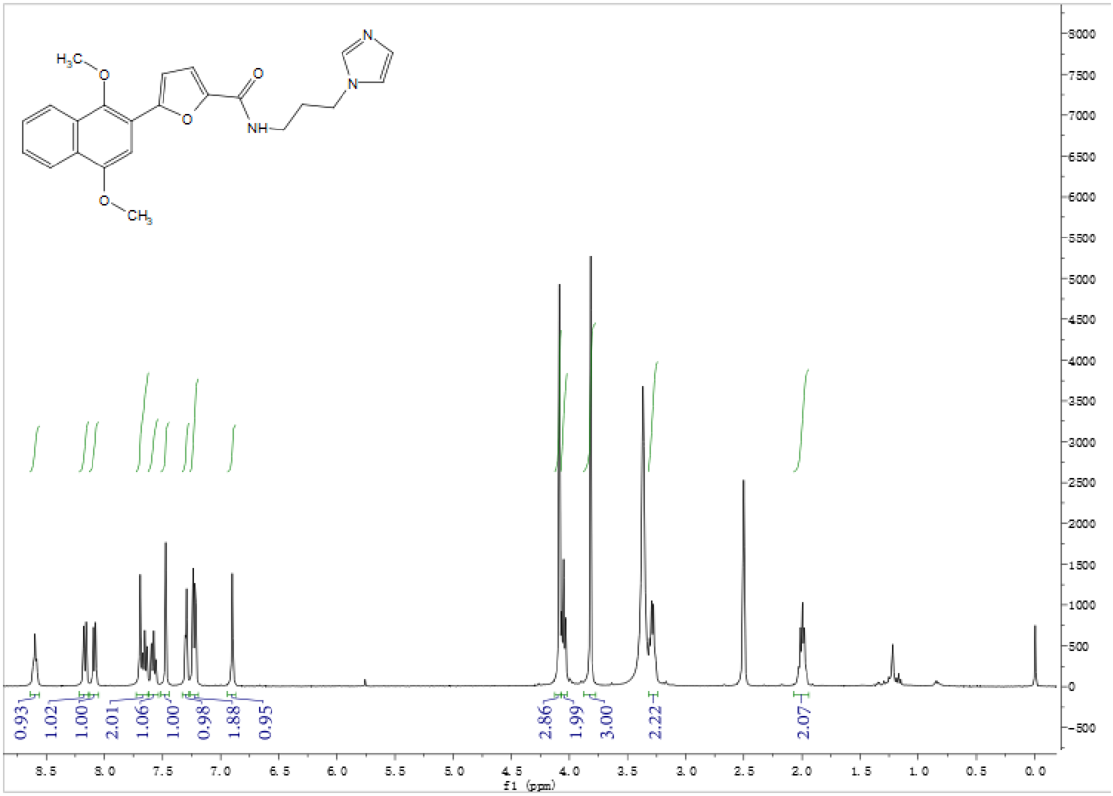

^{1}H-NMR of compound **8g**

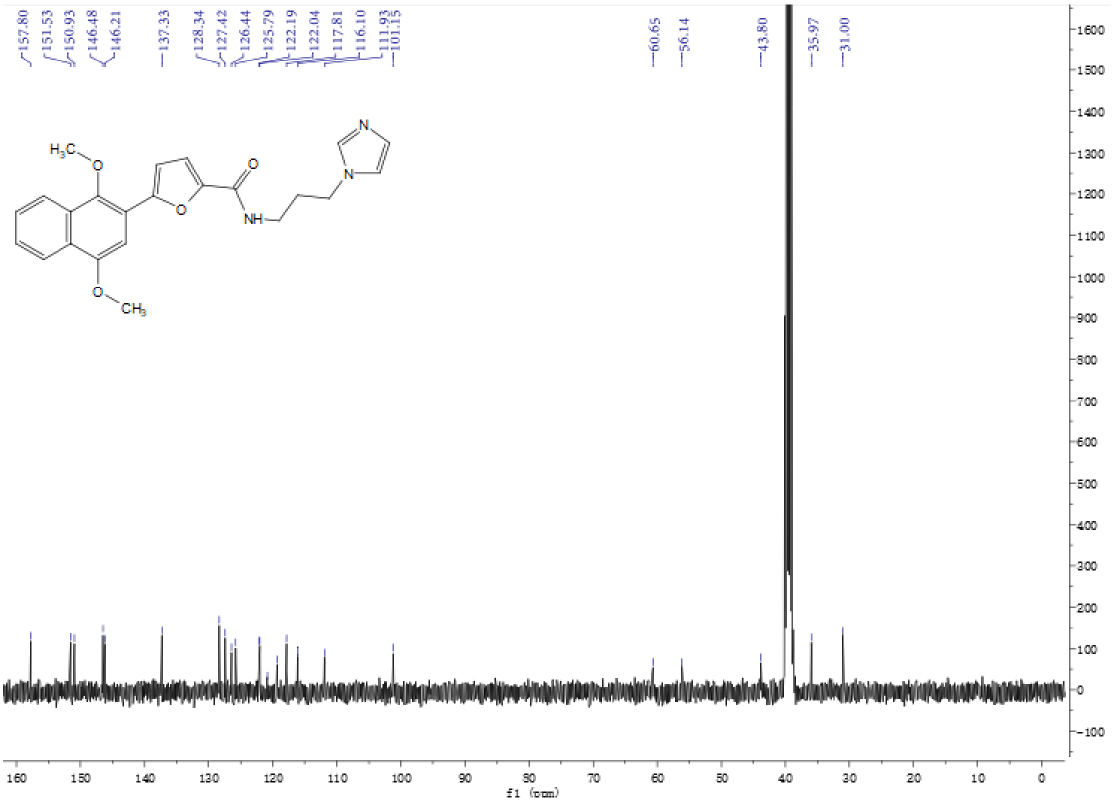

^{13}C-NMR of compound **8g**

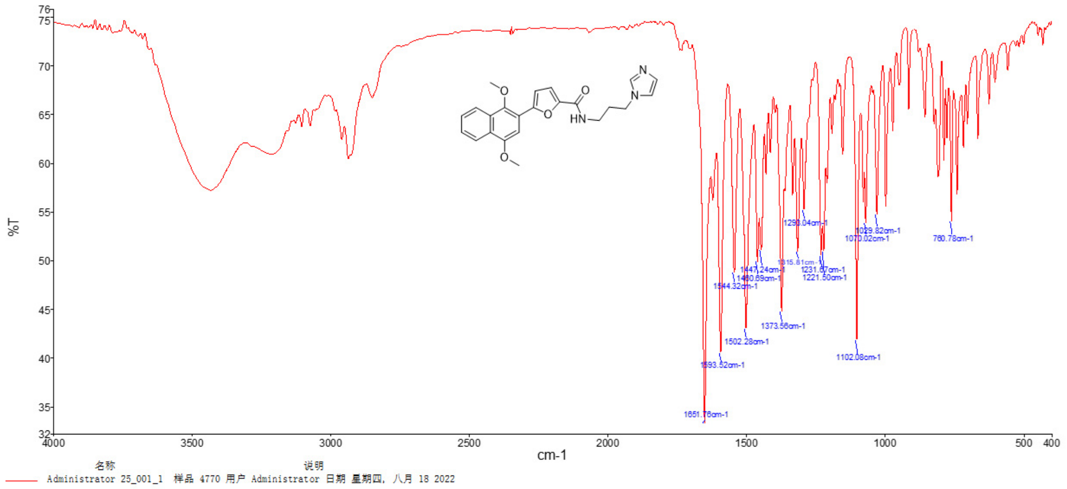

IR of compound **8g**

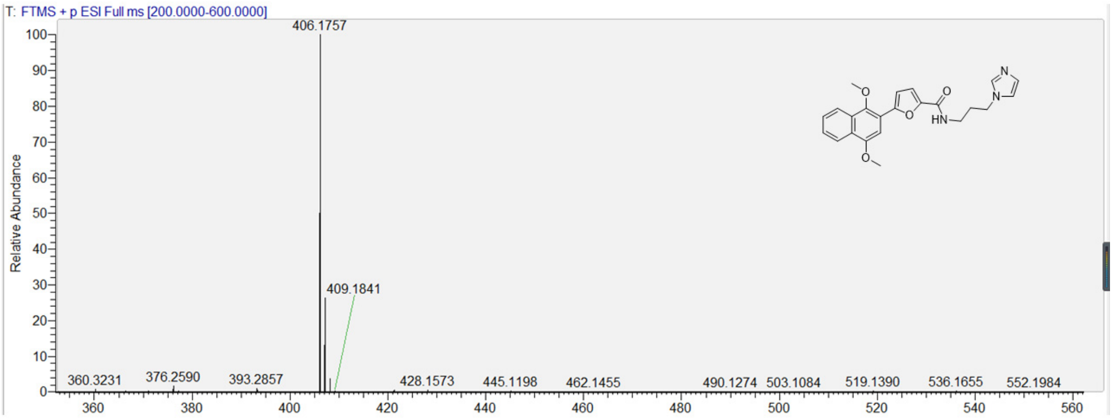

HRMS of compound **8g**

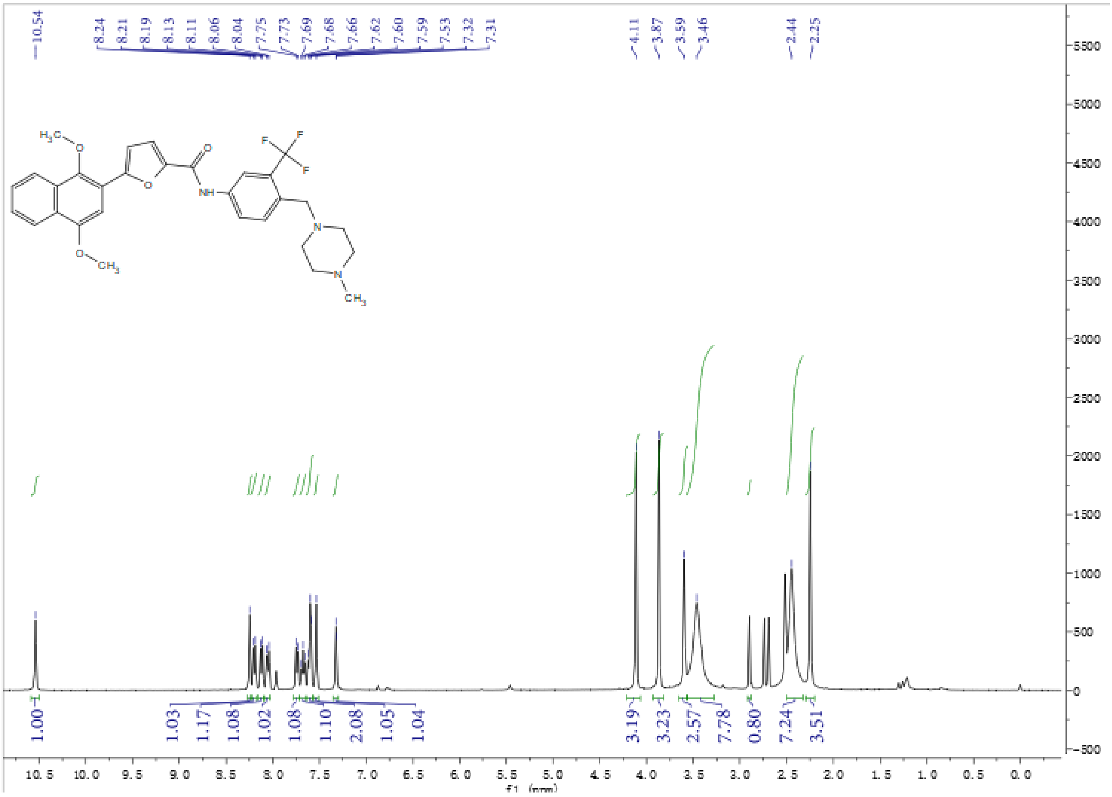

^{1}H-NMR of compound **8h**

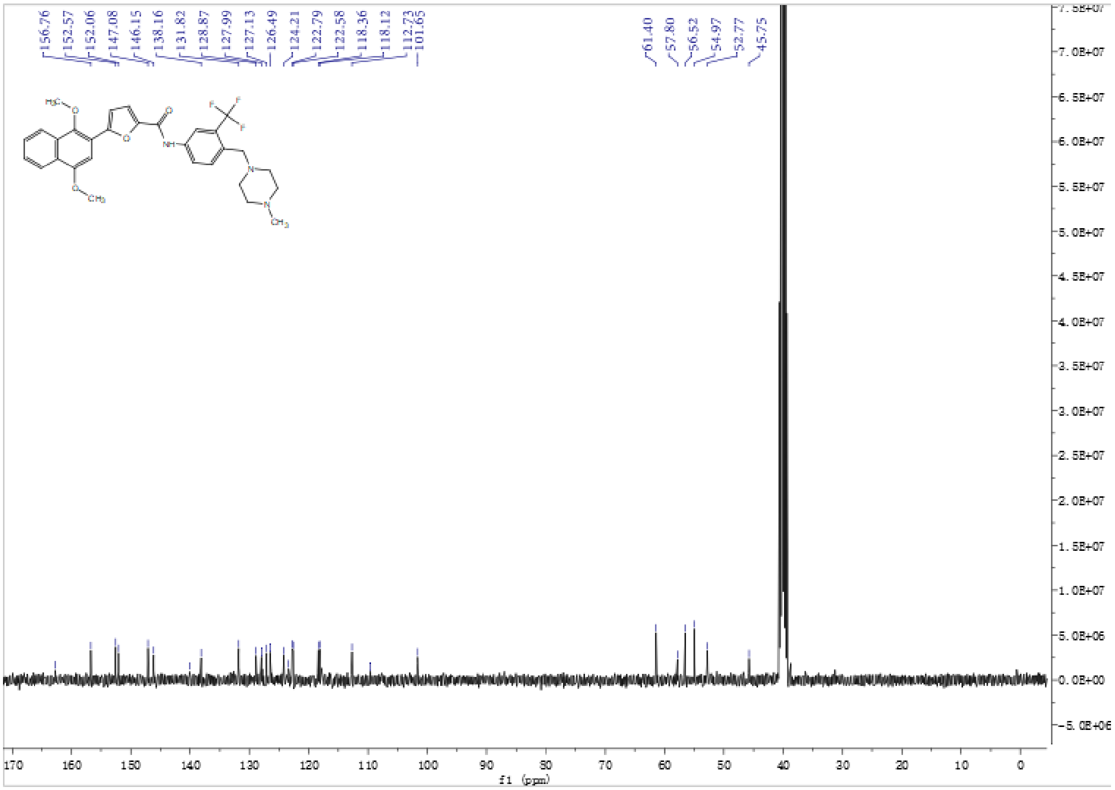

^{13}C-NMR of compound **8h**

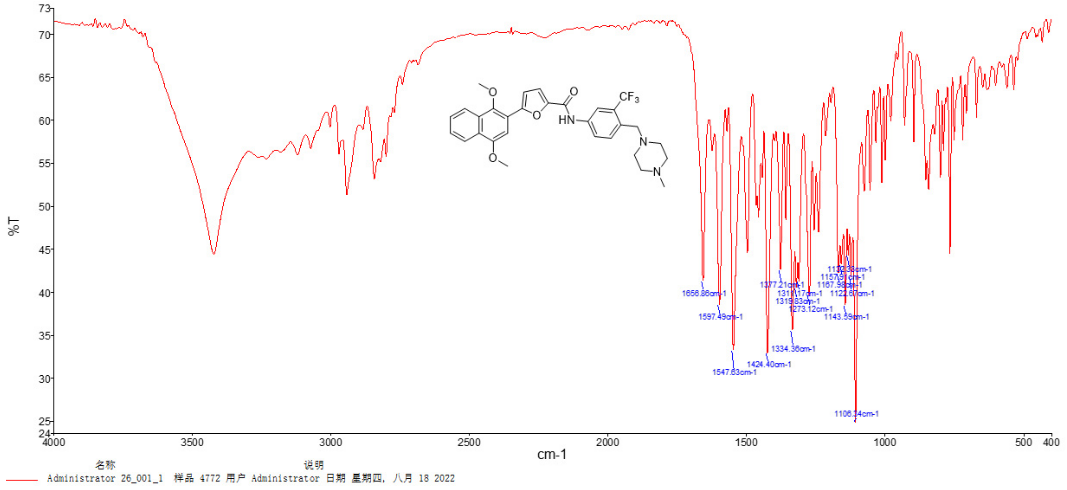

IR of compound **8h**

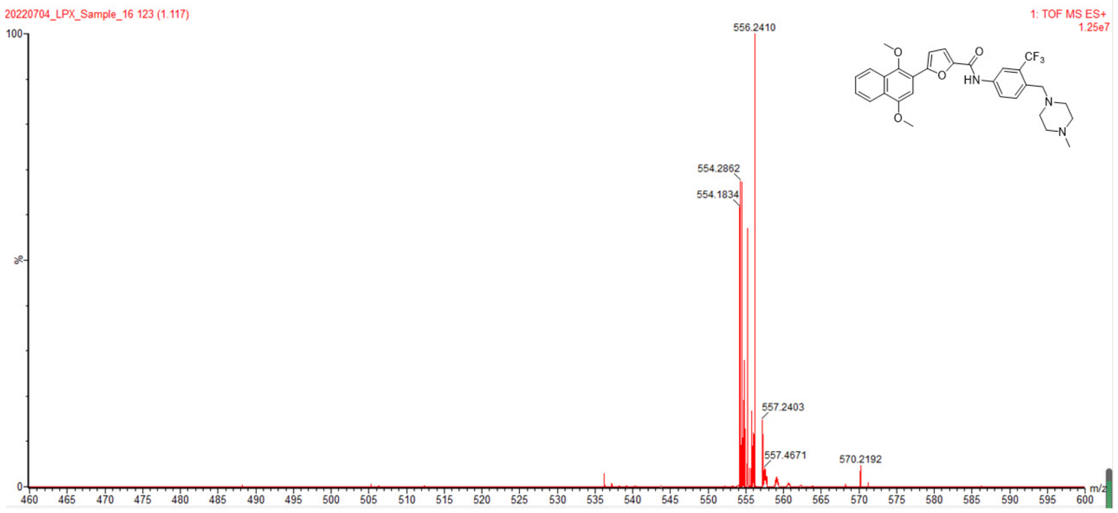

HRMS of compound **8h**

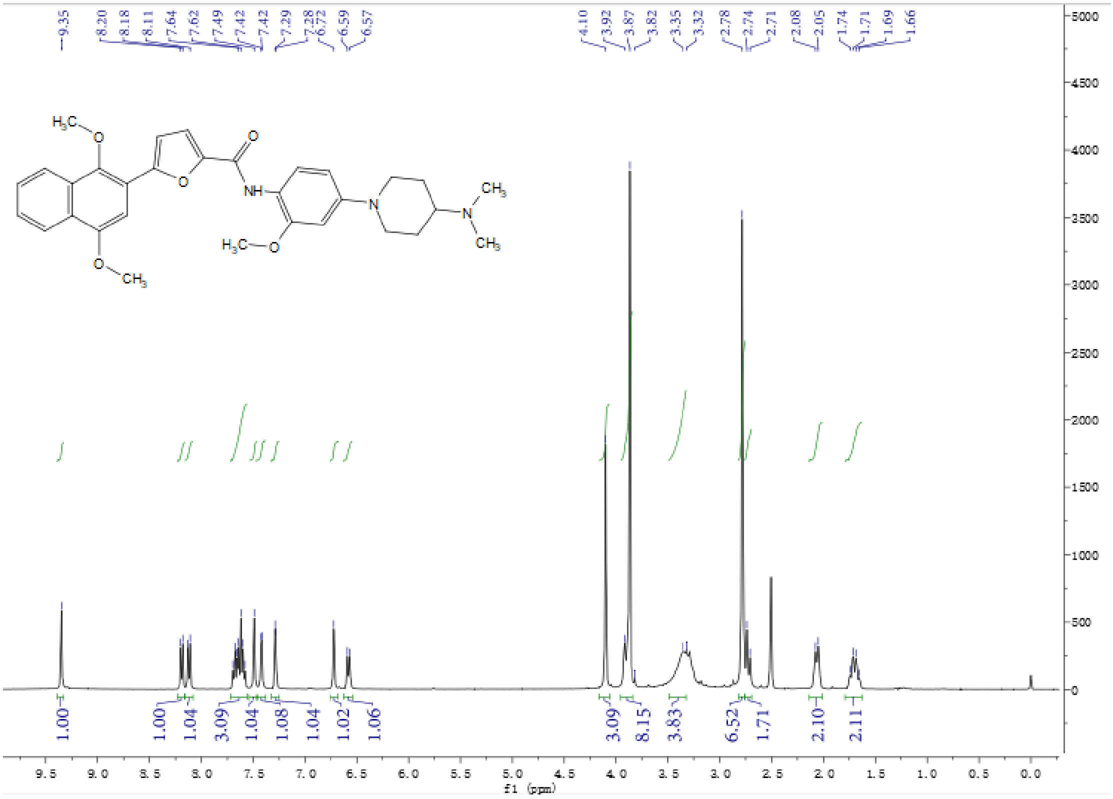

^{1}H-NMR of compound **8i**

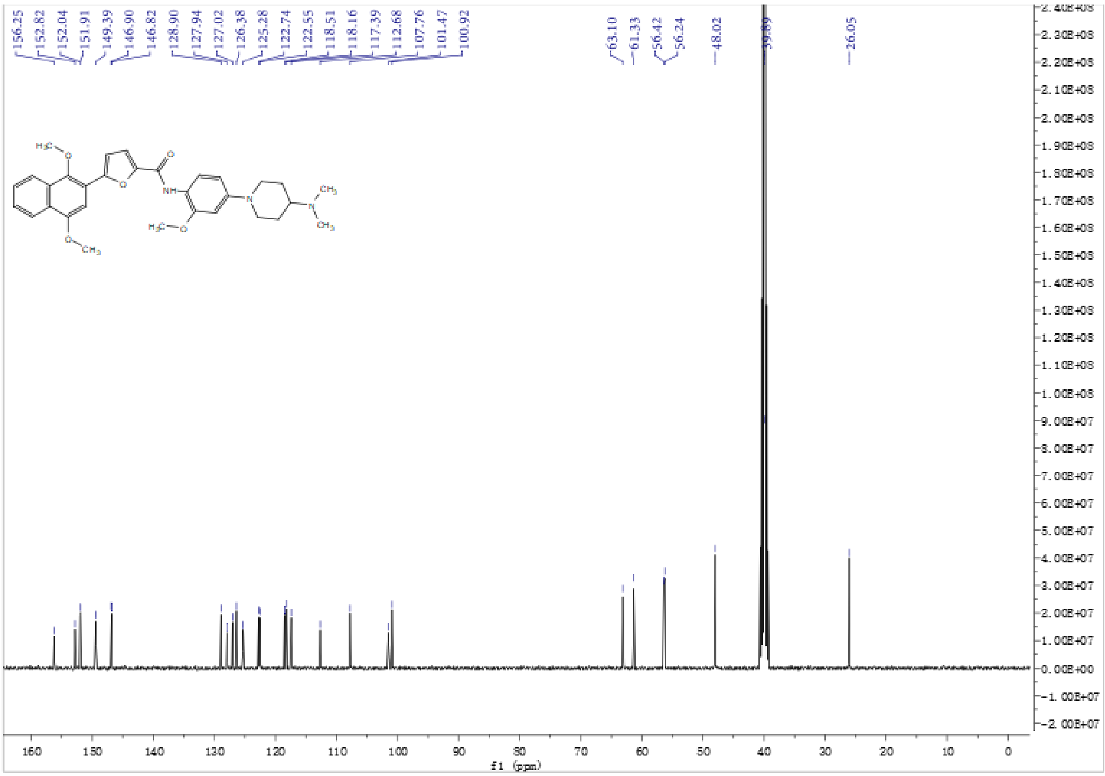

^{13}C-NMR of compound **8i**

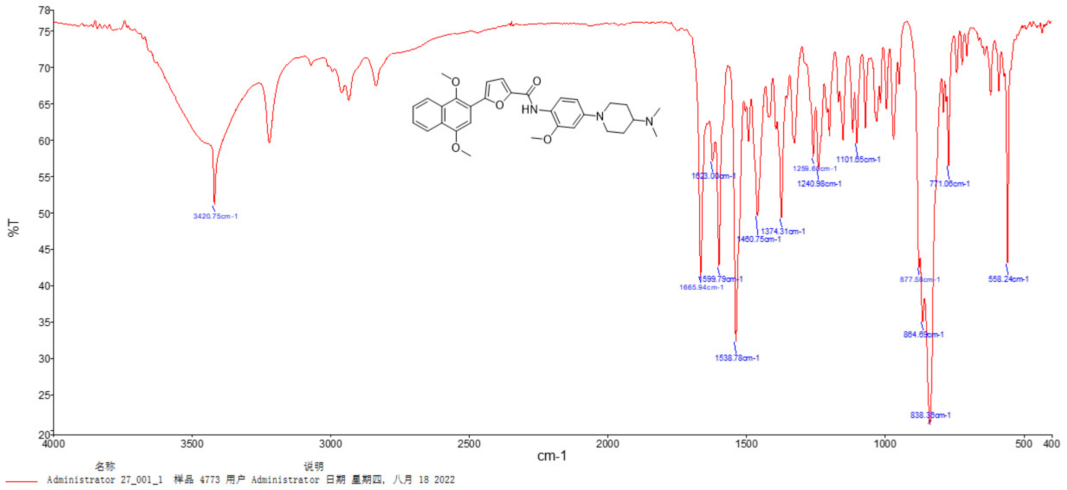

IR of compound **8i**

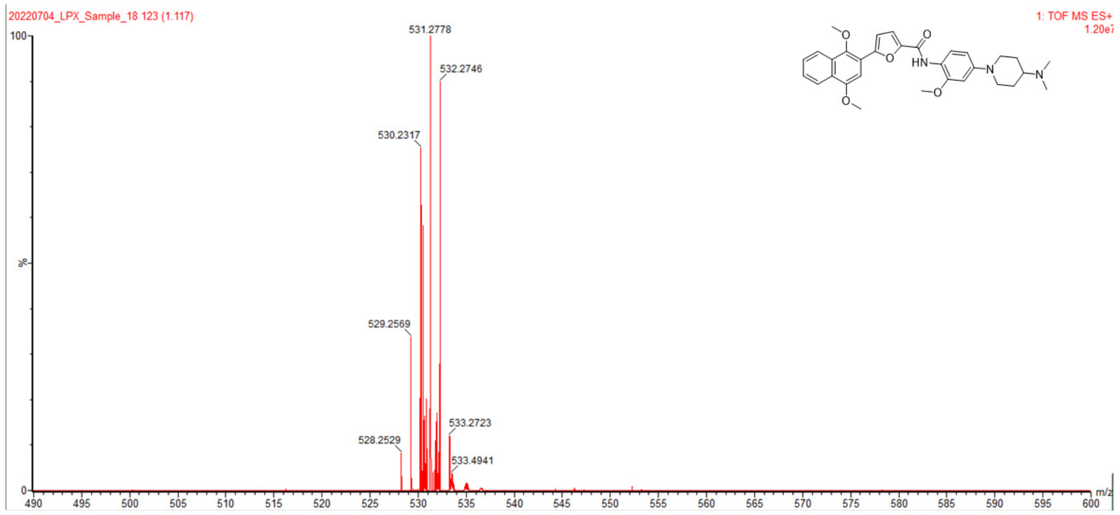

HRMS of compound**8i**

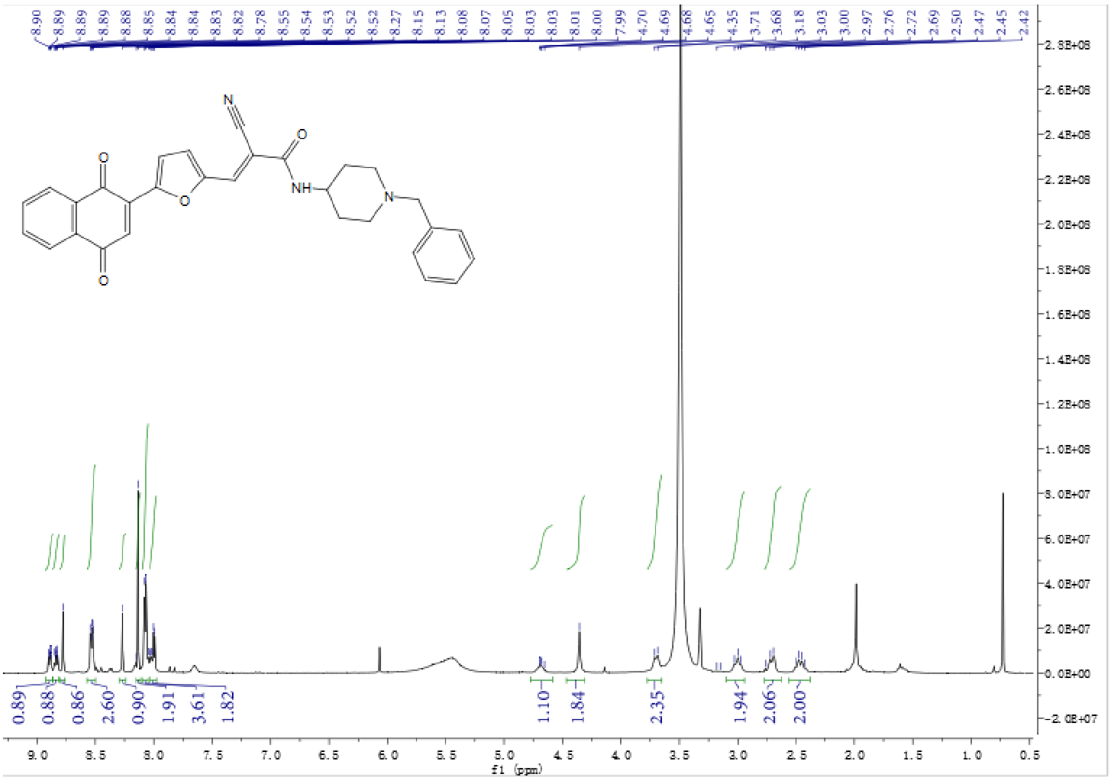

^{1}H-NMR of compound **9a**

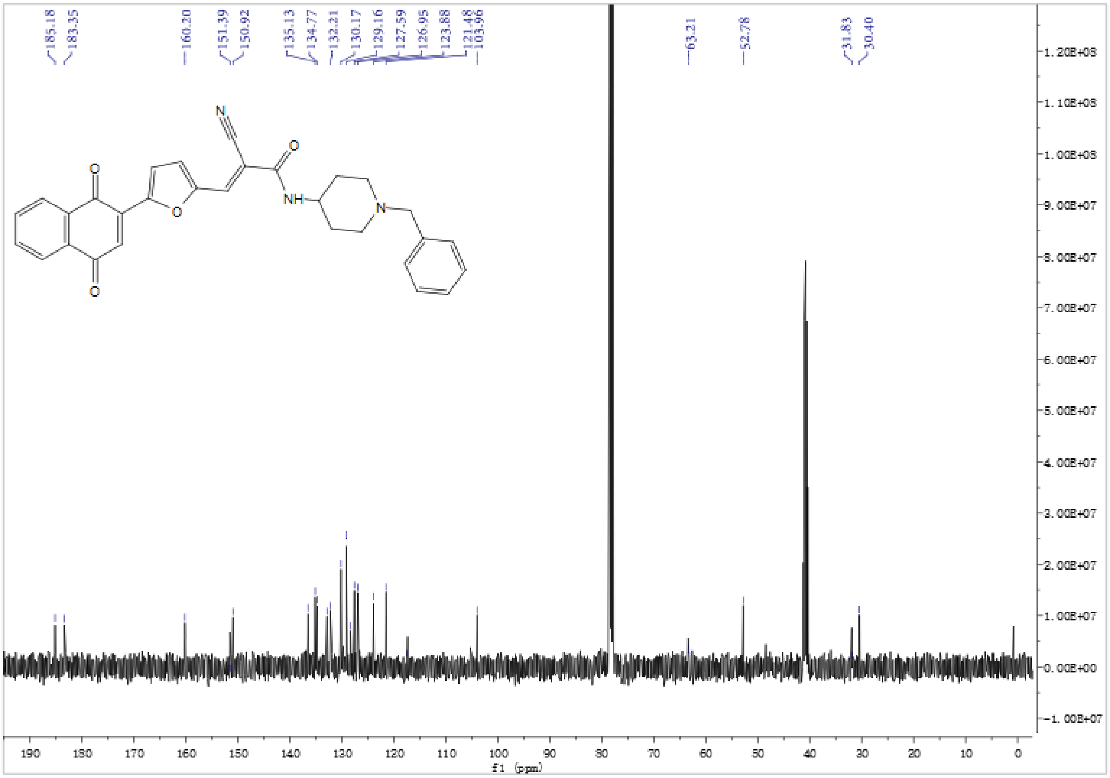

^{13}C-NMR of compound **9a**

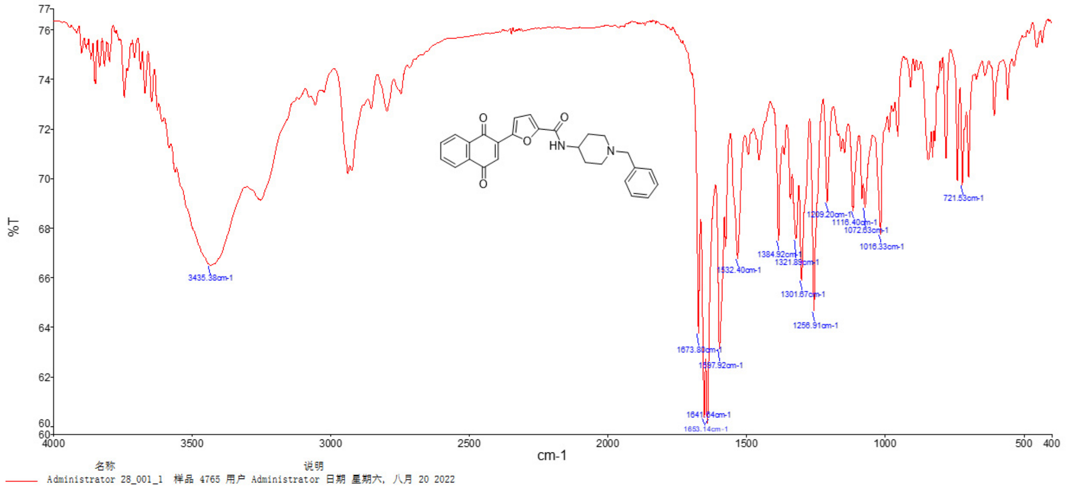

IR of compound **9a**

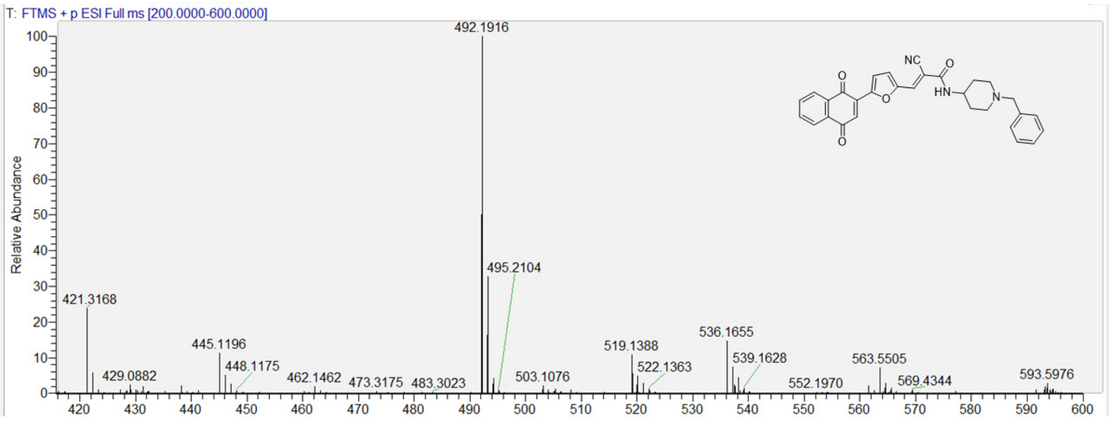

HRMS of compound **9a**

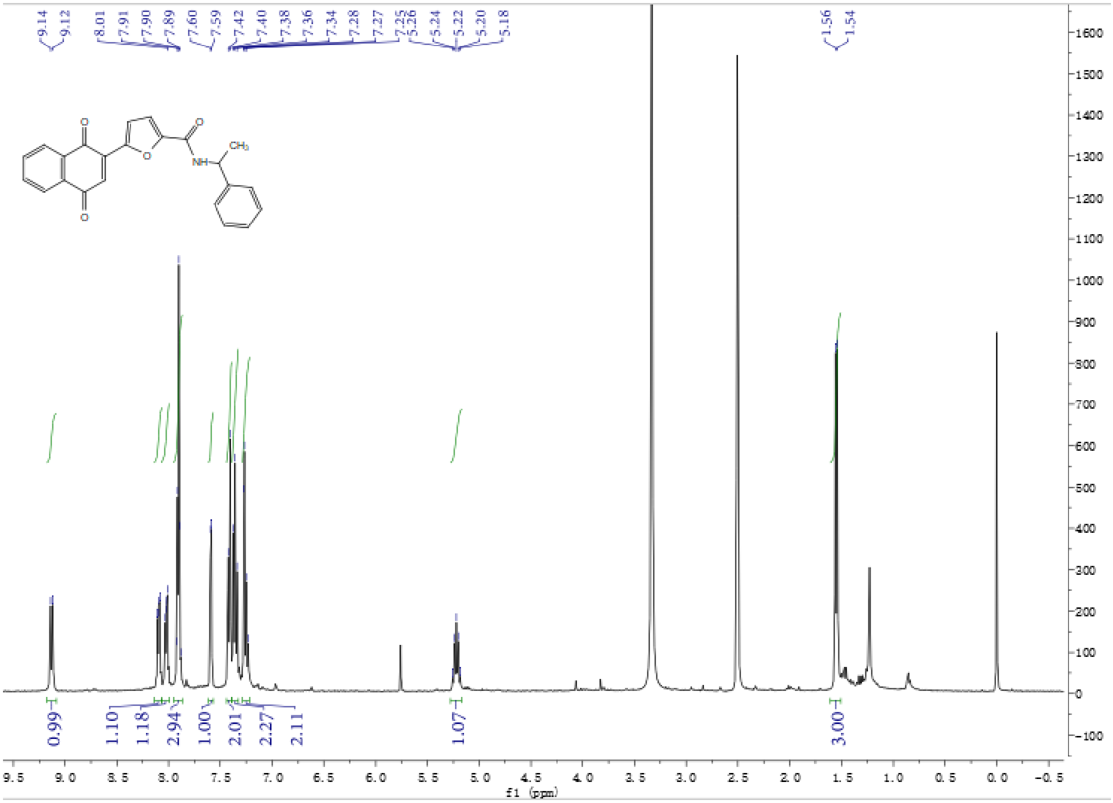

^{1}H-NMR of compound **9b**

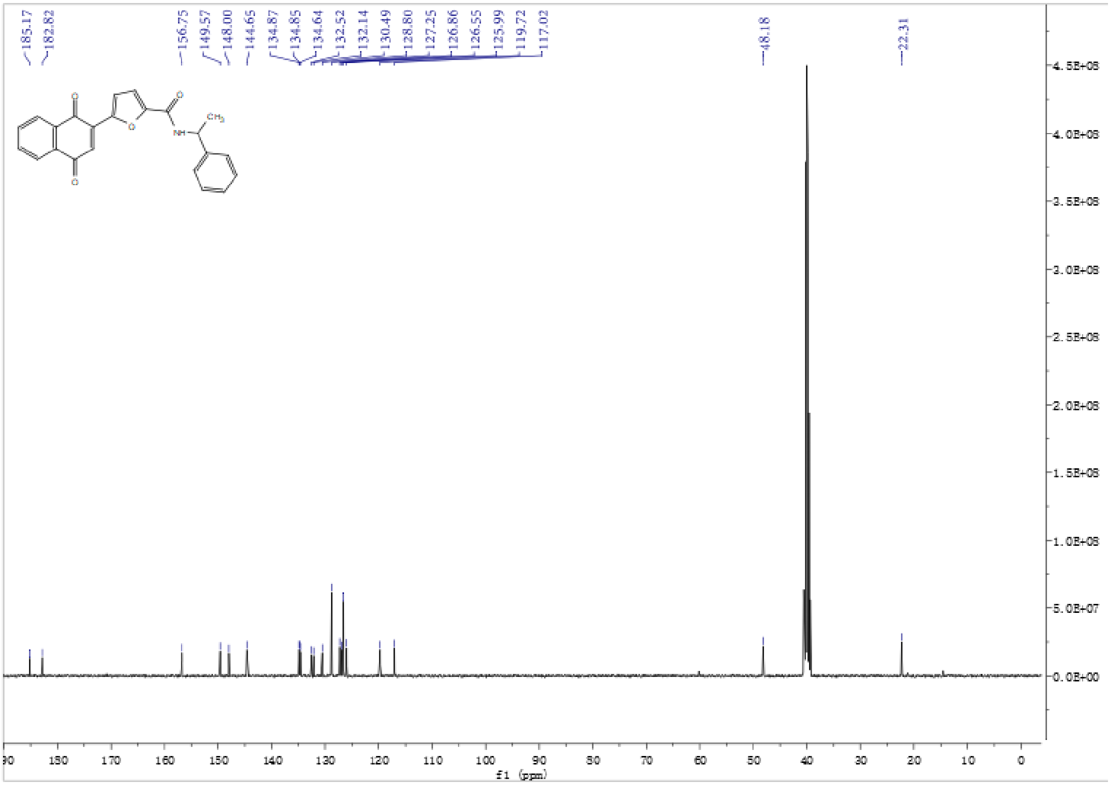

^{13}C-NMR of compound **9b**

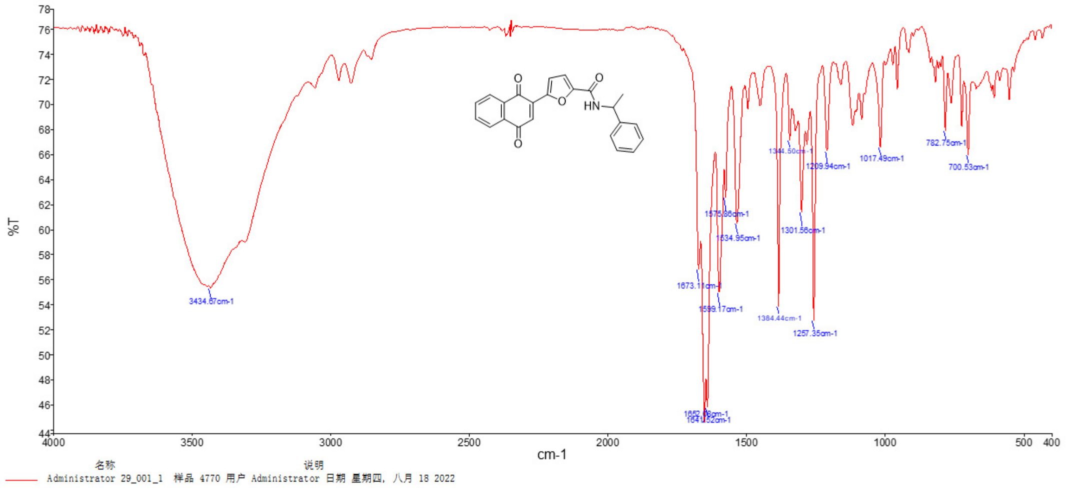

IR of compound **9b**

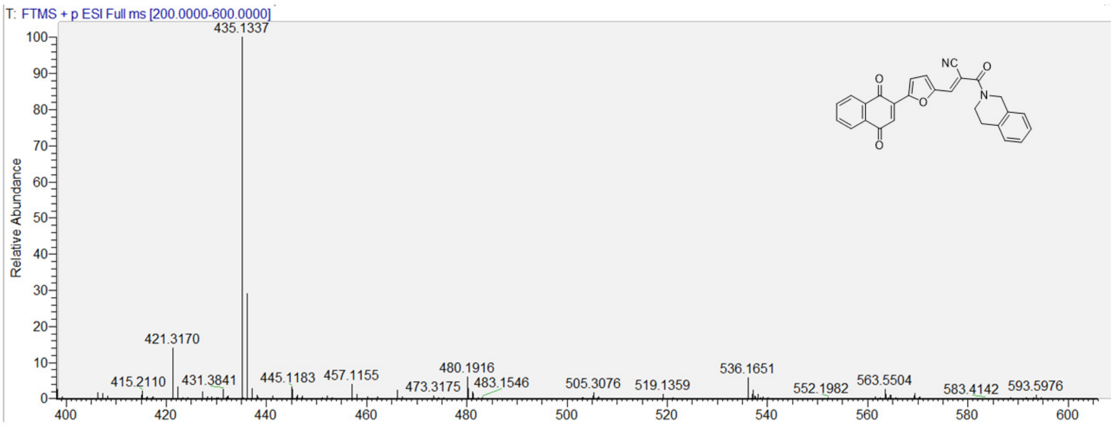

HRMS of compound **9b**

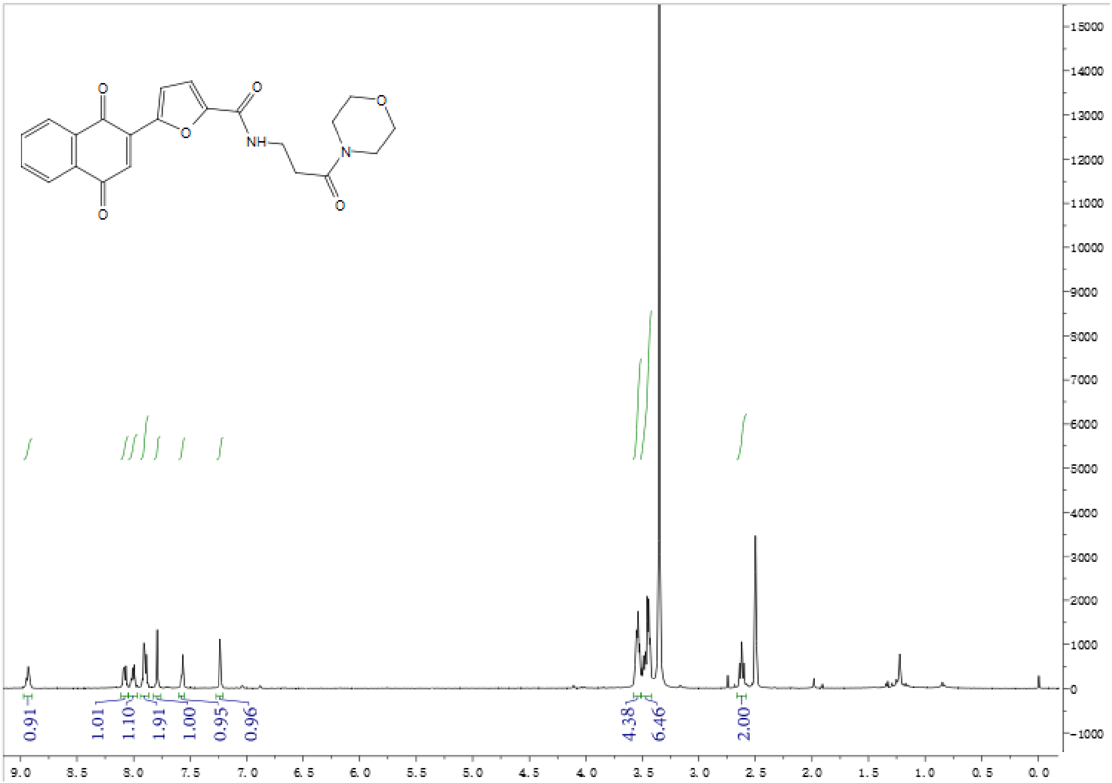

^{1}H-NMR of compound **9c**

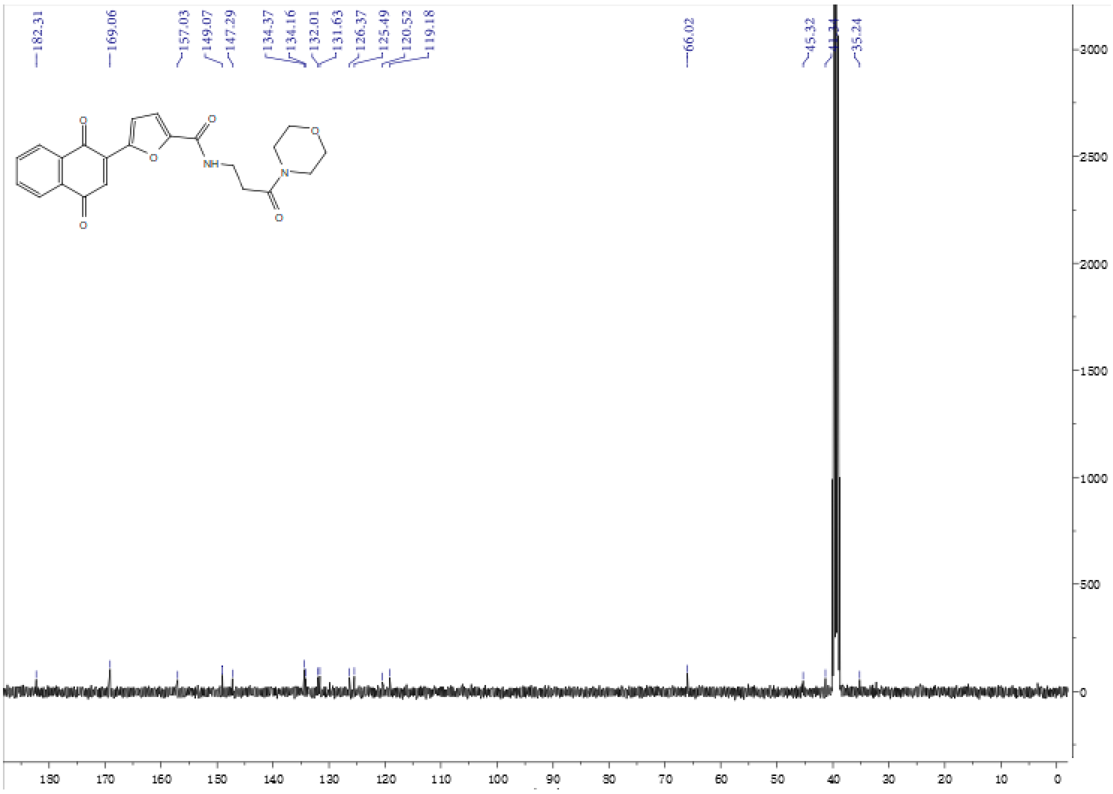

^{13}C-NMR of compound **9c**

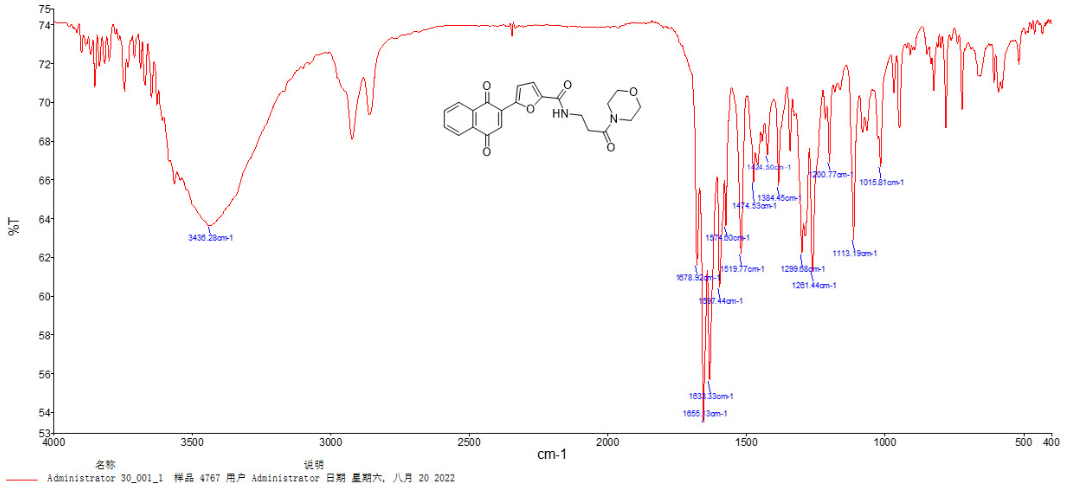

IR of compound **9c**

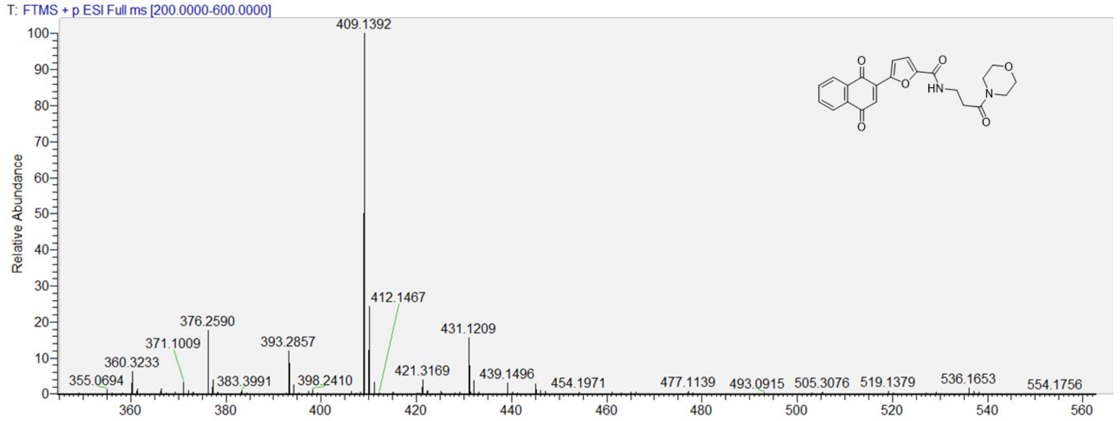

HRMS of compound **9c**
